# The Effect of New Thiophene-Derived Diphenyl Aminophosphonates on Growth of Terrestrial Plants

**DOI:** 10.3390/ma12122018

**Published:** 2019-06-24

**Authors:** Diana Rogacz, Jarosław Lewkowski, Marta Siedlarek, Rafał Karpowicz, Anna Kowalczyk, Piotr Rychter

**Affiliations:** 1 Faculty of Mathematics and Natural Science, Jan Długosz University in Częstochowa, 13/15 Armii Krajowej Av., 42-200 Częstochowa, Poland; diana.rogacz@gmail.com; 2 Department of Organic Chemistry, Faculty of Chemistry, University of Łódź, Tamka 12, 91-403 Łódź, Poland; mz.morawska@gmail.com (M.S.); rafal.karpowicz@chemia.uni.lodz.pl (R.K.); anak56@gmail.com (A.K.)

**Keywords:** *Avena sativa*, *Raphanus sativus*, persistent weeds, *Aliivibrio fischeri* test, *Heterocypris incongruens* test, ecotoxicology, OECD standard, thiophene-derived aminophosphonic diphenyl esters

## Abstract

The aim of this work was to evaluate the impact of the thiophene-derived aminophosphonates **1**–**6** on seedling emergence and growth of monocotyledonous oat (*Avena sativa*) and dicotyledonous radish (*Raphanus sativus* L.), and phytotoxicity against three persistent and resistant weeds (*Galinsoga parviflora* Cav., *Rumex acetosa* L., and *Chenopodium album*). Aminophosphonates **1**–**6** have never been described in the literature. The phytotoxicity of tested aminophosphonates toward their potential application as soil-applied herbicides was evaluated according to the OECD (Organization for Economic and Cooperation Development Publishing) 208 Guideline. In addition, their ecotoxicological impact on crustaceans *Heterocypris incongruens* and bacteria *Aliivibrio fischeri* was measured using the OSTRACODTOXKIT^TM^ and Microtox^®^ tests. Obtained results showed that none of the tested compounds were found sufficiently phytotoxic and none of them have any herbicidal potential. None of the tested compounds showed important toxicity against *Aliivibrio fischeri* but they should be considered as slightly harmful. Harmful impacts of compounds **1**–**6** on *Heterocypris incongruens* were found to be significant.

## 1. Introduction

Compounds bearing thiophene moiety are of increasing importance in various fields of science and technology [1]. Due to their multiple functional properties and chemical versatility, they are investigated in many aspects of material chemistry and engineering. The thiophene-based materials have found their application in organic electronics, where their semiconducting properties are exploited in thin film field-effect transistors and solar cells and in bioimaging, where their optical properties are utilized to monitor biological processes involving proteins and DNA.

It is believed that organic electronics will replace inorganic solid state electronics, owing to their flexibility, easy deposition of materials on large-area substrates, environmental-friendly production, and tunability of electronic properties via chemical synthesis [2]. Thiophene-derived compounds have been employed in a variety of devices, such as light-emitting transistors [3], lasers [4], electrochromic devices [5], and chemo and biosensors [6].

Apart from above, thiophene oligomers are characterized by fluorescence frequency, being tunable from blue to near infrared depending on molecular structure [7]. They are optically stable compounds that do not undergo photobleaching or blinking and display intense and persistent emission signals. These features make oligothiophene fluorophores capable of recognizing specific proteins inside live cells and live animals. For example, a series of thiophene-based fluorophores having a 4-sulfo-2,3,5,6-tetrafluorophenyl (STP) ester group were studied to demonstrate the tuning of the emission frequency by changing the oligomer size or adding one terminal SCH_3_ substituent [8].

Thiophene-based compounds remain a privileged scaffold in medicine [9]. For example, clopidogrel belongs to the thienopyridine class of antiplatelet agents [10]. Tiotropium bromide, which bears two thiophene rings, is a muscarinic receptor antagonist used in the management of chronic obstructive pulmonary disease [11]. Duloxetine is a serotonin-norepinephrine reuptake inhibitor that has the thiophene ring in its molecule [12] and rivaroxaban is an anticoagulant agent preventing blood clot formation [13].

Sulfur-containing compounds are widely used as plant protection agents and their use and biological properties were reported regarding their fungicidal, insecticidal, or herbicidal action [14,15,16,17,18,19]. They are widely used and applied either to weed foliage or to the soil where they are absorbed by roots and shoots of emerging seedlings [14,15,16,17,18,19].

Aminophosphonic derivatives are generally known to have moderate-to-strong phytotoxic action on higher plants [20]. Phosphonic analogues of phenylglycine were reported to exhibit their interesting herbicidal activity [20,21], and are used as plant growth regulators [20,21] or agrochemical fungicides [20,21]. Therefore, methods for their preparation are still intensively developed [22].

In our previous articles [23,24], we described the preparation and ecotoxicological as well as phytotoxicological properties of various dimethyl amino(2-thienyl)methylphosphonates. One of these, namely dimethyl *N*-(2-methylphenyl)amino(2-thienyl)methylphosphonate, was found to be the most promising as a prospective herbicide. Therefore, in search of better properties, we extended our studies to other thiophene-derived aminophosphonic esters, and herein we describe the synthesis of novel diphenyl amino(2-thienyl)methylphosphonates**1**–**6** and investigations on their phyto- and ecotoxicological properties. Their phytotoxicological properties were assessed by the seedling emergence and growth test on two model plants, dicotyledonous radish (*Raphanus sativus)* and monocotyledonous oat (*Avena sativa*). Their phytotoxicity was also tested on three popular and persistent dicotyledonous weeds: gallant soldier *(Galinsoga parviflora* Cav.), common sorrel *(Rumex acetosa* L.), and white goosefoot *(Chenopodium album* L.). Finally, fulfilling recommendations of the European Chemicals Agency (ECHA), we evaluated the ecotoxicological impact of the newly synthesized aminophosphonates on crustaceans *Heterocypris incongruens* (Ostaracodtoxkit™) and bacteria *Aliivibrio fischeri* (Microtox^®^).

## 2. Materials and Methods

### 2.1. Preparation of Aminophosphonates **1–6**

#### 2.1.1. General Information

All solvents (POCh, Gliwice, Poland) were routinely distilled and dried prior to use. Amines, diphenyl phosphite, and thiophene-2-carboxaldehyde (Aldrich, Poznań, Poland) were used as received.

Melting points were measured on a MelTemp II apparatus (Bibby Scientific Limited, Staffordshire, UK) and were not corrected. NMR (Nuclear Magnetic Resonance) spectra were recorded on an Avance III 600 MHz apparatus (Bruker, Billerica, MA, USA) operating at 600 MHz (^1^HNMR), 150 MHz (^13^CNMR), and 243 MHz (^31^PNMR). Elemental analyses were carried out in the Laboratory of Microanalysis, Faculty of Chemistry, University of Łódź, Poland.

#### 2.1.2. Procedure for Preparation of Diphenyl Amino(2-thienyl)methylphosphonates **1–6**

The procedure for synthesis of **1**–**6** was similar to the previously published method [23,24]. Thiophene-2-carboxaldehyde (12 mmol, 1.34 g) was dissolved in methanol (15 mL) and a solution of an appropriate toluidine or anisidine (12 mmol) in methanol (15 mL) was added. The mixture was stirred magnetically for 24 h at room temperature. Then, 2–3 g of anhydrous potassium carbonate was added, and after several minutes the mixture was filtrated and evaporated. Identities of obtained imines were confirmed by ^1^H NMR and they were used for further reactions without any purification.

Obtained imine (10 mmol) was dissolved in acetonitrile (15 mL) and a solution of diphenyl phosphite (10 mmol, 2.34 g, 1.91 mL) in acetonitrile (15 mL) was added. The obtained mixture was refluxed (at 80 °C) during a day and stirred at room temperature at night. The reaction lasted for 72 h. Then, the solvent was evaporated *in vacuo*. Crude products were purified by column chromatography on silica gel eluted with ethyl acetate: hexane 3:1. Scans of ^1^H, ^13^C, and ^31^P NMR spectra of all synthesized aminophosphonates **1–6** are shown in the Supplementary Materials Part 1.

*Diphenyl N-(2-methylphenyl)amino(2-thienyl)methylphosphonate* (**1**)

Yield = 71% (3.10 g) ecru crystals, mp: = 62–64 °C. ^1^H NMR (600 MHz, CDCl_3_): δ 7.29–7.26 (m, 6H, ArH); 7.18–7.14 (m, 2H, C_6_H_4_); 7.10–7.07 (m, 4H, ArH); 7.02–7.00 (m, 3H, ArH); 6.75 (approx. t, ^3^J_HH_ = 7.4 Hz, 1H, C_6_H_4_); 6.68 (d, ^3^J_HH_ = 7.9 Hz, 1H, C_6_H_4_); 5.48 (d, ^2^J_PH_ = 24.1 Hz, 1H, CHP); 4.58 (br. S, 1H, NH); 2.19 (s, 3H, CH_3_). ^31^P NMR (243 MHz, CDCl_3_): δ 13.33. ^13^C NMR (150 MHz, CDCl_3_): δ 150.6 (d, ^2^J_CP_ = 9.5 Hz, PO*C*_Ar_); 150.4 (d, ^2^J_CP_ = 9.7 Hz, PO*C*_Ar_); 143.8 (d, ^2^J_CP_ = 13.0 Hz, PC*C*_thioph_); 138.8 (C_Ar_); 130.6 (C_Ar_); 129.8 (C_Ar_); 127.4 (d, ^5^J_CP_ = 2.9 Hz, C^5^_thioph_); 127.2 (C_Ar_); 126.9 (d, ^3^J_CP_ = 7.3 Hz, C^3^_thioph_); 125.9 (d, ^4^J_CP_ = 4.1 Hz, C_thioph_); 125.5 (C_Ar_); 125.4 (C_Ar_); 123.6 (C_Ar_); 120.7 (d, ^3^J_CP_ = 4.1 Hz, C_Ar_); 120.5 (d, ^3^J_CP_ = 4.3 Hz, C_Ar_); 119.2 (C_Ar_); 111.6 (C_Ar_); 52.2 (d, ^1^J_CP_ = 159.9 Hz, CP); 17.5 (Ar-*C*H_3_). Elemental analysis: calculated for C_24_H_22_NO_3_PS: C, 66.19; H, 5.09; N, 3.22; S, 7.36. Found: C, 66.01; H, 5.23; N, 3.42; S, 7.23.

*Diphenyl N-(3-methylphenyl)amino(2-thienyl)methylphosphonate* (**2**)

Yield = 94% (4.10 g) ecru crystals, mp: = 124–126 °C. ^1^H NMR (600 MHz, CDCl_3_): δ 7.29–7.24 (m, 6H, ArH); 7.17–7.14 (m, 2H, ArH); 7.09–7.05 (m, 3H, ArH); 7.00–6.98 (m, 3H, ArH); 6.62 (d, ^3^J_HH_ = 7.4 Hz, 1H, C_6_H_4_); 6.55–6.54 (m, 1H, C_6_H_4_); 6.52 (dd, ^3^J_HH_ = 7.9 and ^4^J_HH_ = 2.3 Hz, 1H, C_6_H_4_); 5.42 (d, ^2^J_PH_ = 24.1 Hz, 1H, CHP); 4.61 (broad s, 1H, NH); 2.26 (s, 3H, CH_3_). ^31^P NMR (243 MHz, CDCl_3_): δ 13.33. ^13^C NMR (150 MHz, CDCl_3_): δ 150.4 (d, ^2^J_CP_ = 9.8 Hz, PO*C*_Ar_); 150.3 (d, ^2^J_CP_ = 9.8 Hz, PO*C*_Ar_); 145.7 (d, ^2^J_CP_ = 13.2 Hz, PC*C*_thioph_); 139.3 (C_Ar_); 138.6 (C_Ar_); 129.8 (d, ^2^J_CP_ = 2.3 Hz, C_Ar_); 129.3 (C_Ar_); 127.4 (d, ^5^J_CP_ = 2.9 Hz, C^5^_thioph_); 127.1 (d, ^3^J_CP_ = 7.1 Hz, C^3^_thioph_); 125.9 (d, ^4^J_CP_ = 3.9 Hz, C_thioph_); 125.5 (C_Ar_); 125.4 (C_Ar_); 120.8 (d, ^3^J_CP_ = 4.3 Hz, C_Ar_); 120.5 (d, ^3^J_CP_ = 4.4 Hz, C_Ar_); 120.4 (C_Ar_); 115.2 (C_Ar_); 111.3 (C_Ar_); 52.1 (d, ^1^J_CP_ = 161.5 Hz, CP); 21.7 (Ar-*C*H_3_). Elemental analysis: calculated for C_24_H_22_NO_3_PS: C, 66.19; H, 5.09; N, 3.22; S, 7.36. Found: C, 66.30; H, 5.24; N, 3.42; S, 7.15.

*Diphenyl N-(4-methylphenyl)amino(2-thienyl)methylphosphonate* (**3**)

Yield = 80% (3.50 g) ecru crystals, mp: = 149–151 °C. ^1^H NMR (600 MHz, CDCl_3_): δ 7.29–7.24 (m, 6H, ArH); 7.17–7.12 (m, 2H, ArH); 7.10–7.09 (m, 2H, ArH); 7.00–6.97 (m, 5H, ArH); 6.65–6.62 (m, 2H, ArH); 5.38 (dd, ^2^J_PH_ = 24.0 and ^3^J_HH_ = 8.1 Hz, 1H, CHP); 4.54 (dd, ^3^J_PH_ = ^3^J_HH_ = 8.0 Hz, 1H, NH); 2.24 (s, 3H, CH_3_). ^31^P NMR (243 MHz, CDCl_3_): δ 13.43. ^13^C NMR (150 MHz, CDCl_3_): δ 150.5 (d, ^2^J_CP_ = 9.8 Hz, PO*C*_Ar_); 150.3 (d, ^2^J_CP_ = 9.7 Hz, PO*C*_Ar_); 143.4 (d, ^2^J_CP_ = 13.8 Hz, PC*C*_thioph_); 138.7 (C_Ar_); 130.0 (C_Ar_); 129.8 (d, ^4^J_CP_ = 3.1 Hz, C_Ar_); 128.8 (C_Ar_); 127.4 (d, ^5^J_CP_ = 2.7 Hz, C^5^_thioph_); 127.0 (d, ^3^J_CP_ = 7.6 Hz, C^3^_thioph_); 125.9 (d, ^4^J_CP_ = 4.1 Hz, C_thioph_); 125.5 (C_Ar_); 125.4 (C_Ar_); 120.8 (d, ^3^J_CP_ = 4.2 Hz, C_Ar_); 120.5 (d, ^3^J_CP_ = 3.9 Hz, C_Ar_); 114.5 (C_Ar_); 52.4 (d, ^1^J_CP_ = 161.5 Hz, CP); 20.6 (Ar-*C*H_3_). Elemental analysis: calculated for C_24_H_22_NO_3_PS: C, 66.19; H, 5.09; N, 3.22; S, 7.36. Found: C, 66.33; H, 5.16; N, 3.37; S, 7.33.

*Diphenyl N-(2-methoxyphenyl)amino(2-thienyl)methylphosphonate* (**4**)

Yield = 89% (4.00 g) ecru crystals, mp: = 91–93 °C. ^1^H NMR (600 MHz, CDCl_3_): δ 7.28–7.24 (m, 6H, ArH); 7.16–7.13 (m, 2H, ArH); 7.09–7.08 (m, 2H, ArH); 7.04–7.02 (m, 2H, ArH); 6.99–6.98 (m, 1H, ArH); 6.83–6.80 (m, 2H, ArH); 6.76–6.74 (m, 1H, ArH); 6.67–6.66 (m, 1H, ArH); 5.41 (d, ^2^J_PH_ = 23.7 Hz, 1H, CHP); 3.86 (s, 3H, OCH_3_). ^31^P NMR (243 MHz, CDCl_3_): δ 13.37. ^13^C NMR (150 MHz, CDCl_3_): δ 150.5 (d, ^2^J_CP_ = 9.9 Hz, PO*C*_Ar_); 150.4 (d, ^2^J_CP_ = 9.3 Hz, PO*C*_Ar_); 147.6 (C_Ar_); 138.7 (C_Ar_); 135.8 (d, ^2^J_CP_ = 13.1 Hz, PC*C*_thioph_); 129.8 (d, ^4^J_CP_ = 4.9 Hz, C_Ar_); 127.4 (d, ^5^J_CP_ = 2.6 Hz, C^5^_thioph_); 127.0 (d, ^3^J_CP_ = 7.3 Hz, C^3^_thioph_); 125.9 (d, ^4^J_CP_ = 4.0 Hz, C_thioph_); 125.4 (d, ^4^J_CP_ = 4.1 Hz, C_Ar_); 121.2 (C_Ar_); 120.7 (d, ^3^J_CP_ = 4.2 Hz, C_Ar_); 120.6 (d, ^3^J_CP_ = 4.3 Hz, C_Ar_); 118.8 (C_Ar_); 111.5 (C_Ar_); 110.1 (C_Ar_); 55.7 (Ar-O*C*H_3_); 52.0 (d, ^1^J_CP_ = 160.6 Hz, CP). Elemental analysis: calculated for C_24_H_22_NO_4_PS: C, 63.85; H, 4.91; N, 3.10; S, 7.10. Found: C, 63.63; H, 4.97; N, 3.25; S, 7.07.

*Diphenyl N-(3-methoxyphenyl)amino(2-thienyl)methylphosphonate* (**5**)

Yield = 75% (3.40 g) ecru crystals, mp: = 95–97 °C. ^1^H NMR (600 MHz, CDCl_3_): δ 7.29–7.24 (m, 6H, ArH); 7.17–7.12 (m, 2H, ArH); 7.10–7.07 (m, 3H, ArH); 7.00–6.98 (m, 3H, ArH); 6.36 (dd, ^3^J_HH_ = 8.2 and ^4^J_HH_ = 2.3 Hz, 1H, C_6_H_4_); 6.33 (dd, ^3^J_HH_ = 8.0 and ^4^J_HH_ = 2.3 Hz, 1H, C_6_H_4_); 6.27–6.26 (m, 1H, ArH); 5.40 (dd, ^2^J_PH_ = 24.1 and ^3^J_HH_ = 8.7 Hz, 1H, CHP); 4.69 (dd, ^3^J_PH_ = ^3^J_HH_ = 8.3 Hz, 1H, NH); 3.74 (s, 3H, OCH_3_). ^31^P NMR (243 MHz, CDCl_3_): δ 13.21. ^13^C NMR (150 MHz, CDCl_3_): δ 160.9 (C_Ar_); 150.4 (d, ^2^J_CP_ = 9.8 Hz, PO*C*_Ar_); 150.3 (d, ^2^J_CP_ = 9.8 Hz, PO*C*_Ar_); 147.2 (d, ^2^J_CP_ = 13.7 Hz, PC*C*_thioph_); 138.4 (C_Ar_); 130.3 (C_Ar_); 129.9 (d, ^4^J_CP_ = 4.0 Hz, C_Ar_); 129.6 (C_Ar_); 127.5 (d, ^5^J_CP_ = 3.0 Hz, C^5^_thioph_); 127.1 (d, ^3^J_CP_ = 7.6 Hz, C^3^_thioph_); 126.0 (d, ^4^J_CP_ = 4.0 Hz, C_thioph_); 125.6 (C_Ar_); 125.5 (C_Ar_); 121.2 (C_Ar_); 120.8 (d, ^3^J_CP_ = 4.2 Hz, C_Ar_); 120.5 (d, ^3^J_CP_ = 3.9 Hz, C_Ar_); 115.5 (C_Ar_); 107.1 (C_Ar_); 104.7 (C_Ar_); 100.4 (C_Ar_); 55.2 (Ar-O*C*H_3_); 52.0 (d, ^1^J_CP_ = 161.2 Hz, CP). Elemental analysis: calculated for C_24_H_22_NO_4_PS: C, 63.85; H, 4.91; N, 3.10; S, 7.10. Found: C, 63.93; H, 4.97; N, 3.27; S, 7.04.

*Diphenyl N-(4-methoxyphenyl)amino(2-thienyl)methylphosphonate* (**6**)

Yield = 74% (3.35 g) ecru crystals, mp: = 87–91 °C. ^1^H NMR (600 MHz, CDCl_3_): δ 7.29–7.23 (m, 6H, ArH); 7.17–7.10 (m, 4H, ArH); 7.00–6.98 (m, 3H, ArH); 6.77–6.76 (m, 2H, C_6_H_4_); 6.69–6.67 (m, 2H, C_6_H_4_); 5.32 (d, ^2^J_PH_ = 23.7 Hz, 1H, CHP); 4.41 (broad s, 1H, NH); 3.73 (s, 3H, CH_3_). ^31^P NMR (243 MHz, CDCl_3_): δ 13.52. ^13^C NMR (150 MHz, CDCl_3_): δ 156.1 (C_Ar_); 153.5 (C_Ar_); 150.5 (d, ^2^J_CP_ = 9.9 Hz, PO*C*_Ar_); 150.3 (d, ^2^J_CP_ = 9.8 Hz, PO*C*_Ar_); 139.7 (d, ^2^J_CP_ = 14.9 Hz, PC*C*_thioph_); 129.9 (d, ^4^J_CP_ = 3.0 Hz, C_Ar_); 129.6 (C_Ar_); 128.4 (C_Ar_); 127.4 (d, ^5^J_CP_ = 2.3 Hz, C^5^_thioph_); 127.1 (d, ^3^J_CP_ = 7.6 Hz, C^3^_thioph_); 126.0 (d, ^4^J_CP_ = 4.0 Hz, C_thioph_); 125.6 (C_Ar_); 125.5 (C_Ar_); 120.8 (d, ^3^J_CP_ = 3.7 Hz, C_Ar_); 120.5 (d, ^3^J_CP_ = 4.3 Hz, C_Ar_); 120.4 (C_Ar_); 116.0 (C_Ar_); 115.5 (C_Ar_); 115.0 (C_Ar_); 55.8 (Ar-O*C*H_3_); 53.2 (d, ^1^J_CP_ = 161.8 Hz, CP). Elemental analysis: calculated for C_24_H_22_NO_4_PS: C, 63.85; H, 4.91; N, 3.10; S, 7.10. Found: C, 63.46; H, 5.06; N, 3.04; S, 6.96.

### 2.2. Plant Growth Test of Aminophosphonates 1–6

The plant growth test of diphenyl amino(2-thienyl)methylphosphonates **1**–**6** was performed in laboratory conditions following the OECD 208 Guideline Terrestrial Plants Growth Test [25]. Inhibition ratio (IR) was calculated according to the method reported by Wang et al. [26], as well as Pawłowska and Biczak [27]. This method has already been described by other authors [23,24,28,29,30,31], and the detailed procedure is fully described in the Supplementary Materials Part 2.

### 2.3. Pigment Assay

Photosynthetic pigment content was determined according to the method reported by Oren et al. [32]. The procedure is described in detail in the Supplementary Materials Part 2.

### 2.4. Determination of Herbicidal Activity

The weed growth test treated with aminophosphonates **1**–**6** as potential soil-applied herbicides was performed using three widely occurring weeds, namely gallant soldier (*Galinsoga parviflora* Cav.), common sorrel (*Rumex acetosa* L.), and white goosefoot *(Chenopodium album* L.). The weed test was carried out in the same way as the plant growth test at the following concentrations: 100, 400, and 1000 mg/kg of soil dry weight.

Ratings were assigned based on scales from the European Weed Research Council [33,34,35]. The procedure is described in detail in the Supplementary Materials Part 2.

### 2.5. Microtox^®^ Toxicity Assay

Detailed procedure of Microtox^®^ Toxicity Assay has been described by the authors in our previous study [36]. The method is based on the analysis of light emission reduction of luminescent bacteria (*Aliivibrio fischeri*) under toxic stress. The tests were carried out in a Microtox^®^ M500 analyzer according to the 1992 Microtox^®^ Manual. The Microtox^®^ Solid-Phase Test (MSPT) was adopted based on the report from Doe et al. [37]. The procedure is described in detail in the Supplementary Materials Part 2.

### 2.6. Ostracod Test Kit

Ecotoxicity evaluation of synthesized compounds was performed in a short-term contact test using the Ostracodtoxkit F™ provided by MicroBiotests Inc. (Gent, Belgium). This direct sediment contact bioassay was performed in multi well test plates using neonates of the benthic ostracod crustacean *Heterocypris incongruens* hatched from cysts [38]. The procedure is described in detail in the Supplementary Materials Part 2.

### 2.7. Statistical Analysis

The significance of the obtained results was evaluated using the analysis of variance (ANOVA). The least significant difference (LSD) values at a confidence level of 95% were computed using the Tukey test. Moreover, the mean standard deviations were determined and plotted in diagrams. Statistical analysis was performed with STATISTICA 13.3 software.

## 3. Results and Discussion

### 3.1. Preparation of Thiophene-Derived Aminophosphonates **1–6**

Schiff bases were synthesized following the published and commonly known procedure [23,24]. This procedure produced imines, which were isolated, their identities were verified by the 1H NMR spectroscopy, and they were used for further conversions without any purification.

**Scheme 1:**
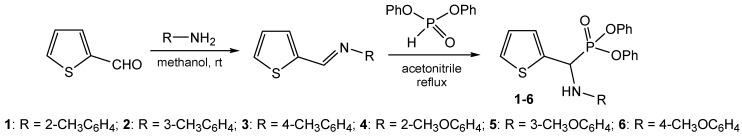
Synthesis of thiophene-derived aminophosphonates **1**–**6**.

Aminophosphonates **1**–**6** were synthesized via the slightly modified aza-Pudovik reaction—the addition of diphenyl phosphite to the azomethine bond of corresponding Schiff bases in boiling acetonitrile for 72 h. After the workup described in the Experimental Section, resulting aminophosphonates **1**–**6** were obtained in 60–70% yields. (Scheme 1)

Their identities have been confirmed by means of the ^1^H, ^13^C, and ^31^P NMR spectroscopy. The ^1^H NMR spectra of compounds **1**–**6** showed a doublet of a H-C-P (hydrogen-carbon-phosphorus) group shift around 5 ppm and a coupling constant around 24 Hz. Typical signals for 2-substituted thiophenes, i.e., three multiples of protons H^5^, H^4^, and H^3^ between 7.3 and 7.0 ppm, were also observed. The ^13^C NMR spectra showed a diagnostic doublet in the range of 53–51 ppm of a coupling constant oscillating between 159–162 Hz corresponding to a CP bond, which is also typical for aminophosphonic systems. Their purity was confirmed by the elemental analysis and by melting point measurements. Scans of ^1^H, ^13^C, and ^31^P NMR spectra of all synthesized aminophosphonates 1–6 are shown in the Supplementary Materials Part 1.

Compounds **1**–**6** were then investigated in terms of their phyto- and ecotoxicological properties.

### 3.2. Growth Inhibition of Shoot Height, Root Length, and Fresh Matter

The percentage growth inhibition (GI%) values of shoot height, root length, and fresh matter of both plants exposed to aminophosphonates **1**–**6** are presented in Table 1 and Table 2.

#### 3.2.1. Yield (Fresh Matter) and Shoot Height Changes

Increasing concentration of all tested aminophosphonates **1**–**6** did not affect significantly the shoot height and fresh mass of oat (*Avena sativa*) seedlings (Table 1).

Harmful effect of diphenyl N-(methylphenyl)amino(2-thienyl)methylphosphonates (**1**–**3**) on fresh mass of radish (*Raphanus sativus*) was in the following order: **1** > **2** > **3** (*o-*, *m-*, and *p-*substitution, respectively). Diphenyl *N*-(2-methylphenyl)amino(2-thienyl)methylphosphonate (**1**) demonstrated 48, 54, and 63% growth inhibition of radish fresh matter for concentrations of 400, 800, and 1000 mg/kg of soil dry weight (kg s.d.w.,) respectively (Table 2). For comparison, compounds **2** and **3** at concentrations of 800 mg and 1000 mg/kg s.d.w caused much less growth inhibition of radish fresh matter: 26–49% and 22–47%, respectively.

Values for shoot height inhibition demonstrated clearly that only aminophosphonates **1** and **2** had a harmful impact on the radish shoot height. GI% values for radish shoot height at the highest concentration used were 66.9 and 56.2% (Table 2) for compounds **1** and **2**, respectively, which showed the slightly higher toxicity of the *o*-methylphenyl-substituted compound **1**. The *p*-methylphenyl-substituted compound **3** was found to be practically harmless for radish, as GI% values were not higher than 4%.

Asimilartrend of harmful effect on radish fresh matter was found for the diphenyl *N*-(methoxyphenyl)-amino(2-thienyl)methylphosphonates (**4**–**6**). The highest toxicity was demonstrated by *o*-methoxyphenyl-substituted aminophosphonate **4**,and at concentrations of 400, 800, and 1000 mg/kg s.d.w., the fresh matter inhibition was, respectively, 42, 49, and 56%. Compounds **5** and **6** (*meta* and *para* substituted) showed lower toxicity to radish and at the highest concentration of 1000 mg/kg s.d.w., aminophosphonate **5** caused 44% GI, and compound **6** caused 41% GI. Similar trends were also observed in a case of inhibitive action of aminophosphonates **4**–**6** on radish shoot height. The *o*-methoxyphenyl derivative **4** was the most harmful, giving a GI% of 66.9% at 1000 mg/kg s.d.w. The *m*-methoxyphenyl analogue was less toxic, causing GI% of 55.2% at 1000 mg/kg s.d.w., whereas the *p*-methoxyphenyl isomer was found to be nearly harmless for radish shoot height, as GI% values did not exceed 4.5% for all tested concentrations.

Aminophosphonates bearing a methylphenyl moiety (**1**–**3**) were slightly more toxic for fresh matter and shoot height for both tested plants than the compounds bearing a methoxyphenyl moiety (**4**–**6**).

#### 3.2.2. Root Length Changes

The tendency of negative impact on oat and radish roots was similar to tendencies of impact on growth inhibition of fresh matter and shoot height. Regardless of the substitution of an amine group, methylphenyl substituted compounds **1**–**3**, or methoxyphenyl substituted compounds **4**–**6**, caused toxicity to decreasing degrees in the following order: **1** > **2** > **3** and **4** > **5** > **6** (*ortho*, *meta*, and *para*, respectively)

Aminophosphonate **1**, when tested in the highest concentration (1000 mg/kg s.d.w.), resulted in roots inhibition of *A*.*sativa* of 69%. A slightly lower value was determined for a thiophene derivative **2** bearing the *m*-methylphenyl moiety, with GI% of 63%, while the lowest was for the *para*-substituted compound **3** (GI% = 31%). The*o*-methoxyphenyl derivative **4** at the highest concentration caused GI% of oat root length inhibition of 53.7%. The lowest toxicity was caused by the aminophosphonate **5** (GI% = 29%), and the *p*-methoxyphenyl isomer (**6**) was found to be least toxic for oat roots (GI% = 22% at 1000 mg/kg s.d.w.).

The toxicity for radish roots showed a very similar trend. Among *N*-(methylphenyl)amino derivatives, diphenyl *N*-(2-methylphenyl)amino(2-thienyl)methylphosphonate (**1**) demonstrated the highest toxicity at the whole concentration range. At the lowest concentration (100 mg/kg s.d.w.), it showed inhibition of *R*. *sativus* root development of 49%. The lowest toxicity against radish roots was found for the *p*-methoxyphenyl derivative 6, giving GI% of 22.9% at the highest concentration (1000 mg/kg s.d.w.). Radish was found to be much more sensitive against newly synthesized compounds than oat. It is worth noting that for all inhibition biomarkers (fresh matter, shoot height, root length), the toxicity trends depend not only on the nature of a substituent (methylphenyl or methoxyphenyl), but also on the isomerism (*ortho*, *meta*, or *para* substitution). The highest toxicity was demonstrated for the thiophene derivatives bearing an *o*-methylphenyl (**1**) and an *o*-methoxyphenyl (**4**) moiety; less toxicity was determined for *meta*, while the lowest was for *para* substituted compounds.

**Figure 1:**
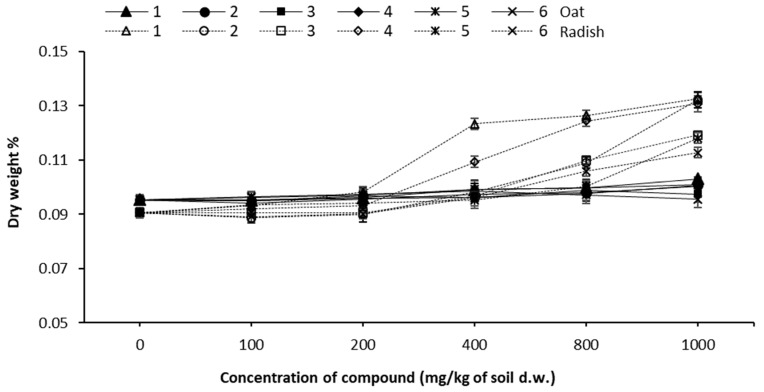
Changes of dry weight of treated oat and radish plants, expressed as percentages of the values in untreated plants **1**–**6** (control plants = 100% dry weight). Solid lines represent changes of oat dry weight. Dotted lines represent changes of radish dry weight.

The level of dry matter of *A*. *sativa* treated with thiophene derivatives **1**–**6** in soil were comparable to the control plants (Figure 1).

Growing concentration of applied xenobiotics in soil caused a rapid increase of dry weight value of *R*. *sativus*. This phenomenon is closely related with the fact that plants absorb nutrients and water through their roots [39].

Growth and development of a plant depends strongly on the concentration of mineral nutrients accessible in the soil. When the concentration of xenobiotics in a plant reaches the critical point, one of the mechanisms of defence against toxic compounds is to absorb the large amount of water in order to start the detoxication processes, including metabolic production of glutathione and superoxide dismutase. During exposure time, roots stop developing and growing, water absorption becomes more difficult, and the level of dry matter in a plant starts to increase. Such behaviour of dry matter during the exposure of plants to contaminants is commonly known.

Changes of aminophosphonates **1**–**6** on radish dry matter were in accordance with the no observed effect concentration (NOEC) and the lowest observed effect concentration (LOEC) values. A significant increase of percentage dry matter of thiophene derivatives **1** and **4** occurred at a concentration of 400 mg/kg s.d.w. The growth of radish dry weight was reflected in the LOEC and NOEC values for this plant, and it confirmed that the lowest observed effect concentration actually indicated the starting point of adverse effects of tested substances. In the case of substances **2**, **3**, **5**, and **6**, greater water absorption took place at a concentration of 800 mg/kg s.d.w.

### 3.3. Germination of Seeds

Percentage germination of *Avena sativa* seeds at the highest concentration (1000 mg/kg) of tested substances **1**–**6** ranged between 97 and 98% (Table 3).

In the case of *R*. *sativus*, at the same concentration (1000 mg/kg), germination ranged between 70 and 96%. Radish germination assessment showed that this plant is much more sensitive to exposition on tested compounds **1**–**6** than oat. This is in accordance with the case of inhibition of shoots, roots, and fresh weight, however with one exception—both derivatives with para-substitution at phenylamino moieties, i.e., diphenyl *N*-(4-methylphenyl)amino(2-thienyl)methylphosphonate (**3**) and diphenyl *N*-(4-methoxyphenyl)amino(2-thienyl)methylphosphonate (**6**), were found to impact germination of radish the most.

Analyzing the influence of compounds **1**–**6** on germination of both radish and oat plants, it is worth noting that this parameter is strongly dose-dependent, i.e., the higher the concentration of the tested substances in soil was, the stronger the adverse effect that was observed.

### 3.4. Values EC_50_, NOEC, LOEC

#### 3.4.1. EC_50_Values for Fresh Matter and Shoot Height

**Figure 2:**
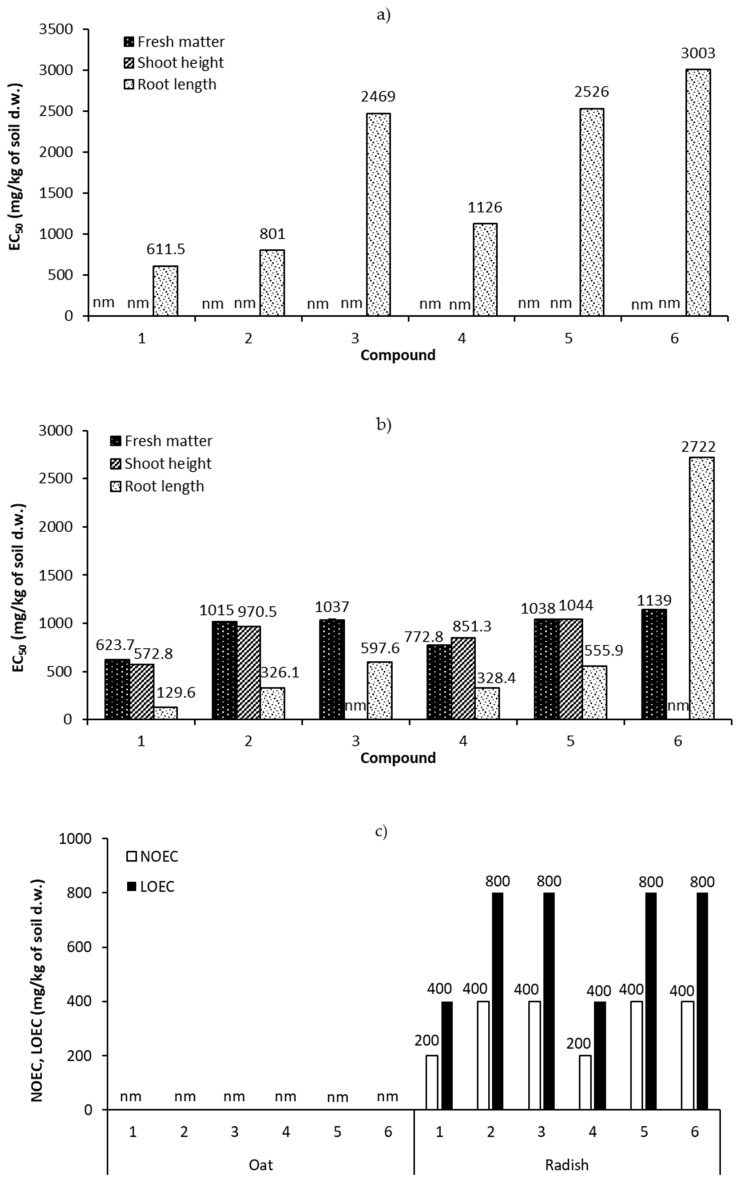
The EC_50_ values of growth inhibition of fresh matter, shoots, and roots of oat (*A. sativa*) (**a**) and common radish *(R. sativus*) (**b**), as well as NOEC and LOEC values related to fresh matter (**c**) of oat and radish following exposure to tested compounds **1**–**6** (nm = not measurable).

EC_50_ values (expressed in mg/kg of soil dry weight) of the test substances to shoot height and fresh matter of *Avena sativa* were too high to be measured precisely (Figure 2a).

Analysis of toxic effect for fresh matter of *Raphanus sativus* showed that from among *N*-methylphenyl-substituted aminophosphonates, the most poisonous is compound **1** (EC_50_ = 623.7 mg/kg s.d.w.), next is the *m*-methylphenyl derivative **2** (EC_50_ = 1015 mg/kg s.d.w.), and the lowest toxicity was noted for the *p*-methylphenyl isomer **3** (EC_50_ = 1037 mg/kg s.d.w.). The similar tendency was found for the aminophosphonates bearing metoxyphenyl moieties **4**–**6**; the *o*-methoxyphenyl derivative **4** was found to be the most toxic substance (EC_50_ = 772.8 mg/kg s.d.w.), next was the *m*-methoxyphenyl isomer **5** (EC_50_ = 1308 mg/kg s.d.w.), and the lowest toxicity was measured for the *p*-methoxyphenyl analog **6** (EC_50_ = 1139 mg/kg s.d.w.).

Variations of toxicity of compounds **1**–**3** against shoot length was similar to toxicity variations for aminophosphonates **4**–**6** (**1**: EC_50_ = 572.8 mg/kg s.d.w.; **2**: 970.5 mg/kg s.d.w.; **4**: EC_50_ = 851.3 mg/kg s.d.w and **5**: 1044 mg/kg s.d.w.). On the other hand, toxic effects for aminophosphonates with the para-substituted phenylamino moieties **3** and **6** were not measurable by GraphPad software, which means that these substances were not toxic at all for shoot height.

**Figure 3:**
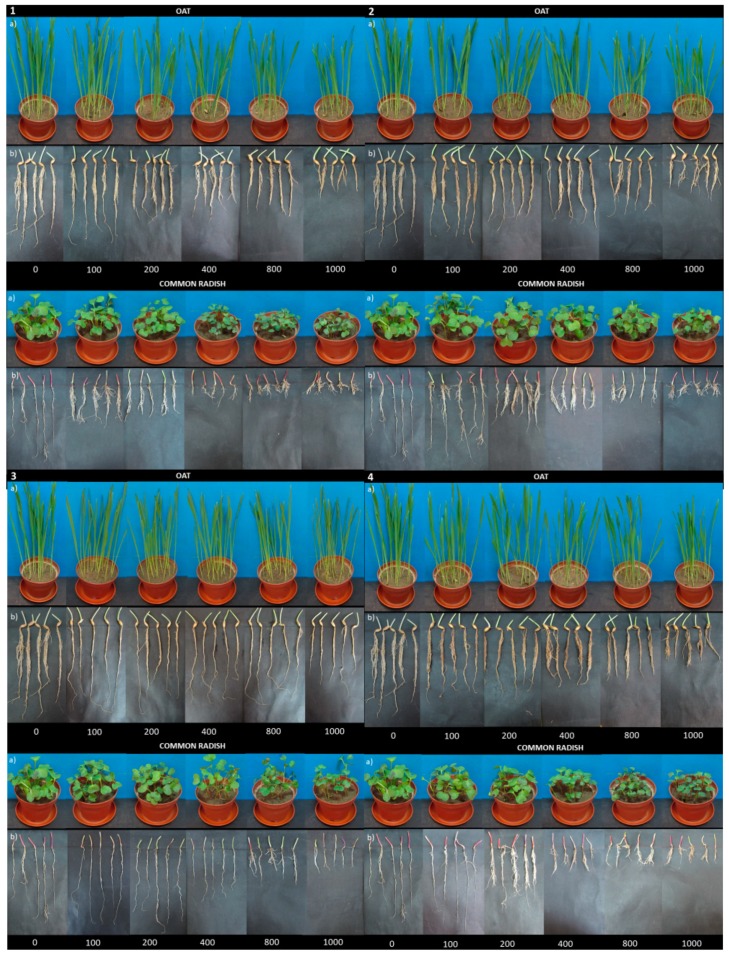

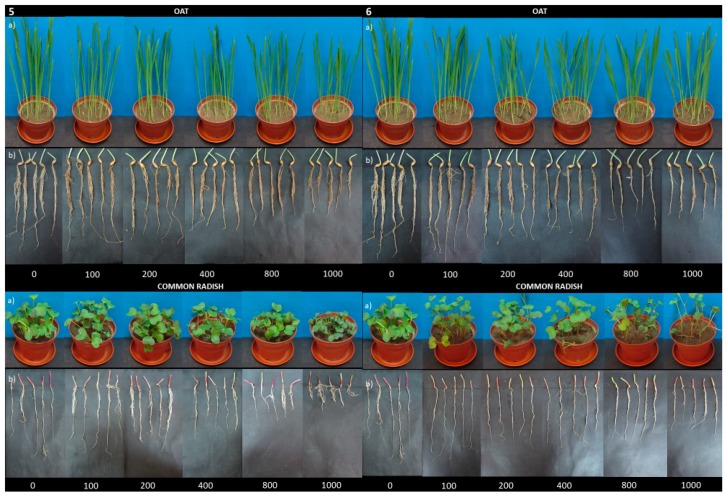
Digital photographs of oat and common radish: (**a**) shoots and (**b**) roots treated with compounds **1**–**6** (concentration in mg/kg of soil dry weight) on the fourteenth day of growth.

Digital photographs of emerged seedlings of oat and common radish after treatment with compounds **1**–**6** (Figure 3) were taken one day before determination of phytotoxicity to allow the visual evaluation of the impact of aminophosphonates **1**–**6**.

#### 3.4.2. EC_50_ Values for Root Length

The only factor for which EC_50_ values of compounds **1**–**6** were measurable for *Avena sativa*was the root length. Analyzing the impact of *N*-methylphenyl-substituted aminophosphonates **1**–**3**, it was found that the *o*-methylphenyl derivative (**1**) had the most inhibiting influence on the root of oat (EC_50_ = 611.5 mg/kg s.d.w.) when compared to the isomers meta **2** (EC_50_ = 801 mg/kg s.d.w) and para **3** (EC_50_ = 2469 mg/kg s.d.w.). Aminophosphonates with a metoxyphenyl moiety **4**–**6** were less toxic as compared to **1**–**3**, and their toxic impact was as follows : **4** > **5** > **6** (1126, 2526, and 3003 mg/kg s.d.w., respectively).

For *Raphanus sativus*, measuredEC_50_ values of examined compounds **1**–**6** indicated that the most harmful impact on roots of radish was observed for the*o*-methylphenyl derivative**1** (EC_50_ = 129.6 mg/kg s.d.w.). Undoubtably, both compounds **2** (EC_50_ = 326.1 mg/kg s.d.w.) **3** (EC_50_ = 597.6 mg/kg s.d.w.) were less toxic.

Among the aminophosphonates bearing a metoxyphenyl group, the following order of toxicity against radish root was found: **4** > **5**> **6** (**4**: EC_50_ = 328.4 mg/kg s.d.w;**5**: EC_50_ = 555.9 mg/kg s.d.w.; **6**: EC_50_ = 2722 mg/kg s.d.w.).

All these trends are visible in digital photographs showing roots of oat and common radish one day before determination of phytotoxicity (Figure 3).

Comparing EC_50_ values for roots, it was detected that radish roots were definitely more sensitive for tested compounds **1**–**6**.

#### 3.4.3. NOEC and LOEC Values

NOEC and LOEC values for *Avena sativa* were not determined because they exceeded 1000 mg/kg s.d.w.

Values of NOEC and LOEC of N-methylphenyl-substituted aminophosphonates **1**–**3** in action on *R. sativus* were as follows: 200 and 400 mg/kg s.d.w. for the ortho-substituted aminophosphonate **1**, and 400 and 800 mg/kg s.d.w for **2** and **3**. A similar tendency was observed for methoxyphenyl derivatives **4**–**6**, where NOEC and LOEC values for the ortho-substituted derivative (**4**) were: 200 and 400 mg/kg s.d.w., respectively, while for compounds **5** and **6** they were 400 and 800 mg/kg s.d.w., respectively.

### 3.5. Pigment Assay

#### 3.5.1. Changes of Total Chlorophyll Content

**Figure 4:**
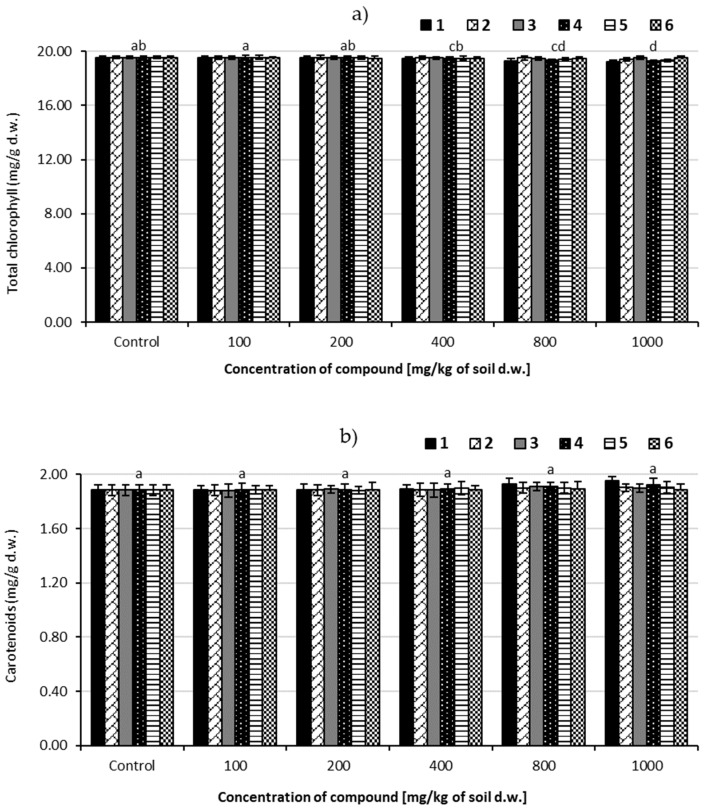

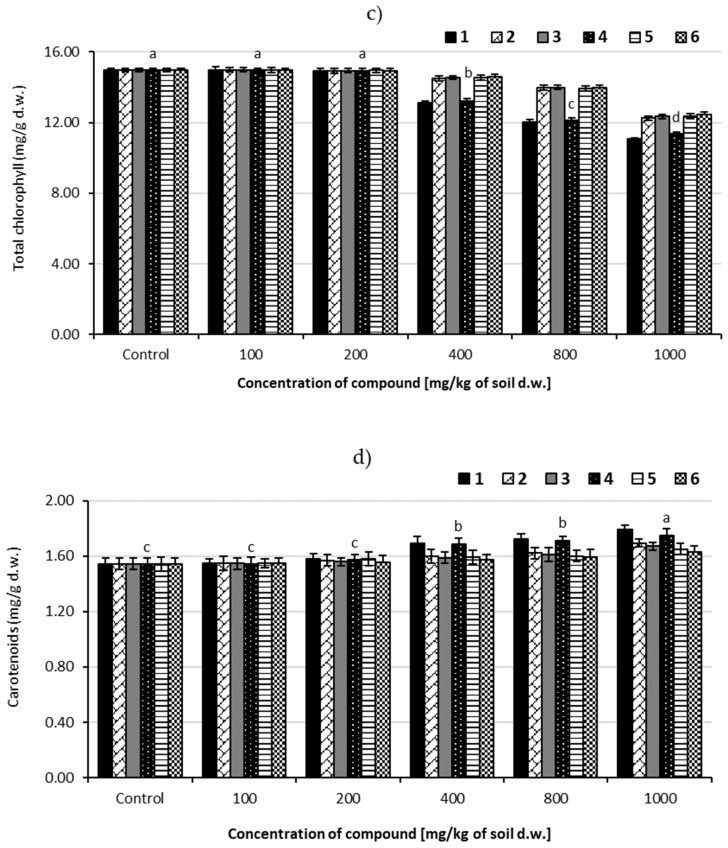
Values of compounds **1**–**6** for the content of total chlorophyll (**a**) and carotenoids (**b**) in oat seedlings and the content of total chlorophyll (**c**) and carotenoids (**d**) in radish leaves. Data are expressed as a mean ± SD of three replicates for each concentration. Values denoted by the same letters do not differ statistically at *p* < 0.05.

The level of total chlorophyll in *A. sativa* leaves treated with the tested compounds **1**–**6** was comparable to level of this pigment in control plants (Figure 4a).

The increasing concentration of the tested compounds **1**–**6** in the soil resulted in a reduction in total chlorophyll level in the green parts of radish plants (*R. sativus*) (Figure 4c).

Obtained results were in accordance with the results of determination of plant pigments under stress conditions [40,41,42]. Appearance of chlorosis symptoms on the leaves of the plant, such as yellowing and whitening of normally green plant tissue, indicates reduction in the amount of chlorophyll, causing disease (chlorosis) or nutrient deficiency. The chlorophyll content measured in the radish leaves depended on the compound used, with the strongest decrease of chlorophyll level in radish being observed after the exposure to ortho-substituted derivatives, namely diphenyl *N*-(2-methylphenyl)amino(2-thienyl)methylphosphonate (**1**) and diphenyl *N*-(2-methoxyphenyl)amino(2-thienyl)methylphosphonate (**4**). An important drop in the level of chlorophyll due to the impact of these two substances **1** and **4** was observed at the concentration of 400 mg/kg s.d.w (Figure 4c). For the rest of the compounds **2**, **3**, **5**, and **6**, the decrease in the level of chlorophyll was observed at 400 mg/kg s.d.w. also, but it was not so rapid.

#### 3.5.2. Changes of Carotenoids Content

The ratio of chlorophyll to carotenoids is a key factor accountable for the appropriate functionality of the photosynthesis system. Carotenoid pigments are critical to the survival of the plant, which is why their composition and amount strongly depends on the physiological and pathological conditions in which they live [43,44].

The levels of carotenoids in *A. sativa* seedlings treated with all aminophosphonates were comparable to the level of this pigment in control plants (Figure 4b).

However, in the case of *R. sativus* (Figure 4d), the increasing concentration of the tested compounds in soil caused a constant increase in the amount of carotenoids in green parts of the plant [45]. Carotenoids are necessary to protect chlorophyll against exposed biotic and abiotic factors, but the dependence of carotenoid level on the stress conditions is ambiguous [46,47,48,49]. One of the most important environmental factors affecting the level and metabolism of carotenoids is oxidative stress, which occurs when the absorbed energy exceeds the capacity of the photosynthetic plant device. A significant increase in the level of carotenoids was observed in the case of two *ortho*-substituted aminophosphonates **1** and **4**.

### 3.6. Weed Test

#### 3.6.1. Growth Inhibition of Shoot Height

The percentage growth inhibition (GI%) values for shoot height of three dicotyledonous persistent weeds, namely gallant soldier (*G. parviflora* Cav.), common sorrel (*R. acetosa* L.), and white goosefoot (*Ch. album* L.), exposed on aminophosphonates **1**–**6** are presented in Table 4.

Based on the obtained results, it was found that among aminophosphonates **1**–**3**, the *o*-methylphenyl-substituted aminophosphonate(**1**) was the most toxic to all weeds, and at the highest concentration (1000 mg/kg s.d.w.) it caused 55, 56, and 59% GI of shoot heights for gallant soldier, white goosefoot, and common sorrel, respectively. The toxic impact of compounds **2** and **3** was less harmfull for the mentioned weeds and the following growth inhibition percentages were calculated: 40, 42, and 51% for compound **2**, and 24, 39, and 51% for compound **3**.

Methoxyphenyl-substituted derivatives **4**–**6** were less toxic for all three tested weeds than methylphenyl-substituted compounds **1**–**3**. *N*-(2-methoxyphenyl)amino(2-thienyl)methylphosphonate (**4**) was found to be the most toxic of all methoxy-substituted compounds (**4**–**6**), giving GI% to be 42% for gallant soldier; 47% for white goosefoot and 51% for common sorrel. Aminophosphonate **5** showed GI% values of 34, 35, and 49%, respectfully, and substance **6** showed values of 18, 26, 42%, respectively.

**Figure 5:**
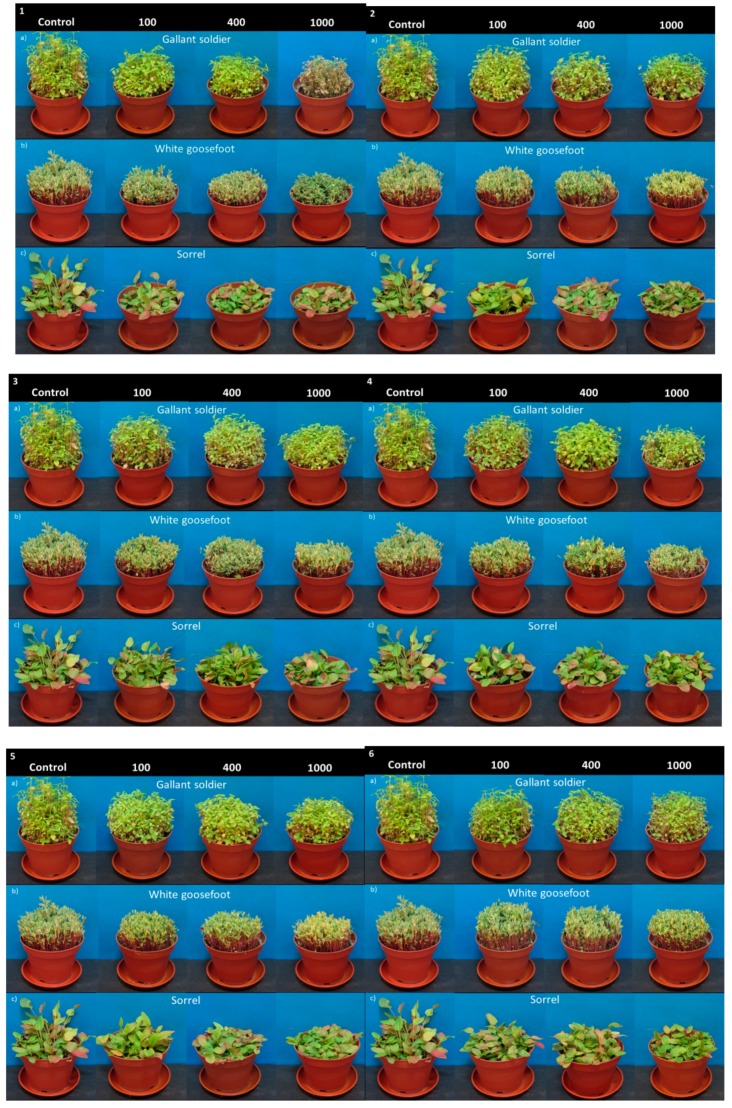
Digital photographs of (**a**) gallant soldier (*G. parviflora* Cav.), (**b**) white goosefoot (*Ch. album* L.), and (**c**) common sorrel (*R. acetosa* L.) treated with compounds **1**–**6** (concentration in mg/kg of soil dry weight) on the sixth week of growth.

All these trends are visible in digital photographs of weeds taken one day before determination of shoot inhibition (Figure 5).

Soil-applied herbicides penetrate the plant through the seed coat and for this reason, the difference in efficiency of compounds **1**–**6** may result from the different morphological structures of tested plants, which determine the degree of retention and penetration of the agent.

#### 3.6.2. EC_50_ for Shoot Height

**Figure 6:**
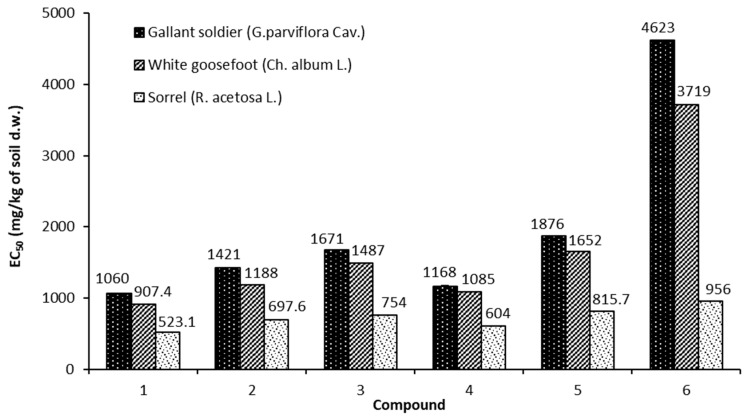
The EC_50_ values of growth inhibition of shoots of gallant soldier(*G. parviflora* Cav.), white goosefoot (*Ch. album* L.), and common sorrel (*R. acetosa* L.) plants following exposure to tested compounds **1**–**6**.

Changes of effective concentration values of EC_50_ (expressed in mg per kg of soil dry weight) are presented at Figure 6. Comparing *N*-methylphenyl-substituted aminophosphonates **1**–**3**, it was found that the *o*-methylphenyl-substituted aminophosphonate (**1**) demonstrated the highest inhibiting action on the growth of all weeds when compared to **2** and **3** (gallant solider EC_50_ = 1060 mg/kg; white goosefoot EC_50_ = 907.4 mg/kg; sorrel EC_50_ = 523.1 mg/kg). Diphenyl *N*-(3-methylphenyl)amino(2-thienyl)methylphosphonate (**2**) was found to be less harmful and the least toxic properties were found for the *N*-(4-methylphenyl) derivative**3**.

Aminophosphonates bearing metoxyphenyl moieties **4** and **5** showedless toxic character as compared to compounds **1**–**3**. Diphenyl *N*-(2-methoxyphenyl)amino(2-thienyl)methylphosphonate (**4**) was more toxic than the aminophosphonate **5**(gallant soldier EC_50_ = 1068 mg/kg; white goosefoot EC_50_ = 1085 mg/kg; sorrel EC_50_ = 604 mg/kg).The aminophosphonate**6** was found to be harmless for gallant soldier (EC_50_ = 4623 mg/kg) and white goosefoot (EC_50_ = 3719 mg/kg). On the whole, compounds **1** and **4** with a phenyl ring substituted in an *ortho* position, were more toxic than substances in the *meta* position (**2** and **5**). The lowest toxicity was found for *para*-substituted aminophosphonates (**3** and **6**).

#### 3.6.3. EWRC Rating Scale

**Figure 7:**
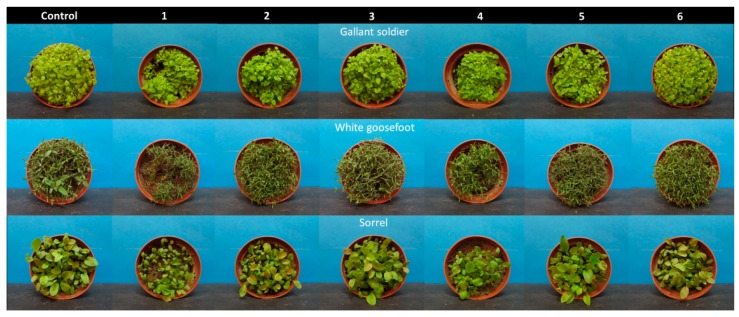
Digital photographs of gallant soldier (*G. parviflora Cav*.), white goosefoot (*Ch. album* L.), and common sorrel (*R. acetosa* L.) treated with compounds **1**–**6** (concentration in 1000 mg/kg of soil dry weight) on the third week of growth.

The visual impact of tested substances **1**–**6** on examined species of weeds is presented in Figure 7. Ratings were assigned based on scales from the European Weed Research Council (EWRC) and are presented in Table 5.

The highest efficiency for all weeds was observed for substance **1**, where according to the European Weed Research Council scale [33,34,35], a rank was 7 = bad (55.0 to 69.9%). In the case of aminophosphonates **2**–**5**, the EWRC scale rank was 8 = very bad (30 to 54.9%). For diphenyl N-(4-methoxyphenyl) -amino(2-thienyl)methylphosphonate (**6**), the EWRC rank was 9 = none (0.0 to 29.9%) for gallant soldier and white goosefoot, and for sorrel it was 8 = very bad (30 to 54.9%).

The aminophosphonates **1**–**6** were found to be completely ineffective as herbicides, being classified in the EWRC scale at 7-9 (EWRC: 7 = bad, 55.0 to 69.9%; 8 = very bad, 30 to 54.9%; 9 = none, 0.0 to 29.9%).

### 3.7. Microtox^®^ Toxicity Assay

**Figure 8:**
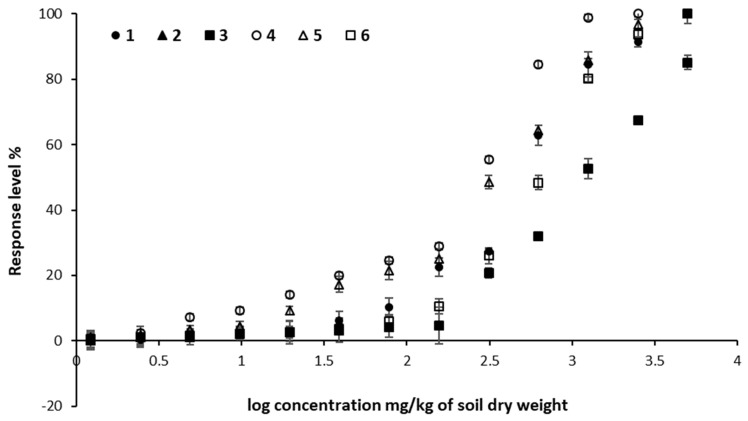
The EC_50_ graded dose-response curved for the six tested compounds **1**–**6**. Overlapped curves are plotted by Microtox^®^ Analyzer 500 software. Error bars represent standard deviation errors (SD; n = 3 determinations).

The potential usefulness of the tested aminophosphonates was verified by assessing their ecotoxicity. It is recommended to rate the ecotoxicity of each newly synthesized substance of potential biocidal application aptly based on the recommendation given by the ECHA, expressed in the Registration, Evaluation, and Authorisation of Chemicals (REACH) procedure. Toxicity assessment using bacteria *Aliivibrio fischeri* as test organisms was performed for all six tested compounds **1**–**6.** Values of EC_50_ calculated by Microtox^®^ Analyzer software are plotted in Figure 8 and shown in Table 6.

Analyzing these data, it is worth noting that aminophosphonates bearing an *ortho*-substituted phenyl ring were the most toxic for tested bacteria (**1** = EC_50_ = 410.3 mg/L;**4** = EC_50_ = 105.8 mg/L). The less toxic substances were those with a *meta*-substituted phenyl ring (**2** = EC_50_ = 422.5 mg/L; **5** = 232.9 mg/L), and the *para* substituted compounds were determined as the least toxic (**3** = EC_50_ = 1201 mg/L;**6** = EC_50_ = 538.9 mg/L).

According to Hernando et al. [50], the categories of toxicity against *Aliivibrio fischeri* are “*very toxic to aquatic organisms*” (when EC_50_ is below 1 mg/L), “*toxic*” (when EC_50_ is in the range of 1–10 mg/L), and “*harmful*” (when EC_50_ in the range of 10–100 mg/L). In such a way, aminophosphonates **1**–**3**, **5**, and **6** should be considered as harmless. Only substance **4** is harmful to *Aliivibrio fischeri* (EC_50_ = 105.8 mg/L). Aminophosphonates with the methoxy moieties **4**–**6** were found to be more toxic for *Aliivibrio fischeri* as opposed to their action on dicotyledonous tested plants (radish and weeds); as for these plants, methyl derivatives were more toxic. In order to facilitate the comparison, EC_50_ values for *Aliivibrio fischeri* were recalculated in mg/kg of soil dry weight.

### 3.8. Ostracod Test Kit

**Figure 9:**
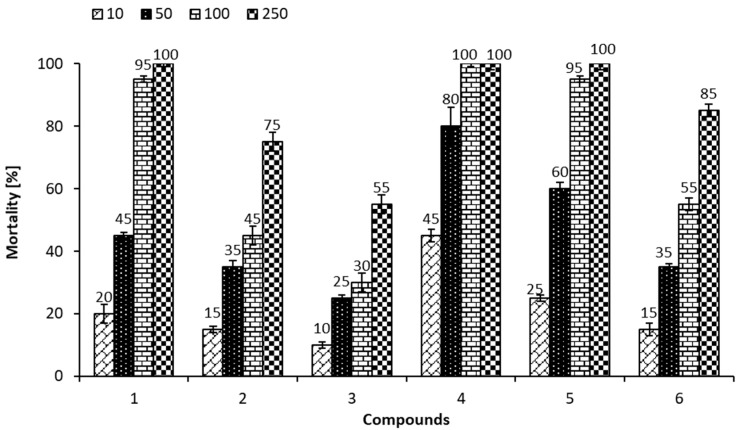
The mortality (%) of *Heterocypris incongruens* living in soil contaminated with compounds **1**–**6** at concentrations of 10, 50, 100, 250, and 500 mg/kg of soil dry weight (nm = not measurable). Error bars represent standard deviation error (SD; n = 6 determinations).

Mortality and percent growing inhibition assessment using *Heterocypris incongruens* showed that the effect of the test substances was dose (concentration)-dependent and structure-dependent (Table 7 and Figure 9).

As expected, higher concentrations of tested compounds **1**–**6** resulted in greater mortality for crustaceans (Figure 9). The effect of the tested aminophosphonic derivatives on crustaceans was determined based on the following substance concentrations: 10, 50, 100, and 250 mg/kg s.d.w. Mortality assessment using *Heterocypris incongruens* showed that the effect of the test substances was different depending on their concentration and structure.

Aminophosphonates **1**–**3**, **5**, and **6** caused over 10% mortality of ostracods at the lowest concentration (10 mg/kg s.d.w.), while diphenyl *N*-(2-methoxyphenyl)-amino(2-thienyl)- methylphosphonate caused death of 45% of the tested population. Also, the concentration of 50 mg/kg in the case of aminophosphonates **1**–**3** and **5** resulted in high mortality (above 25%) and very high mortality for substances **4** and **5** (60 and 80%, respectively). Almost total and total mortality of *Heterocypris incongruens* was observed at the concentration 100 mg/kg s.d.w. for thiophene-derived aminophosphonates **1** (95%), **4** (100%), and **5** (95%). These compounds, at their highest applied concentration (250 mg/kg of soil), were found to be the most toxic against examined crustaceans.

In addition to mortality, growth inhibition was the second criterion of the toxic effects indicated by the microbiotest Ostracodtoxkit F™. This criterion allows assessment of the sublethal toxicity of sediments. Inhibition of growth is determined by comparing the size of live spouses living in the studied settlement with the size of spouses living in the reference sludge at the end of the test. Determining the sublethal impact of toxic substances on sediments is justified only for sediments that do not cause high mortality among organisms (mortality less than 30%).

As expected, the higher the concentration of the test compound in the soil was, the higher the inhibition of the growth of organisms. At the concentration of 10 mg/kg s.d.w., GI% ranged from 4 to 12% for substances **1**–**3**, **5**, and **6**. In the case of the aminophosphonate **4**, growth inhibition of *H. incongruens* was not measured. This indicates the highest toxicity of this substance to ostracods, while the aminophosphonate **3** was the least toxic (GI% ranged from 4% for 10 mg/kg s.d.w. to 26% for 100 mg/kg s.d.w.).

The tendency of mortality changes of crustaceans *Heterocypris incongruens* was similar to changes in the toxicity of microorganisms *Aliivibrio fischeri*. *N*-methoxyphenyl aminophosphonates **4**–**6** were definitely more toxic than *N*-methylphenyl derivatives **1**–**3**. In both cases, compounds **1** and **4** were found to be the most toxic substances, followed by **4** and **5**, while the least toxic was **3** and **6**.

In our previous papers [23,24], we reported the phytotoxicological study of a series of dimethyl amino(2-thienyl)methylphosphonates, where their impact on common radish (*Raphanus sativus*) and oat (*Avena sativa*) was measured and characterized by a NOEC/LOEC factor and by the half maximal effective concentration (EC_50_) [23]. Consequently, aminophosphonic dimethyl esters of this series were investigated in respect to their impact on three common and persistent weeds [24]: gallant soldier (*Galinsoga parviflora* Cav.), white goosefoot (*Chenopodium album* L.), and common sorrel (*Rumex acetosa* L.); they were also tested in view of their ecotoxicological impact on bacteria *Aliivibrio fischeri*, as well as crustaceans *Heterocypris incongruens*. Results described in these papers [23,24] allowed selection of two compounds of herbicidal potency, and one of them, namely dimethyl *N*-(2-methylphenyl)amino(2-thienyl)methylphosphonate, demonstrated significantly low ecotoxicity against tested organisms.

Analysis of all collected data demonstrated that aminophosphonates **1**–**6** are totally out of interest from the point of view of their herbicidal potency. First of all, this was because their EC_50_ values of growth inhibition of the tested weed shoots following their exposure were significantly high (1060–4623 mg/kg s.d.w. for gallant soldier, 907.4–3719 mg/kg s.d.w. for white goosefoot, and 523.1–956 mg/kg s.d.w. for common sorrel) (Figure 7). The growth inhibition% (GI%) caused by compounds **2**–**6** at the highest applied concentration (1000 mg/kg s.d.w.) calculated for gallant soldier (*Galinsoga parviflora* Cav.) and white goosefoot (*Chenopodium album* L.) did not exceed 50%, while GI% calculated for common sorrel (*Rumex acetosa* L.) exceeded 51% only in the case of compounds **2**–**4** (Table 4, Figure 6). Exclusively, diphenyl *N*-(2-methylphenyl)amino(2-thienyl)methylphosphonate (**1**) caused GI% higher than 50% at the highest applied concentration (1000 mg/kg s.d.w.), with 55.3% for gallant soldier, 56.1% for white goosefoot, and 59.4% for common sorrel. The scores of tested compounds **2**–**6** according to the EWRC rating scale [33,34,35] were estimated to be 8: very bad (in the case of aminophosphonate **6**, and even a score of 9 for gallant soldier and white goosefoot), and diphenyl *N*-(2-methylphenyl)amino- (2-thienyl)methylphosphonate (**1**) was ranked a score of 7: bad [33,34,35] (Table 5, Figure 8).

Compounds **2**–**5** were found to be harmful for radish (*Raphanus sativus*), especially badly effecting its roots, as EC_50_ ranged between 326.1 mg/kg s.d.w. (for **2**) and 555.9 mg/kg s.d.w. (for **5**). Diphenyl *N*-(4-methoxyphenyl)amino(2-thienyl)methylphosphonate (**6**) was found to be harmless for radish roots but more harmful for its fresh matter (EC_50_ = 1139 mg/kg s.d.w.) and tests for *N*-(2-methylphenyl)amino- (2-thienyl)methylphosphonate (**1**) showed its general harmful action on this plant (Figure 2b). Aminophosphonates **1**–**2** and **4** showed also their harmful character for oat (*Avena sativa*) roots (EC_50_ = 611.5, 801 and 1126 mg/kg s.d.w., respectively) (Figure 2a).

Ecotoxicological tests performed on aminophosphonates **1**–**6** did not show their toxic or important harmful character against tested organisms but one cannot state that they are harmless for these organisms. The only harmless compound for bacteria *Aliivibrio fischeri* was diphenyl *N*-(4-methylphenyl)amino-(2-thienyl)methylphosphonate (**3**), which gave and EC_50_ value of 1201 mg/L. Aminophosphonates **1**–**2** and **5**–**6** were slightly harmful for bacteria and diphenyl *N*-(2-methoxyphenyl)amino(2-thienyl)methylphosphonate (**4**) was found to be the most toxic, with an EC_50_ value of 105.8 mg/L (Table 6).

Aminophosphonates **1**–**6** were found to be moderately harmful for crustaceans *Heterocypris incongruens*. At the lowest applied concentration (10 mg/kg s.d.w.), compounds **1**–**3** and **5**–**6** gave ostracod mortality in the range of 10–25%, but at 50 mg/kg s.d.w., diphenyl *N*-(4-methylphenyl)amino-(2-thienyl)methylphosphonate (**3**) was the only compound giving mortality below 30% (25%). All the rest, i.e., aminophosphonates **1**–**2** and **5**–**6**, were lethal from 35 up to 60%. Diphenyl *N*-(2-methoxyphenyl)amino(2-thienyl)methylphosphonate (**4**) was found to be the most toxic for ostracods, giving 45% mortality at the lowest applied concentration (10 mg/kg s.d.w.) and reaching 80% at 50 mg/kg s.d.w.

All of the above results demonstrate clearly that none of diphenyl amino(2-thienyl)methyl- phosphonates **1**–**6** have any herbicidal potential—they are practically not phytotoxic against tested weeds, but they are, however, slightly phytotoxic against important food crops, namely oat and radish, moreover, the investigated compounds **1–6** are slightly but evidently ecotoxic against marine bacteria *Aliivibrio fischeri* and freshwater crustaceans *Heterocypris incongruens*.

As was mentioned in the introductory part, compounds bearing a thiophene moiety were found to have various biological properties. Thus, important microbiocidal, herbicidal, or cytotoxic properties of the studied thiophene-derived aminophosphonic systems **1**–**6** cannot be excluded. For these reasons, the large- or medium-scale preparation of these compounds for potential agrochemical or pharmacological application should be taken into consideration and the environmental protection aspect should also be excogitated. Our results call attention to the necessity of further toxicological investigation of potential products and wastes bearing thiophene-derived aminophosphonic systems.

## 4. Conclusions

A series of novel diphenyl *N*-arylaminophosphonates **1**–**6** bearing a 2-thienyl moiety was prepared and synthesized compounds were evaluated with respectto their phytotoxicological properties. Tested aminophosphonate derivatives exhibited more adverse effects on dicotyledonous than monocotyledonous plants, especially on their underground parts. Obtained results showed clearly that aminophosphonates **1**–**6** do not have any potential as soil-applied herbicides due to their very weak inhibitory effect on investigated plants.

All investigated compounds **1**–**6** were also tested with respect to their ecotoxicological impact on *Heterocypris incongruens* (OSTRACODTOXKIT^TM^) and *Aliivibrio fischeri* (Microtox^®^ test). The results showed distinctly that onlyaminophosphonate **3** is practically not toxic for bacteria *Aliivibrio fischeri*, while the rest (**1**–**2** and **3**–**6**) showed slightly harmful effects on these bacteria. All tested aminophosphonates are moderately toxic for crustaceans *Heterocypris incongruens*.

This paper is intended to call attention to the necessity for careful handling of any potential wastes containing investigated compounds **1**–**6** because of their possible hazardous impact on the environment.

## Figures and Tables

**Table 1:** Effect of compounds **1**–**6** on the fresh matter, shoot height, and root length of oat seedling plants compared to control (mean ± SD; n = 15).

Compound Concentration (mg/kg s.d.w.)	Inhibition Biomarkers (%)					
Fresh matter						
	1	2	3	4	5	6
100	−0.4 ± 0.2	0.4 ± 0.2	−0.4 ± 0.4	0.2 ± 0.1	0.2 ± 0.0	1.6 ± 0.4
200	2.9 ± 0.1	−0.5 ± 0.1	0.6 ± 0.4	0.5 ± 0.1	2.3 ± 0.2	2.9 ± 0.2
400	5.0 ± 0.1	0.6 ± 0.1	1.2 ± 0.1	2.2 ± 0.2	1.5 ± 0.2	3.4 ± 0.2
800	5.7 ± 0.4	3.5 ± 0.2	4.1 ± 0.1	3.7 ± 0.1	2.6 ± 0.1	1.8 ± 0.1
1000	8.0 ± 0.1	4.7 ± 0.3	1.6 ± 0.3	6.1 ± 0.1	3.7 ± 0.1	1.5 ± 0.1
Shoot height						
	1	2	3	4	5	6
100	0.4 ± 0.6	0.4 ± 0.5	−0.4 ± 0.6	0.5 ± 1.0	0.5 ± 0.2	1.3 ± 0.6
200	−0.5 ± 1.2	−0.2 ± 0.7	0.5 ± 0.8	0.9 ± 0.6	2.9 ± 0.3	2.9 ± 0.3
400	1.6 ± 1.0	1.8 ± 0.9	1.8 ± 0.8	3.8 ± 0.5	1.8 ± 0.4	3.1 ± 0.5
800	3.1 ± 0.6	3.3 ± 1.4	3.8 ± 0.8	4.6 ± 0.6	2.4 ± 0.2	1.3 ± 0.2
1000	5.8 ± 1.1	4.4 ± 0.5	2.0 ± 1.0	6.0 ± 1.0	5.5 ± 1.0	0.5 ± 0.5
Root length						
	1	2	3	4	5	6
100	3.5 ± 0.1	2.4 ± 0.2	4.0 ± 0.5	6.7 ± 0.8	5.9 ± 0.8	0.5 ± 0.7
200	17.4 ± 0.6	21.1 ± 0.1	7.8 ± 0.6	9.6 ± 0.3	5.3 ± 0.7	4.3 ± 0.3
400	43.9 ± 0.7	29.4 ± 0.3	8.8 ± 0.7	18.2 ± 0.2	9.6 ± 0.3	9.9 ± 0.6
800	51.1 ± 0.7	42.0 ± 0.8	11.8 ± 0.6	28.1 ± 0.4	16.8 ± 0.7	18.4 ± 0.1
1000	68.7 ± 0.4	63.4 ± 4.8	30.5 ± 0.6	53.7 ± 0.3	28.6 ± 0.2	21.7 ± 0.7

**Table 2:** Effect of compounds **1**–**6** on the fresh matter, shoot height, and root length of radish seedling plants compared to control (mean ± SD; n = 15).

Compound Concentration (mg/kg s.d.w.)	Inhibition Biomarkers (%)					
Fresh matter						
	1	2	3	4	5	6
100	3.9 ± 0.2	−0.8 ± 0.1	0.1 ± 0.1	2.9 ± 0.2	4.4 ± 0.2	−1.5 ± 0.2
200	10.3 ± 0.1	0.9 ± 0.1	1.3 ± 0.2	5.6 ± 0.1	5.8 ± 0.1	1.1 ± 0.1
400	48.2 ± 0.1	8.5 ± 0.1	9.3 ± 0.1	41.5 ± 0.1	5.1 ± 0.2	8.0 ± 0.2
800	53.8 ± 0.0	26.2 ± 0.0	21.8 ± 0.1	49.0 ± 0.1	14.3 ± 0.2	19.8 ± 0.2
1000	63.4 ± 0.1	48.9 ± 0.0	47.4 ± 0.1	55.6 ± 0.0	43.8 ± 0.1	41.0 ± 0.1
Shoot height						
	1	2	3	4	5	6
100	3.6 ± 0.7	−2.8 ± 0.2	0.6 ± 0.5	3.5 ± 0.2	3.4 ± 0.7	−0.9 ± 0.4
200	12.3 ± 0.2	0.3 ± 0.5	0.9 ± 0.5	5.4 ± 0.6	5.4 ± 0.6	−1.3 ± 0.3
400	49.8 ± 0.5	9.5 ± 0.7	2.2 ± 0.9	38.2 ± 0.4	5.7 ± 0.6	−1.9 ± 0.2
800	56.5 ± 0.4	21.5 ± 0.2	−0.6 ± 0.3	46.7 ± 0.4	13.2 ± 0.3	−3.2 ± 0.7
1000	66.9 ± 0.4	55.2 ± 0.3	−1.6 ± 0.3	52.4 ± 0.4	42.6 ± 0.5	−4.1 ± 0.2
Root length						
	1	2	3	4	5	6
100	48.6 ± 0.2	0.8 ± 0.6	−0.6 ± 0.2	2.0 ± 0.3	2.2 ± 0.6	−0.8 ± 0.2
200	53.7 ± 0.4	43.8 ± 0.3	−1.7 ± 0.5	35.7 ± 0.6	15.7 ± 0.6	4.5 ± 0.7
400	72.8 ± 0.7	64.9 ± 0.6	27.0 ± 0.7	68.8 ± 0.4	30.6 ± 0.4	9.5 ± 0.7
800	86.2 ± 0.1	71.1 ± 0.2	69.1 ± 0.4	73.9 ± 1.0	70.2 ± 0.3	16.4 ± 0.5
1000	89.0 ± 0.8	76.1 ± 0.5	76.1 ± 0.6	79.2 ± 1.0	74.0 ± 0.5	22.9 ± 0.6

**Table 3:** Average changes (mean of three replicates) of germination of oat (*A*. *sativa*) and radish (*R*. *sativus*) treated with compounds **1**–**6**. Least significant differences for samples (LSD_S_) and concentrations (LSD_C_) are given for each tested parameter. Germination% refers to number of emerged plants expressed as a percent of control plants.

Sample Concentration (mg/kg of s.d.w.)	Number of Emerged Seedlings						Germination%					
	Oat											
	1	2	3	4	5	6	1	2	3	4	5	6
Control	20	20	20	20	20	20	100	100	100	100	100	100
100	20	20	20	20	20	20	100	100	100	100	100	100
200	19	20	20	20	20	20	98	100	100	100	100	100
400	19	20	19	19	20	20	98	100	98	98	100	100
800	19	19	19	19	20	20	97	98	98	98	100	100
1000	19	19	19	19	19	19	97	97	97	98	97	98
	LSD_S_ = 1 LSD_C_ = 1											
	Radish											
	1	2	3	4	5	6	1	2	3	4	5	6
Control	19	19	19	19	19	19	100	100	100	100	100	100
100	19	19	19	19	19	19	100	100	100	100	100	100
200	19	19	19	19	19	19	100	98	100	100	98	100
400	19	18	18	19	19	18	98	96	96	98	98	96
800	18	18	17	18	19	16	96	96	89	96	98	84
1000	17	18	14	17	18	13	89	95	74	89	96	70
	LSD_S_ = 1 LSD_C_ = 2											

**Table 4:** Effect of **1**–**6** on shoot height of gallant soldier (*G. parviflora* Cav.), white goosefoot (*Ch. album* L.), and common sorrel (*R. acetosa* L.)compared to control (mean ± SD; n = 15).

Compound Concentration (mg/kg s.d.w.)	Inhibition (%)					
Gallant soldier (*Galinsoga parviflora* Cav.)						
	**1**	**2**	**3**	**4**	**5**	**6**
100	8.9 ± 0.6	8.7 ± 0.6	8.1 ± 0.5	7.9 ± 0.3	7.6 ± 0.3	7.7 ± 0.8
400	22.1 ± 1.1	13.0 ± 0.8	9.7 ± 0.3	8.2 ± 0.5	8.9 ± 0.5	6.9 ± 0.9
1000	55.3 ± 0.6	40.0 ± 1.1	34.1 ± 0.45	42.1 ± 0.4	34.1 ± 1.2	18.3 ± 0.6
White goosefoot (*Chenopodium album* L.)						
	**1**	**2**	**3**	**4**	**5**	**6**
100	23.3 ± 0.5	21.0 ± 1.1	18.1 ± 0.4	21.9 ± 0.3	7.6 ± 0.3	3.9 ± 0.8
400	41.7 ± 0.5	33.3 ± 0.7	26.3 ± 1.5	35.1 ± 0.6	27.5 ± 0.8	10.6 ± 0.7
1000	56.1 ± 1.2	42.2 ± 0.8	39.3 ± 0.4	47.0 ± 0.6	35.1 ± 0.7	26.3 ± 0.2
Sorrel (*Rumex acetosa* L.)						
	**1**	**2**	**3**	**4**	**5**	**6**
100	50.9 ± 0.3	47.3 ± 0.7	26.4 ± 0.4	28.7 ± 0.3	27.4 ± 0.3	24.4 ± 0.6
400	53.2 ± 1.1	49.4 ± 0.4	31.4 ± 0.8	33.7 ± 1.3	32.5 ± 0.6	29.6 ± 0.9
1000	59.4 ± 0.5	51.2 ± 1.1	51.6 ± 0.4	51.5 ± 0.4	49.0 ± 0.2	42.4 ± 0.5

**Table 5:** Poisoning of the plants on the third week of growth after application of tested compounds **1**–**6.**

Weeds	Compounds						
	Control	1	2	3	4	5	6
Gallant soldier (*G. parviflora* Cav.)	−	7	8	8	8	8	9
White goosefoot (*Ch. album* L.)	−	7	8	8	8	8	9
Common sorrel (*R. acetosa* L.)	−	7	8	8	8	8	8

Note: 1 = total plant death (100%); 2 = excellent (98.0 to 99.9%); 3 = very good (95.0 to 97.9%); 4 = good to acceptable (90 to 94.9%); 5 = moderate (82.0 to 89.9%); 6 = weak (70.0 to 81.9%); 7 = bad (55.0 to 69.9%); 8 = very bad (30 to 54.9%); and 9 = none (0.0 to 29.9%).

**Table 6:** Microtox^®^ EC_50_ values (mg/kg of soil dry weight) of exposure of the luminescent marine bacteria *Aliivibriofischeri* to tested substances **1**–**6** with respective 95% confidence limits (in brackets) obtained for the fit of the data.

Compound	EC_50_ (Lower Limit; Upper Limit (mg/L))	EC_50_ (Lower Limit; Upper Limit (mg/kg s.d.w.))	Coefficient of Determination (R^2^)
**1**	410.3 (356.1;472.7)	541.6 (470;623.9)	0.9746
**2**	422.5 (365.9;487.9)	557.7 (219.6;644)	0.9750
**3**	1201 (1065;1354)	1585.3 (1405.8;1787.3)	0.0979
**4**	105.8 (69.99;159.9)	139.6 (92.4;211.1)	0.8167
**5**	232.9 (182.7;296.9)	307.4 (241.2;391.9)	0.9290
**6**	538.9 (483.7;600.6)	710.9 (638.5;792.7)	0.9836

**Table 7:** Growth inhibition (%) of *Heterocypris incongruens* living in soil contaminated with compounds **1**–**6** at concentrations of 10, 50, 100, and 250 mg/kg of soil dry weight. SD means standard deviation (SD; n = 6 determinations). NM = not measured due to mortality rate higher than 30%.

Compound Concentration (mg/kg s.d.w.)	Growth Inhibition (%)					
	1	2	3	4	5	6
**10**	11 ± 2	9 ± 1	4 ± 1	NM	12 ± 1	6 ± 1
**50**	NM	NM	14 ± 1	NM	NM	NM
**100**	NM	NM	26 ± 2	NM	NM	NM
**250**	NM	NM	NM	NM	NM	NM

## Supplementary Materials

The following are available online at https://www.mdpi.com/1996-1944/12/12/2018/s1. Supplementary Material Part 1 with Figures S1–S6: scans of NMR spectra of aminophosphonates **1**–**6**, respectively. Supplementary Material Part 2, where all the detailed experimental procedures are given.
