# Supplementary material for: The Effect of New Thiophene-Derived Diphenyl Aminophosphonates on Growth of Terrestrial Plants
Source: Materials (Basel). 2019 Jun 24;12(12):2018. doi: 10.3390/ma12122018 (PMC6630915; doi:10.3390/ma12122018)
Supplement: Supplementary file 1 [file materials-12-02018-s001.zip › materials-519639 supplemenatry final check/Materials 519639 Supplementary 1.pdf]

The Effect of New Thiophene-Derived Diphenyl Aminophosphonates on Growth of Terrestrial Plants

Diana Rogacz [1], Jarosław Lewkowski [2,*], Marta Siedlarek[2], Rafał Karpowicz[2], Anna Kowalczyk[2] and Piotr Rychter [1,*]

[1]*Faculty of Mathematics and Natural Science, Jan Długosz University in Częstochowa, 42-200 Częstochowa, 13/15 Armii Krajowej Av., Poland;*
diana.rogacz@gmail.com (D.R.)

[2]*Department of Organic Chemistry, Faculty of Chemistry, University of Łódź, Tamka 12, 91-403 Łódź, Poland;*
mz.morawska@gmail.com (M.S.); rafalkarpowicz@gmail.com (R.K.)

*Correspondence: jaroslaw.lewkowski@chemia.uni.lodz.pl (J.L.); p.rychter@ajd.czest.pl (P.R.); Tel.: +48-42-635-5751 (J.L.); +48-34-361-5154 (P.R.)

Supplementary Material part 1

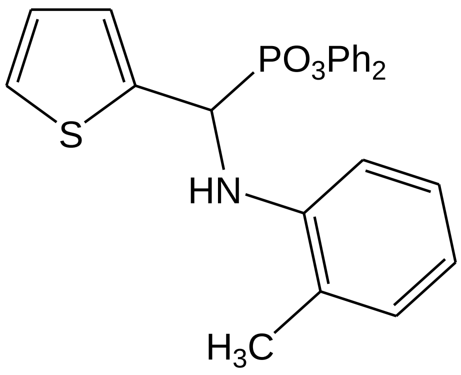

Fig. S1. ^{1}H NMR, ^{13}C NMR, ^{31}P NMR spectra of diphenyl N-(2-methylphenyl)amino(2-thienyl)methylphosphonate **(1)**

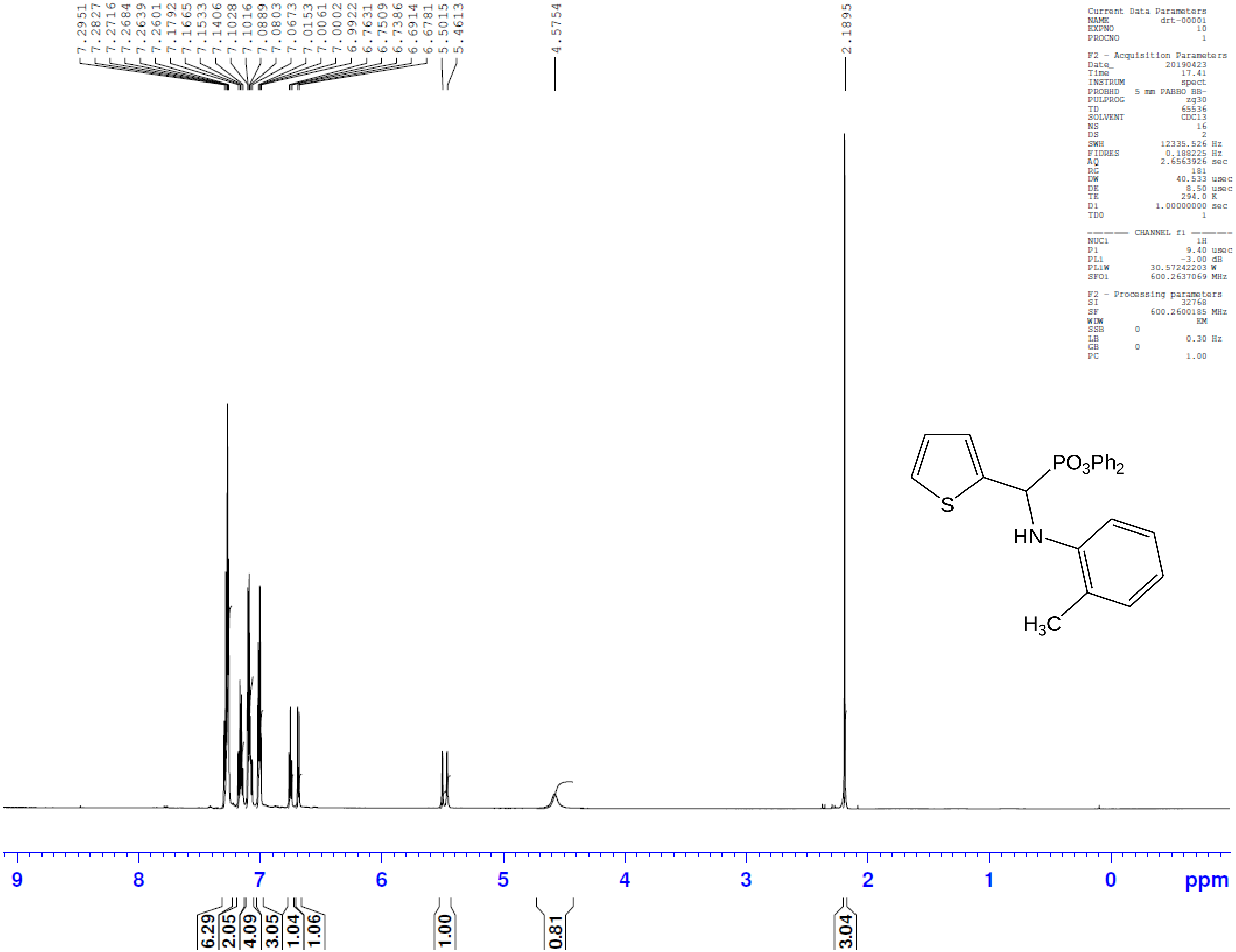
7.2951
7.2827
7.2716
7.2684
7.2639
7.2601
7.1792
7.1665
7.1533
7.1406
7.1028
7.1016
7.0889
7.0803
7.0673
7.0153
7.0061
7.0002
6.9922
6.7631
6.7509
6.7386
6.6914
6.6781
5.5015
5.4613
4.5754
2.1895
Current Data Parameters
NAME drt-00001
EXPNO 10
PROCNO 1
F2 - Acquisition Parameters
Date_ 20190423
Time 17.41
INSTRUM spect
PROBHD 5 mm PABBO BB-
PULPROG zg30
TD 65536
SOLVENT CDCl3
NS 16
DS 2
SWH 12335.526 Hz
FIDRES 0.188225 Hz
AQ 2.6563926 sec
RG 181
DW 40.533 usec
DE 8.50 usec
TE 294.0 K
D1 1.00000000 sec
TD0 1
CHANNEL f1
NUC1 1H
P1 9.40 usec
PL1 -3.00 dB
PL1W 30.57242203 W
SFO1 600.2637069 MHz
F2 - Processing parameters
SI 32768
SF 600.2600185 MHz
WDW EM
SSB 0
LB 0.30 Hz
GB 0
PC 1.00
PO3Ph2
S
HN
H3C
9
8
7
6
5
4
3
2
1
0
ppm
6.29
2.05
4.09
3.05
1.04
1.06
1.00
0.81
3.04

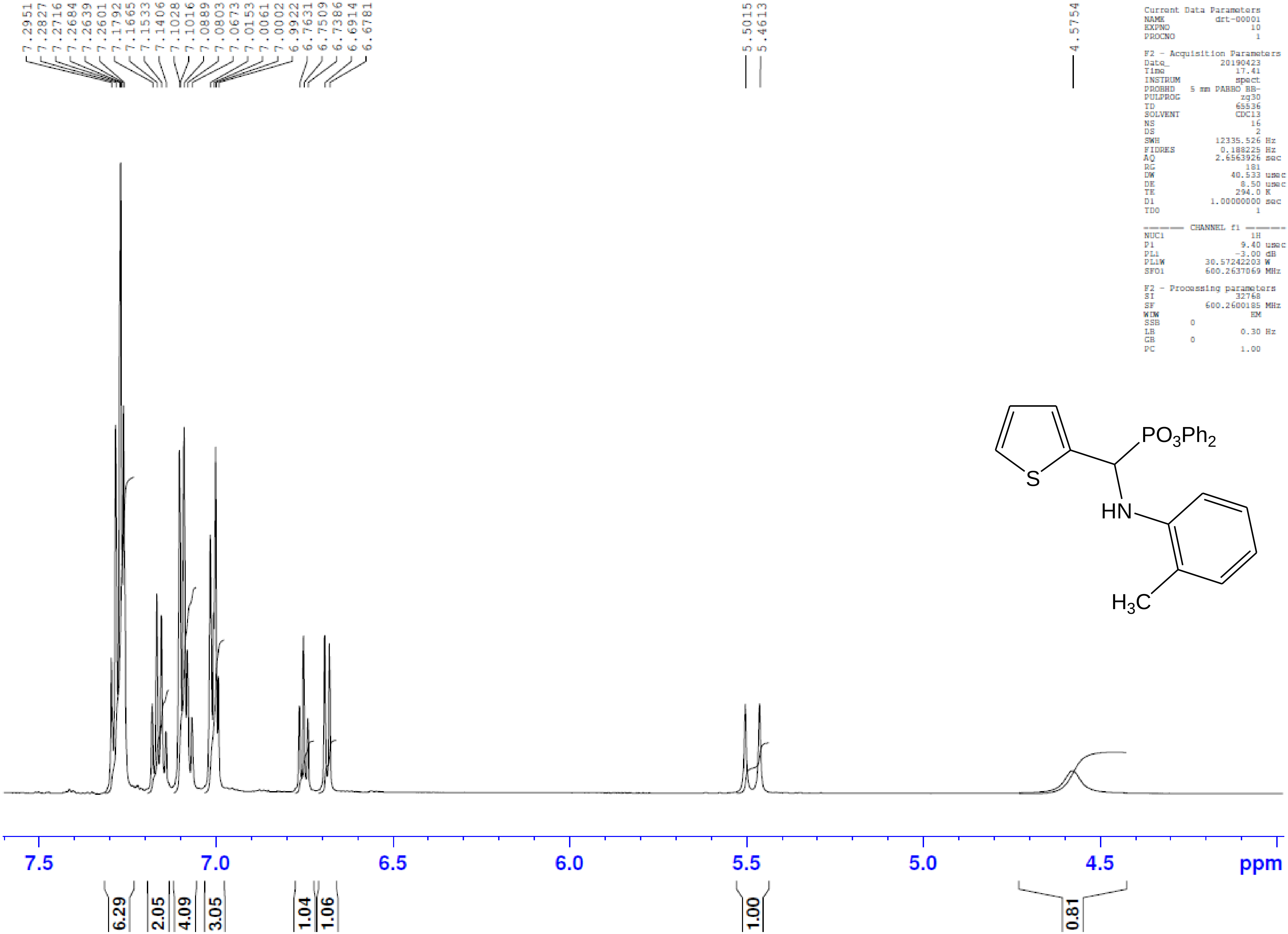
7.2951
7.2827
7.2716
7.2684
7.2639
7.2601
7.1792
7.1665
7.1533
7.1406
7.1028
7.1016
7.0889
7.0803
7.0673
7.0153
7.0061
7.0002
6.9922
6.7631
6.7509
6.7386
6.6914
6.6781
5.5015
5.4613
4.5754
Current Data Parameters
NAME drt-00001
EXPNO 10
PROCNO 1
F2 - Acquisition Parameters
Date_ 20190423
Time 17.41
INSTRUM spect
PROBHD 5 mm PABBO BB-
PULPROG zg30
TD 65536
SOLVENT CDCl3
NS 16
DS 2
SWH 12335.526 Hz
FIDRES 0.188225 Hz
AQ 2.6563926 sec
RG 181
DW 40.533 usec
DE 8.50 usec
TE 294.0 K
D1 1.00000000 sec
TD0 1
======== CHANNEL f1 ========
NUC1 1H
P1 9.40 usec
PL1 -3.00 dB
PL1W 30.57242203 W
SFO1 600.2637069 MHz
F2 - Processing parameters
SI 32768
SF 600.2600185 MHz
WDW EM
SSB 0
LB 0.30 Hz
GB 0
PC 1.00
PO3Ph2
S
HN
H3C
7.5
7.0
6.5
6.0
5.5
5.0
4.5
ppm
6.29
2.05
4.09
3.05
1.04
1.06
1.00
0.81

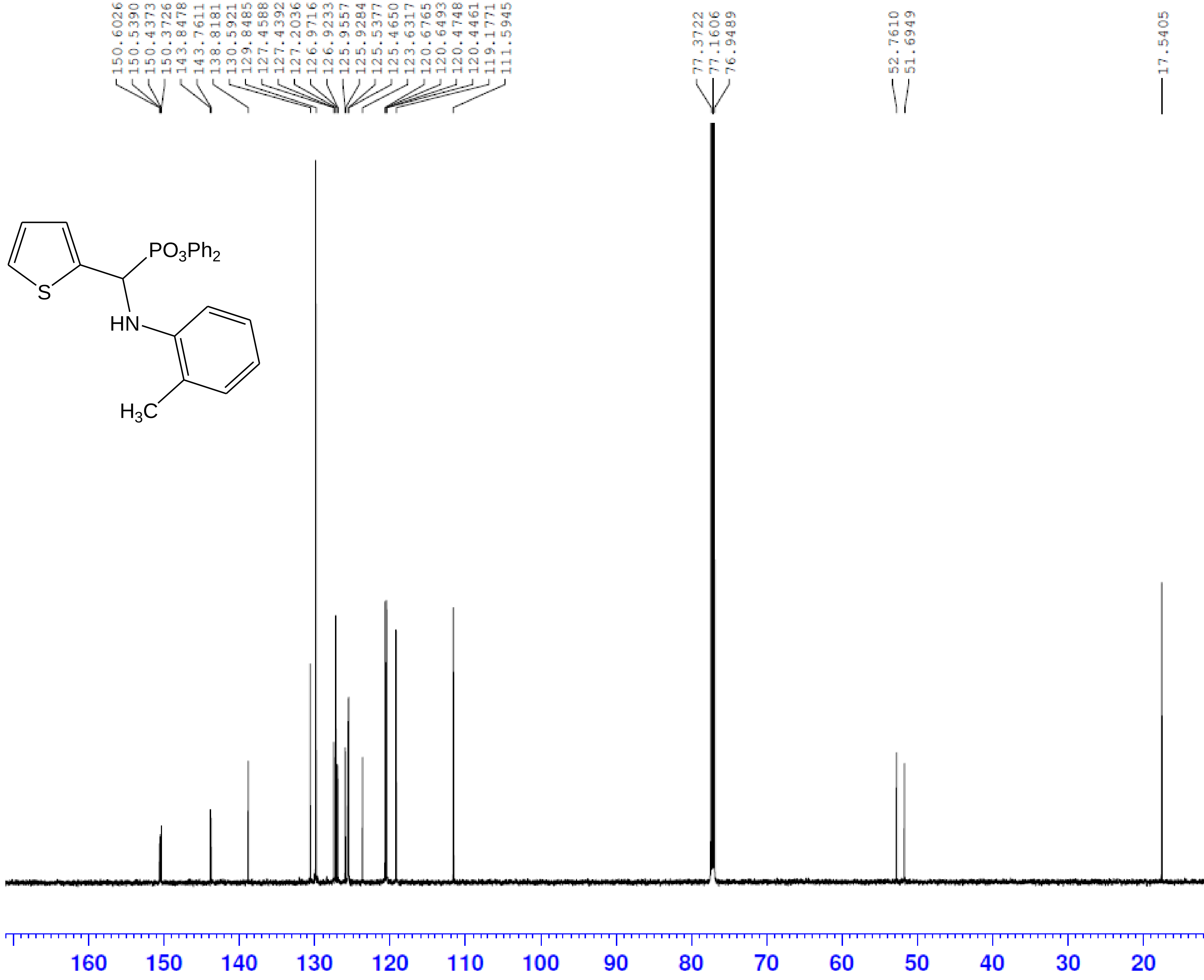


```
Current Data Parameters
NAME             drt-00001
EXPNO                   11
PROCNO                   1

F2 - Acquisition Parameters
Date_             20190423
Time                 18.34
INSTRUM              spect
PROBHD   5 mm PABBO BB-
PULPROG            zgpg30
TD                   65536
SOLVENT              CDCl3
NS                    1024
DS                       4
SWH              36057.691 Hz
FIDRES            0.550197 Hz
AQ               0.9087659 sec
RG                    2050
DW                  13.867 usec
DE                    8.50 usec
TE                   294.0 K
D1              2.00000000 sec
D11             0.03000000 sec
TD0                      1

======== CHANNEL f1 ========
NUC1                   13C
P1                   10.50 usec
PL1        0 dB
PL1W           91.93504333 W
SFO1           150.9505906 MHz

======== CHANNEL f2 ========
CPDPRG[2           waltz16
NUC2                    1H
PCPD2                90.00 usec
PL2                  -3.00 dB
PL12                 16.26 dB
PL13                 18.00 dB
PL2W           30.57242203 W
PL12W           0.36251819 W
PL13W           0.24284537 W
SFO2           600.2624010 MHz

F2 - Processing parameters
SI                   32768
SF             150.9354840 MHz
WDW                     EM
SSB        0
LB                    1.00 Hz
GB         0
PC                    1.40
```

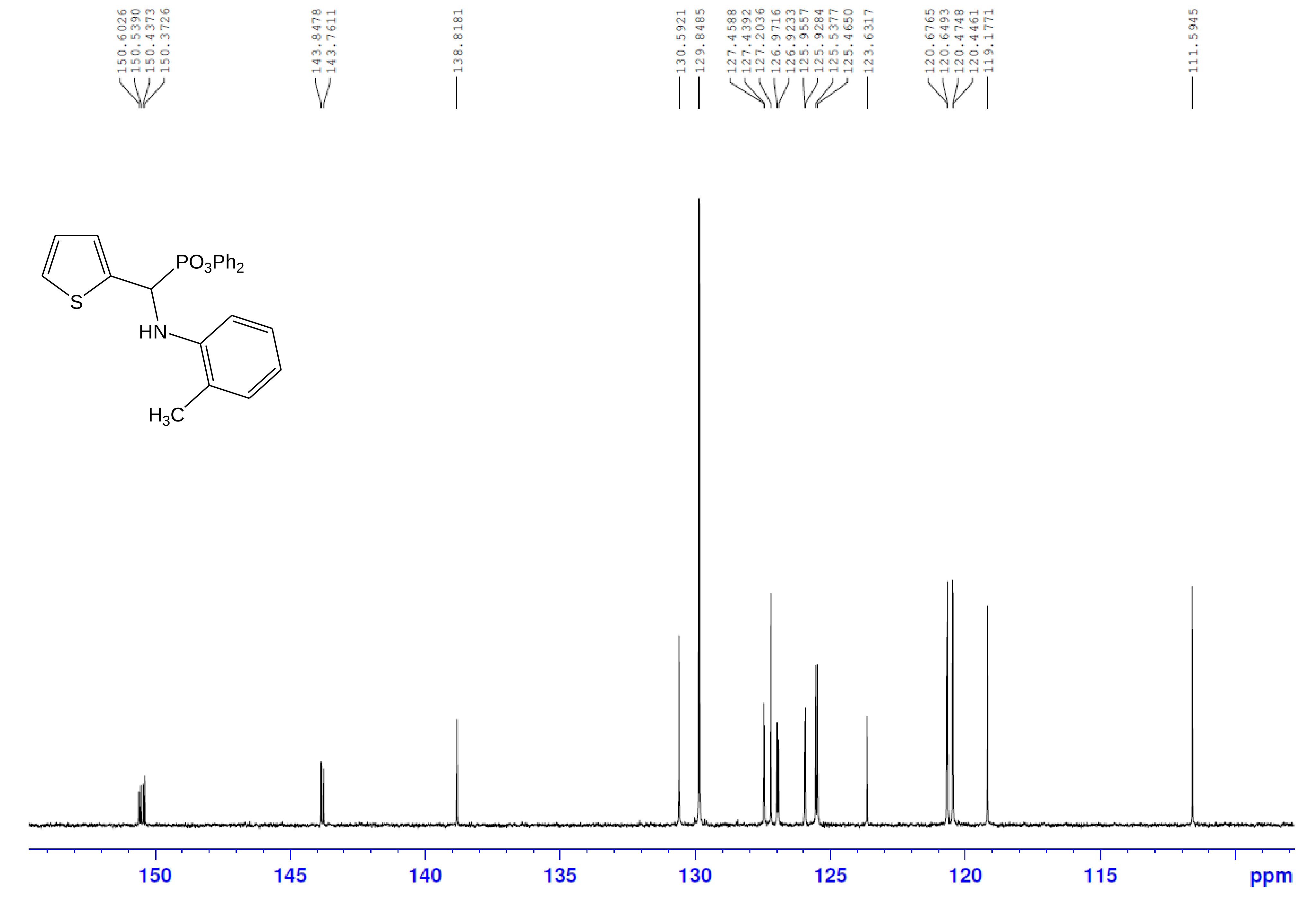
150.6026
150.5390
150.4373
150.3726
143.8478
143.7611
138.8181
130.5921
129.8485
127.4588
127.4392
127.2036
126.9716
126.9233
125.9557
125.9284
125.5377
125.4650
123.6317
120.6765
120.6493
120.4748
120.4461
119.1771
111.5945
PO3Ph2
S
HN
H3C
150
145
140
135
130
125
120
115
ppm

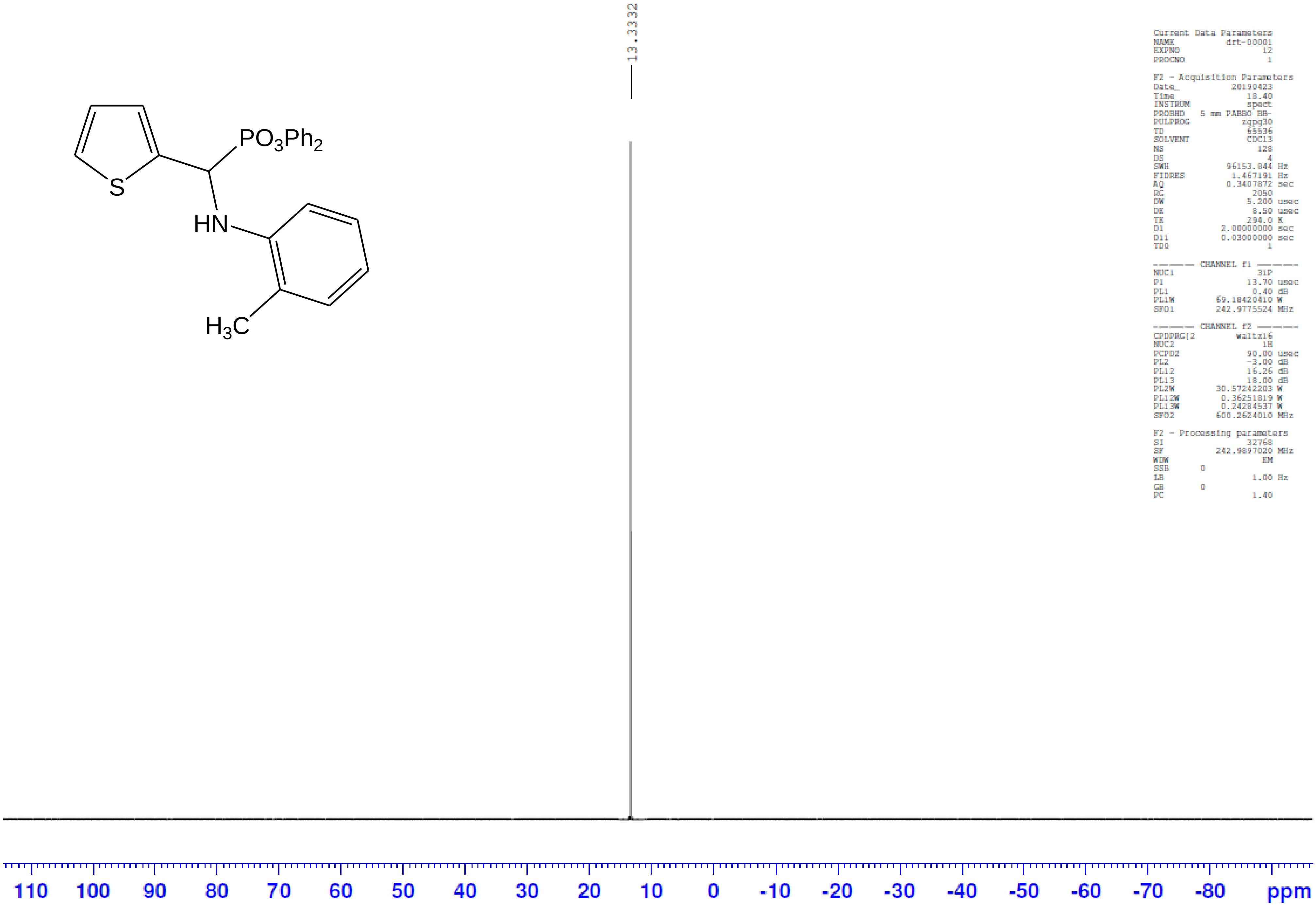
S
HN
PO_3Ph_2
H_3C
13.3332
Current Data Parameters
NAME drt-00001
EXPNO 12
PROCNO 1
F2 - Acquisition Parameters
Date_ 20190423
Time 18.40
INSTRUM spect
PROBHD 5 mm PABBO BB-
PULPROG zgpg30
TD 65536
SOLVENT CDCl3
NS 128
DS 4
SWH 96153.844 Hz
FIDRES 1.467191 Hz
AQ 0.3407872 sec
RG 2050
DW 5.200 usec
DE 8.50 usec
TE 294.0 K
D1 2.00000000 sec
D11 0.03000000 sec
TD0 1
======== CHANNEL f1 ========
NUC1 31P
P1 13.70 usec
PL1 0.40 dB
PL1W 69.18420410 W
SFO1 242.9775524 MHz
======== CHANNEL f2 ========
CPDPRG[2 waltz16
NUC2 1H
PCPD2 90.00 usec
PL2 -3.00 dB
PL12 16.26 dB
PL13 18.00 dB
PL2W 30.57242203 W
PL12W 0.36251819 W
PL13W 0.24284537 W
SFO2 600.2624010 MHz
F2 - Processing parameters
SI 32768
SF 242.9897020 MHz
WDW EM
SSB 0
LB 1.00 Hz
GB 0
PC 1.40
110 100 90 80 70 60 50 40 30 20 10 0 -10 -20 -30 -40 -50 -60 -70 -80 ppm

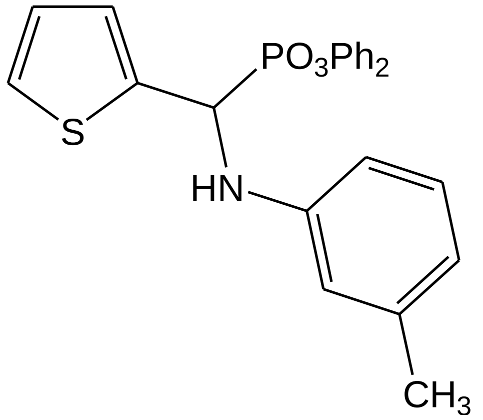

Fig. S2. ^{1}H NMR, ^{13}C NMR, ^{31}P NMR spectra of diphenyl N-(3-methylphenyl)amino(2-thienyl)methylphosphonate **(2)**

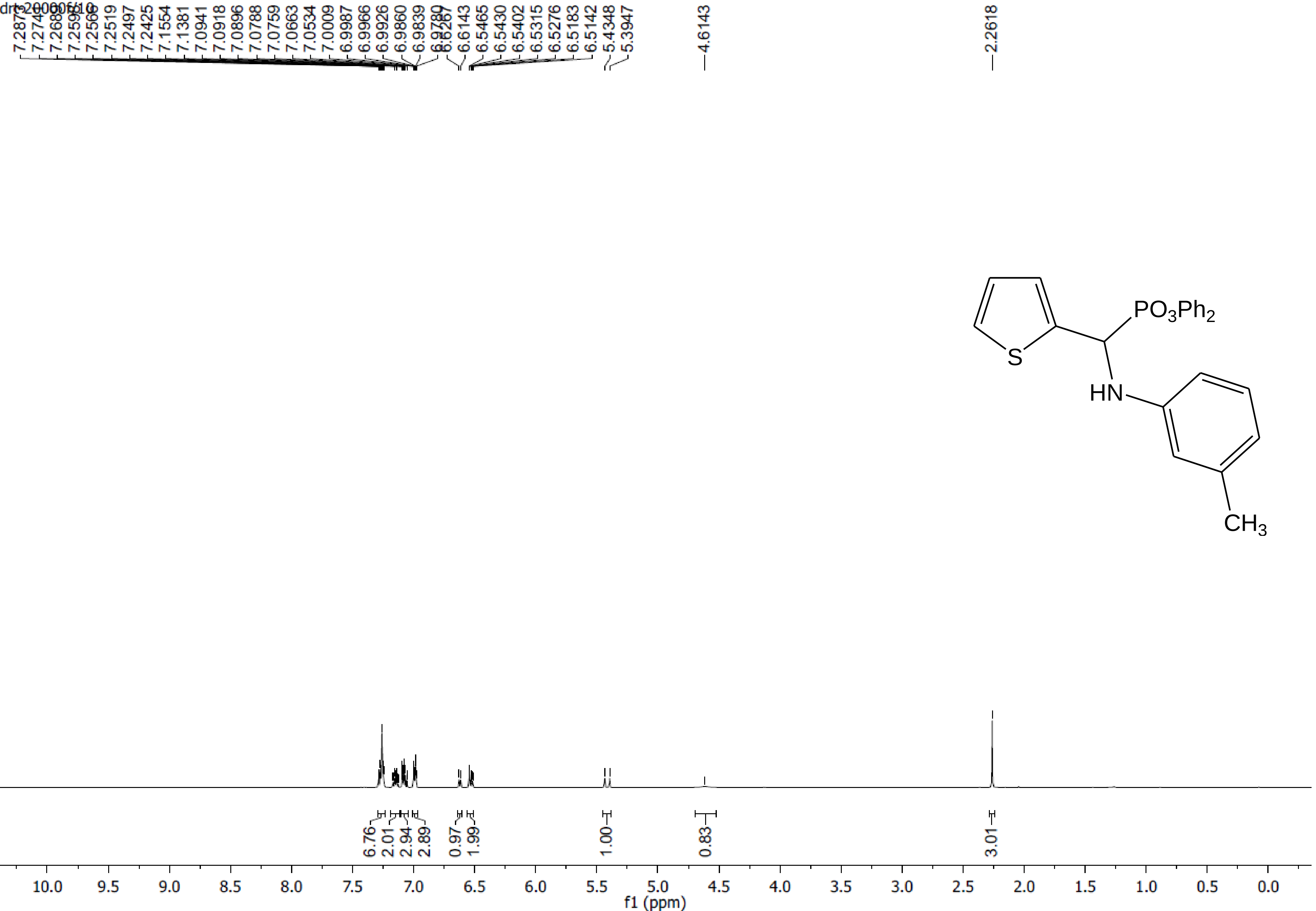

7.2519
7.2497
7.2425
7.1554
7.1381
7.0941
7.0918
7.0896
7.0788
7.0759
7.0663
7.0534
7.0009
6.9987
6.9966
6.9926
6.9860
6.9839
6.6143
6.5465
6.5430
6.5402
6.5315
6.5276
6.5183
6.5142
5.4348
5.3947
4.6143
2.2618
6.76
2.01
2.94
2.89
0.97
1.99
1.00
0.83
3.01
f1 (ppm)
PO3Ph2
S
HN
CH3

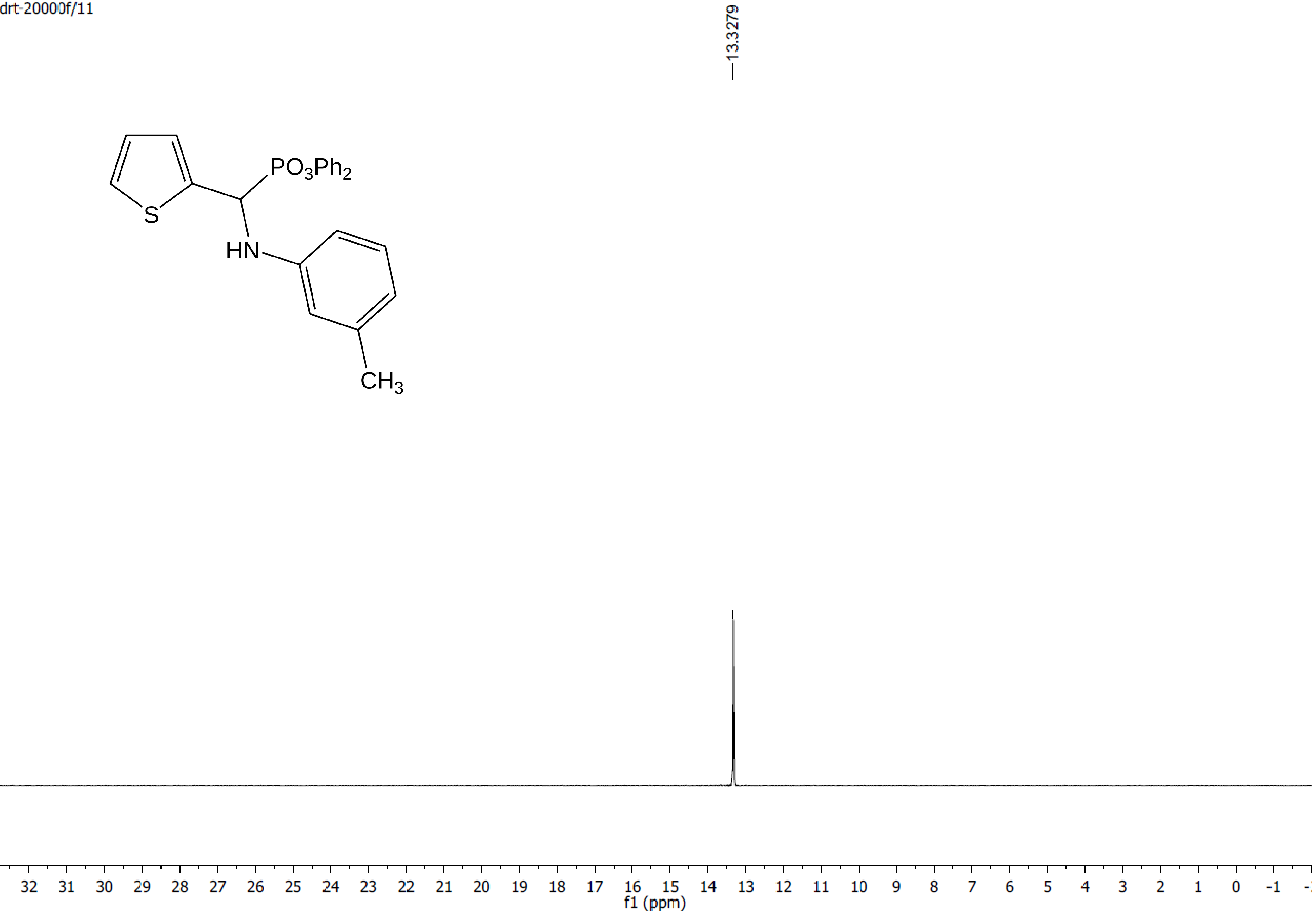
drt-20000f/11
13.3279
PO3Ph2
S
HN
CH3
32 31 30 29 28 27 26 25 24 23 22 21 20 19 18 17 16 15 14 13 12 11 10 9 8 7 6 5 4 3 2 1 0 -1
f1 (ppm)

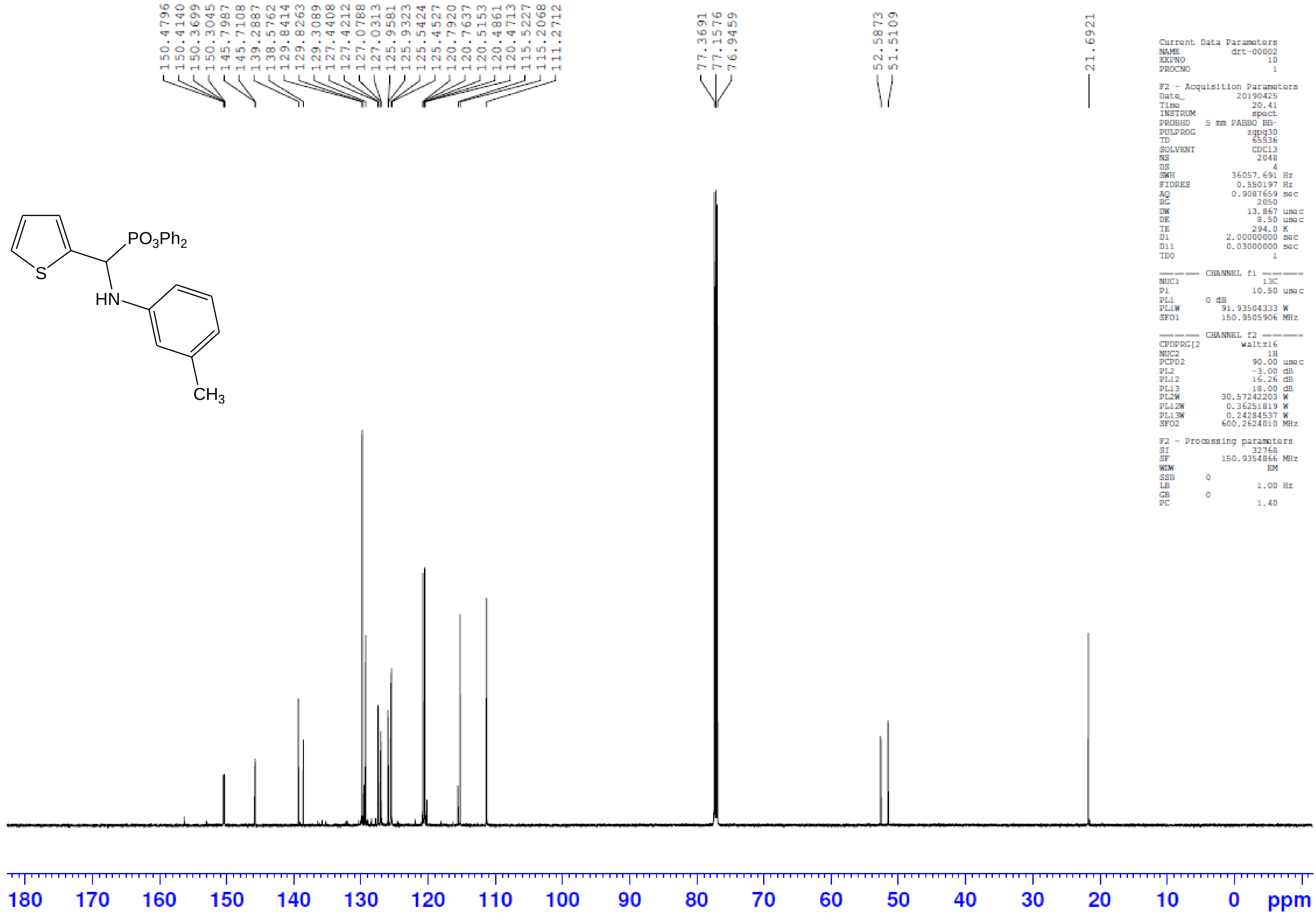
150.4796
150.4140
150.3699
150.3045
145.7987
145.7108
139.2887
138.5762
129.8414
129.8263
129.3089
127.4408
127.4212
127.0788
127.0313
125.9581
125.9323
125.5424
125.4527
120.7920
120.7637
120.5153
120.4861
120.4713
115.5227
115.2068
111.2712
77.3691
77.1576
76.9459
52.5873
51.5109
21.6921
PO3Ph2
S
HN
CH3
Current Data Parameters
NAME drt-00002
EXPNO 10
PROCNO 1
F2 - Acquisition Parameters
Date_ 20190425
Time 20.41
INSTRUM spect
PROBHD 5 mm PABBO BB-
PULPROG zgpg30
TD 65536
SOLVENT CDCl3
NS 2048
DS 4
SWH 36057.691 Hz
FIDRES 0.550197 Hz
AQ 0.9087659 sec
RG 2050
DW 13.867 usec
DE 8.50 usec
TE 294.0 K
D1 2.00000000 sec
D11 0.03000000 sec
TD0 1
CHANNEL f1
NUC1 13C
P1 10.50 usec
PL1 0 dB
PL1W 91.93504333 W
SFO1 150.9505906 MHz
CHANNEL f2
CPDPRG[2 waltz16
NUC2 1H
PCPD2 90.00 usec
PL2 -3.00 dB
PL12 16.26 dB
PL13 18.00 dB
PL2W 30.57242203 W
PL12W 0.36251819 W
PL13W 0.24284537 W
SFO2 600.2624010 MHz
F2 - Processing parameters
SI 32768
SF 150.9354866 MHz
WDW EM
SSB 0
LB 1.00 Hz
GB 0
PC 1.40
180
170
160
150
140
130
120
110
100
90
80
70
60
50
40
30
20
10
0
ppm

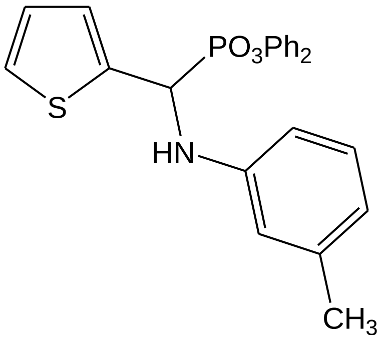
PO3Ph2
S
HN
CH3

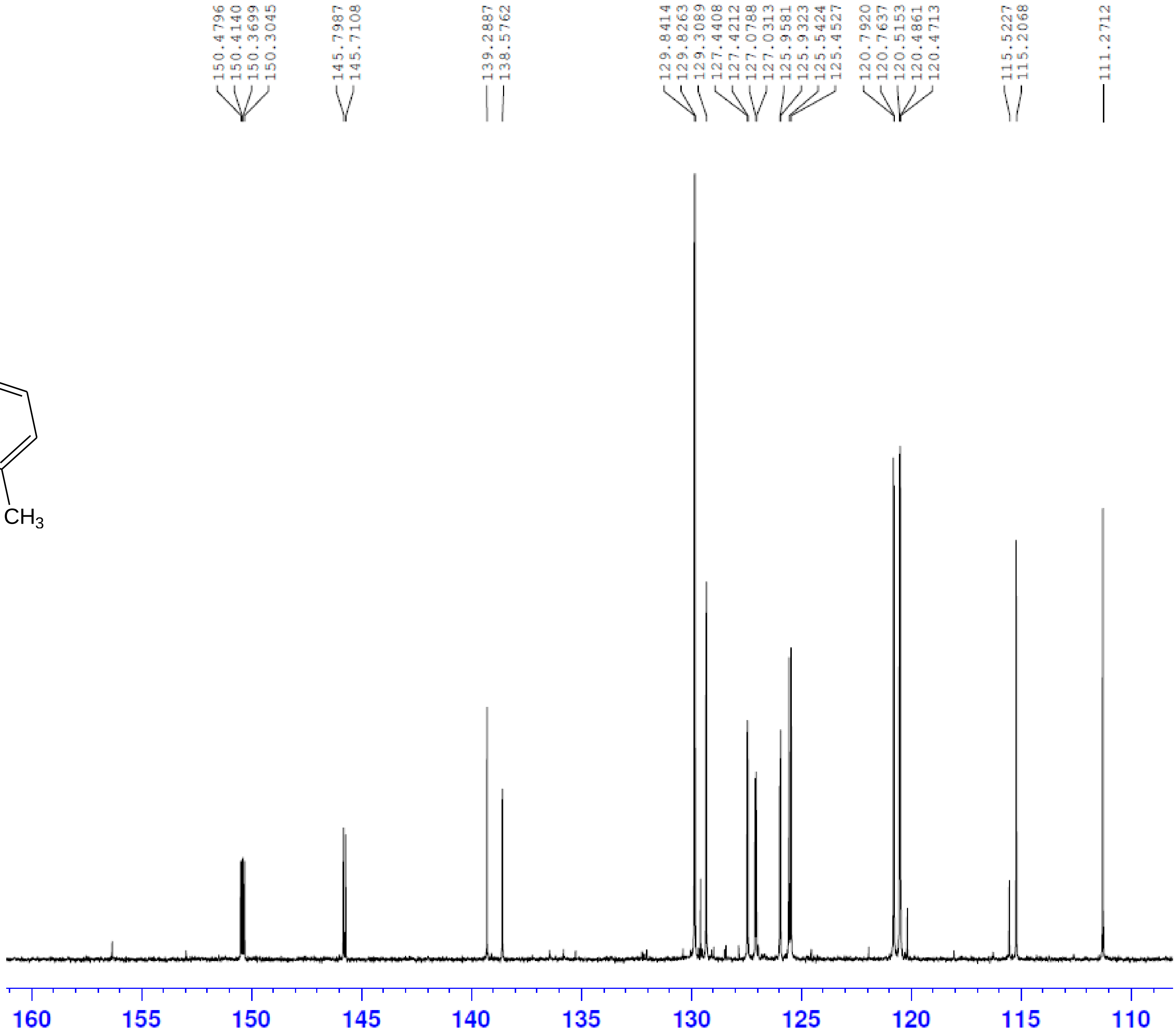
150.4796
150.4140
150.3699
150.3045
145.7987
145.7108
139.2887
138.5762
129.8414
129.8263
129.3089
127.4408
127.4212
127.0788
127.0313
125.9581
125.9323
125.5424
125.4527
120.7920
120.7637
120.5153
120.4861
120.4713
115.5227
115.2068
111.2712
160
155
150
145
140
135
130
125
120
115
110

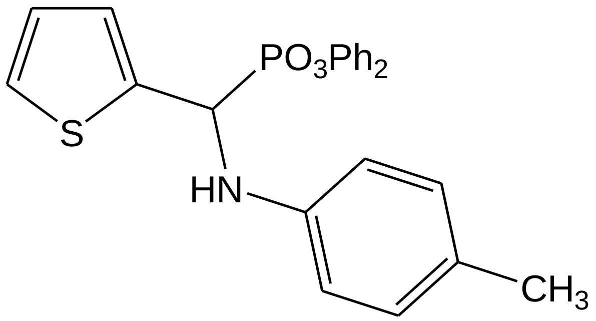

Fig. S3. ^{1}H NMR, ^{13}C NMR, ^{31}P NMR spectra of diphenyl N-(4-methylphenyl)amino(2-thienyl)methylphosphonate **(3)**

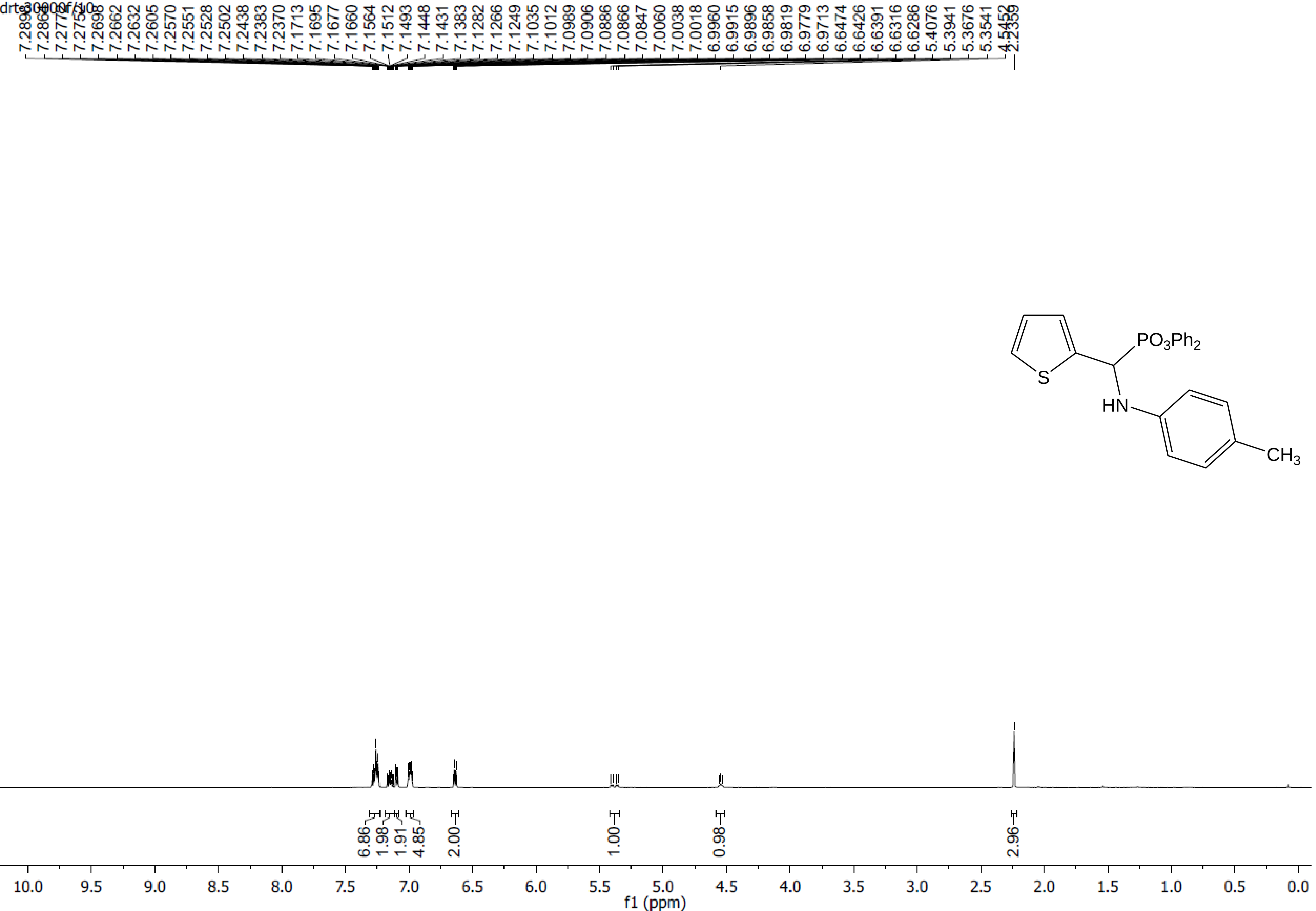

7.2662
7.2632
7.2605
7.2570
7.2551
7.2528
7.2502
7.2438
7.2383
7.2370
7.1713
7.1695
7.1677
7.1660
7.1564
7.1512
7.1493
7.1448
7.1431
7.1383
7.1282
7.1266
7.1249
7.1035
7.1012
7.0989
7.0906
7.0886
7.0866
7.0847
7.0060
7.0038
7.0018
6.9960
6.9915
6.9896
6.9858
6.9819
6.9779
6.9713
6.6474
6.6426
6.6391
6.6316
6.6286
5.4076
5.3941
5.3676
5.3541
PO3Ph2
S
HN
CH3
6.86
1.98
1.91
4.85
2.00
1.00
0.98
2.96
10.0
9.5
9.0
8.5
8.0
7.5
7.0
6.5
6.0
5.5
5.0
4.5
4.0
3.5
3.0
2.5
2.0
1.5
1.0
0.5
0.0
f1 (ppm)

drt-30000f/10

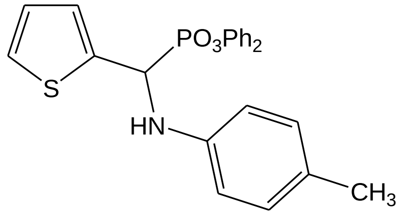

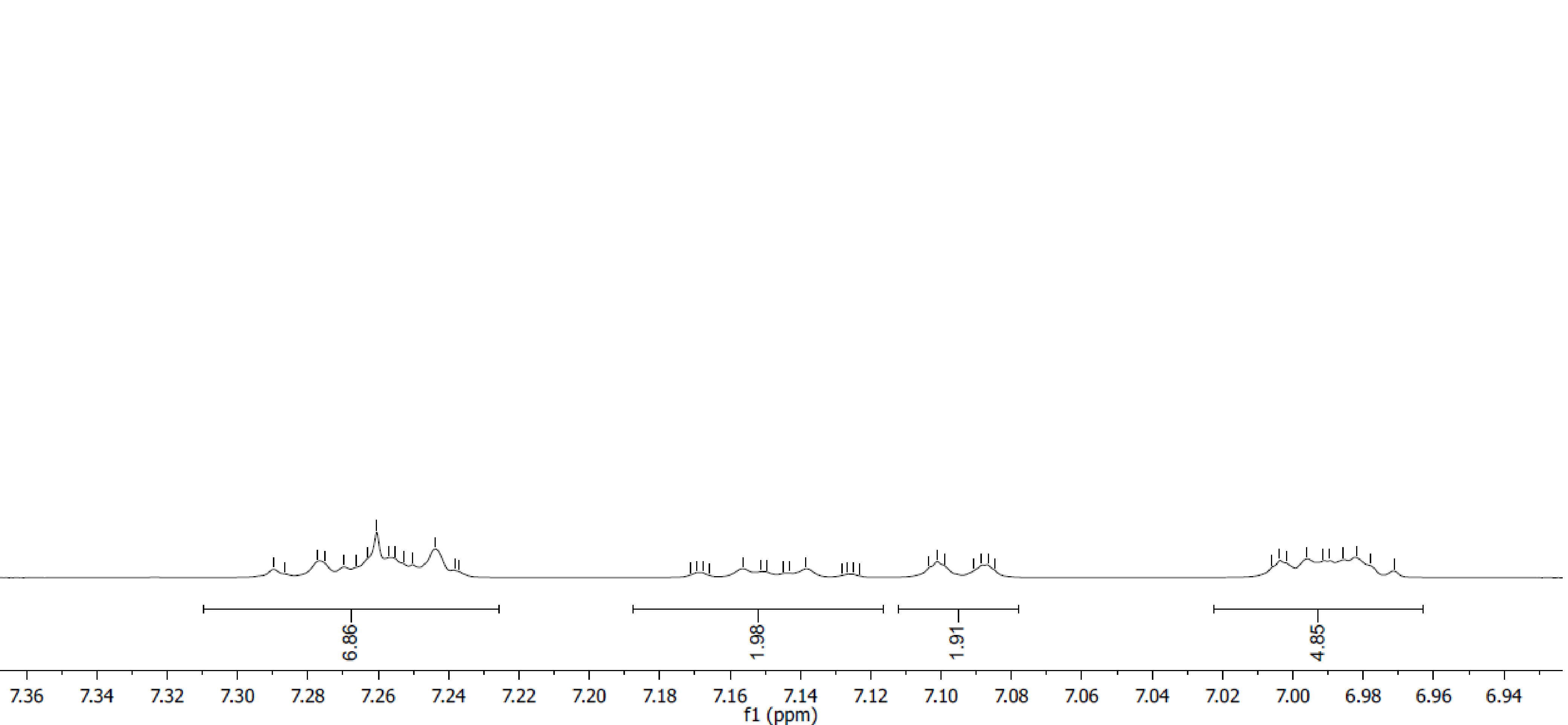

drt-30000f/10

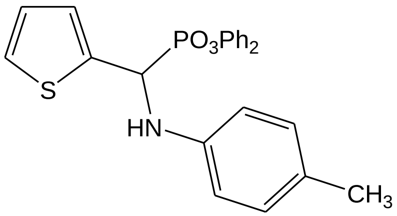

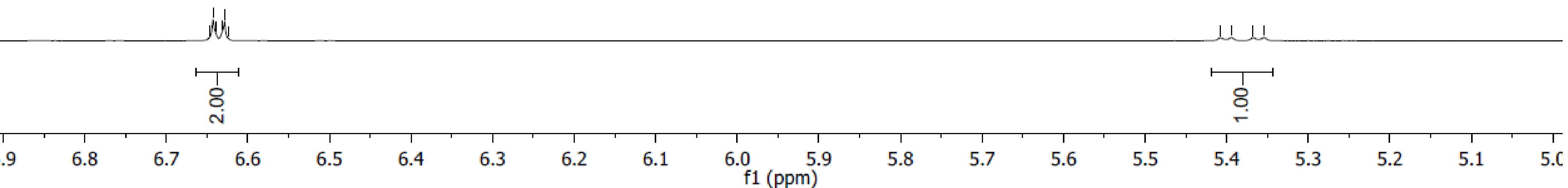

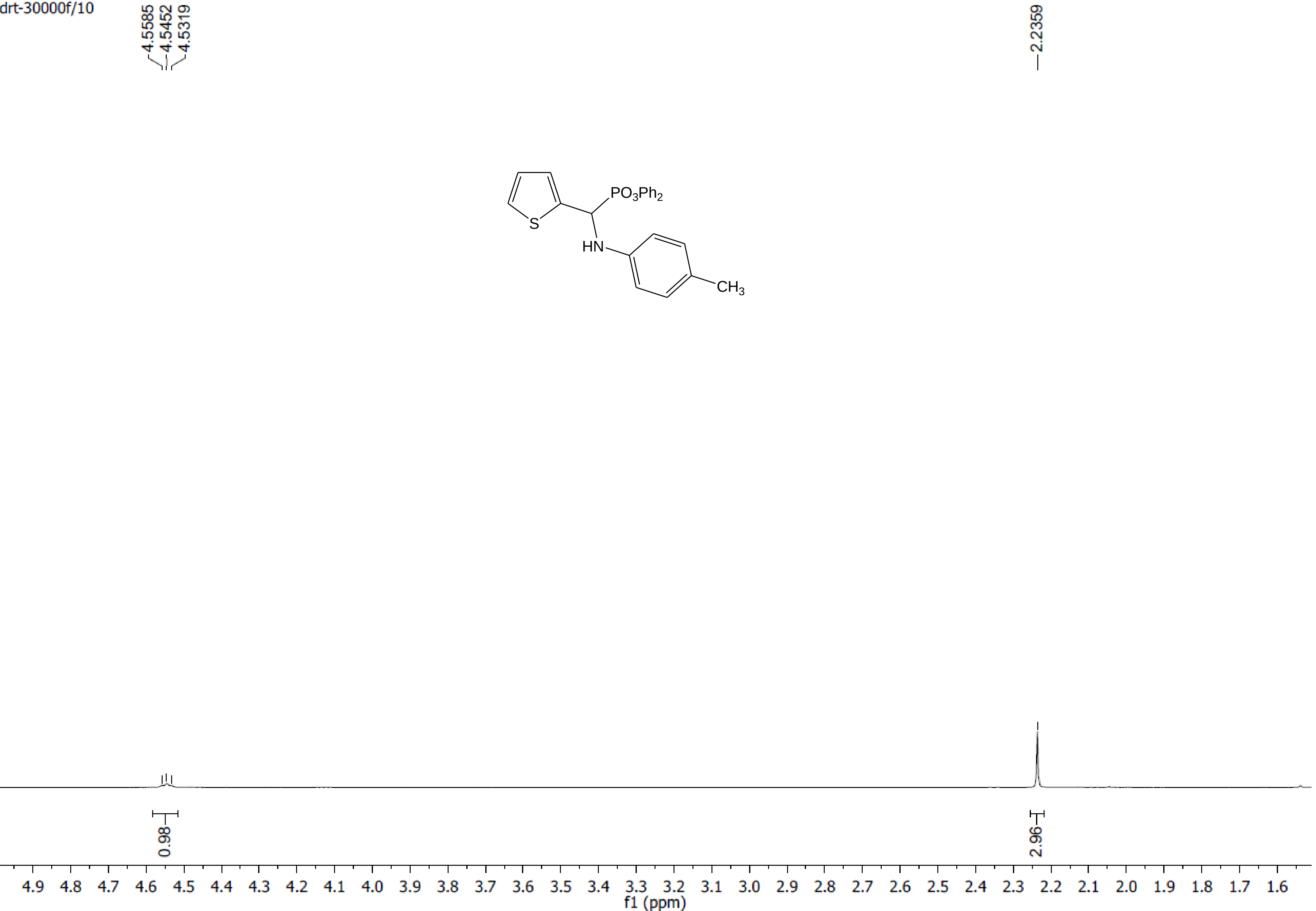
drt-30000f/10
4.5585
4.5452
4.5319
2.2359
PO3Ph2
S
HN
CH3
0.98
2.96
4.9 4.8 4.7 4.6 4.5 4.4 4.3 4.2 4.1 4.0 3.9 3.8 3.7 3.6 3.5 3.4 3.3 3.2 3.1 3.0 2.9 2.8 2.7 2.6 2.5 2.4 2.3 2.2 2.1 2.0 1.9 1.8 1.7 1.6
f1 (ppm)

drt-00003f/11

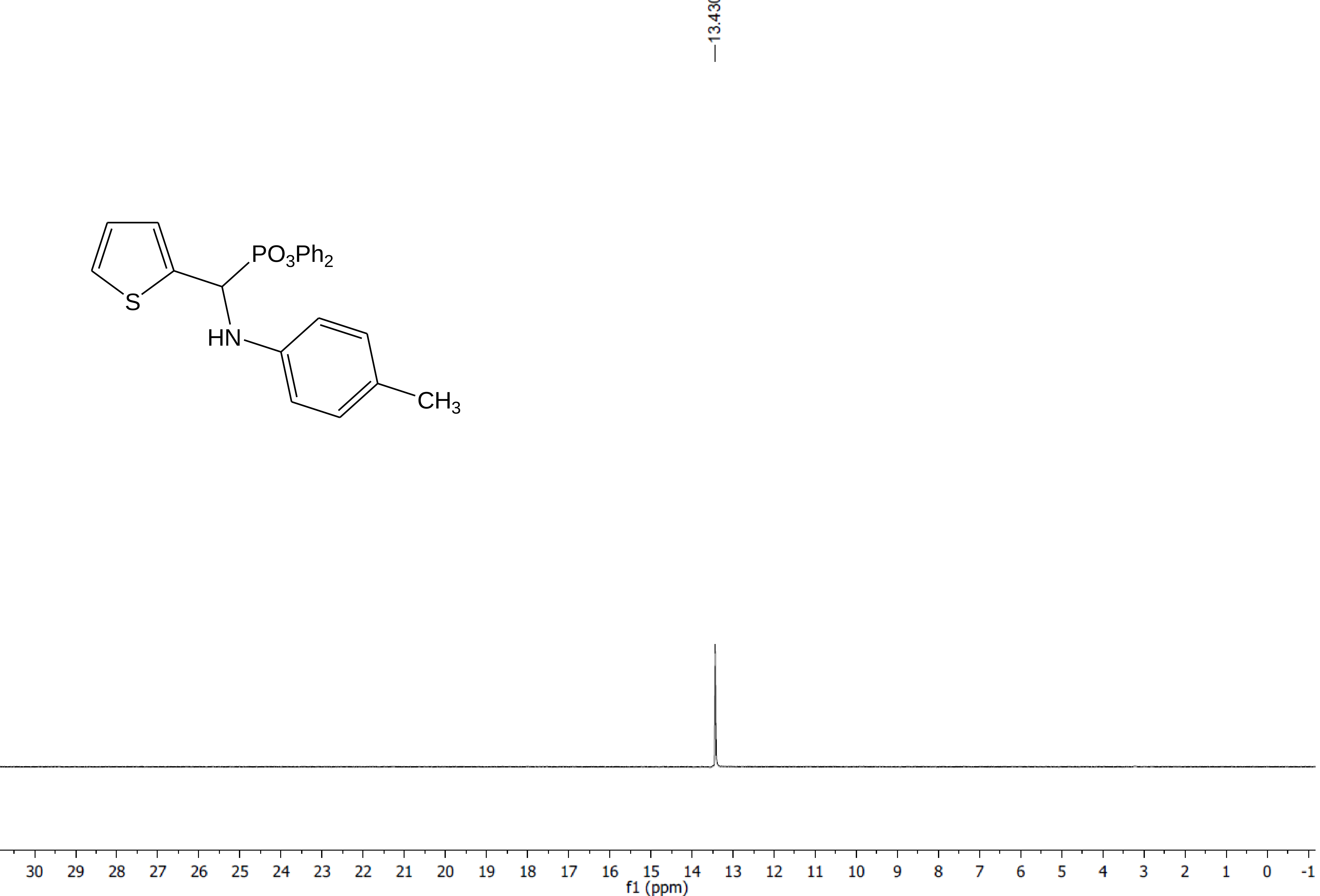

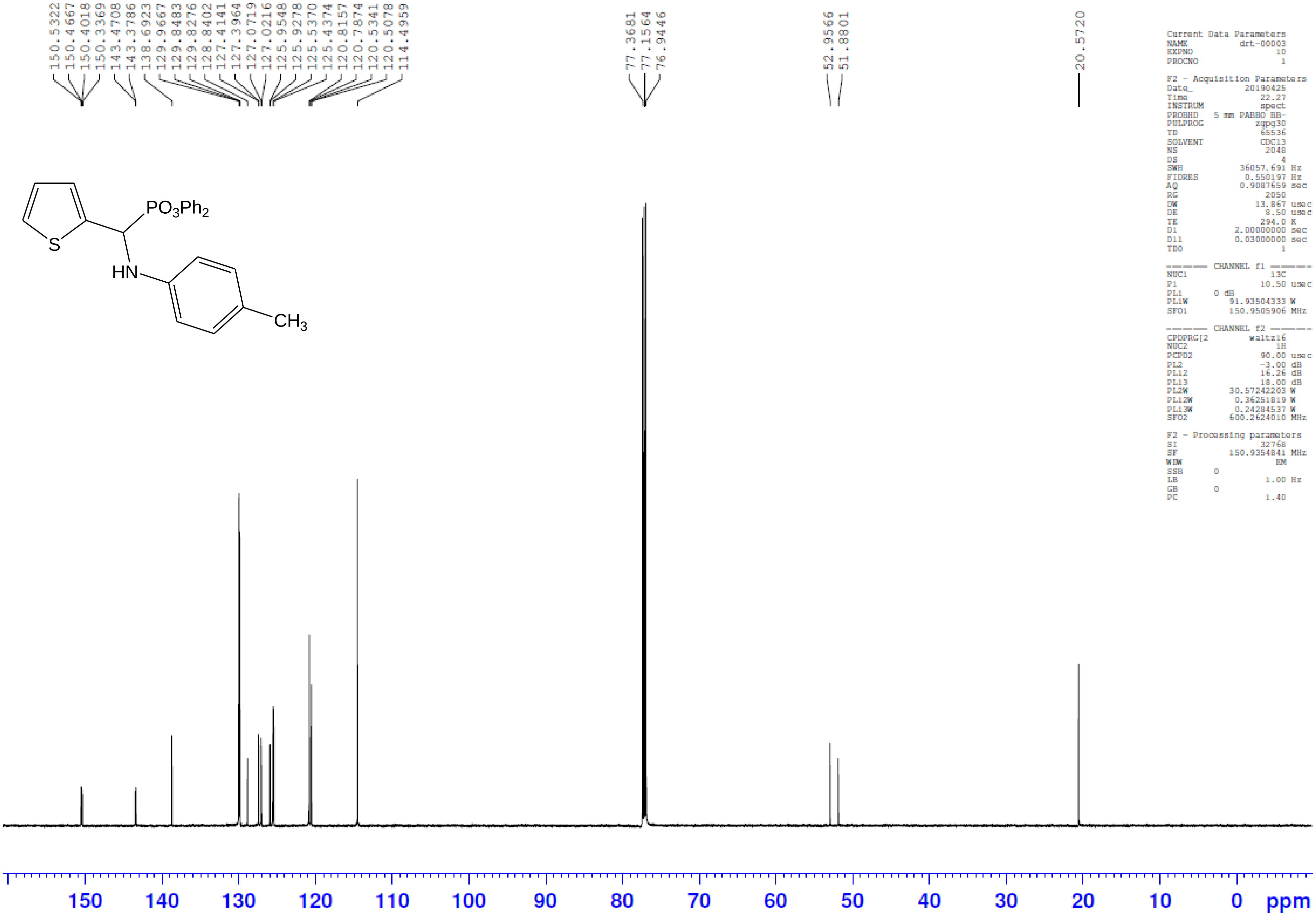
150.5322
150.4667
150.4018
150.3369
143.4708
143.3786
138.6923
129.9667
129.8483
129.8276
128.8402
127.4141
127.3964
127.0719
127.0216
125.9548
125.9278
125.5370
125.4374
120.8157
120.7874
120.5341
120.5078
114.4959
77.3681
77.1564
76.9446
52.9566
51.8801
20.5720
PO3Ph2
S
HN
CH3
Current Data Parameters
NAME drt-00003
EXPNO 10
PROCNO 1
F2 - Acquisition Parameters
Date_ 20190425
Time 22.27
INSTRUM spect
PROBHD 5 mm PABBO BB-
PULPROG zgpg30
TD 65536
SOLVENT CDCl3
NS 2048
DS 4
SWH 36057.691 Hz
FIDRES 0.550197 Hz
AQ 0.9087659 sec
RG 2050
DW 13.867 usec
DE 8.50 usec
TE 294.0 K
D1 2.00000000 sec
D11 0.03000000 sec
TD0 1
======== CHANNEL f1 ========
NUC1 13C
P1 10.50 usec
PL1 0 dB
PL1W 91.93504333 W
SFO1 150.9505906 MHz
======== CHANNEL f2 ========
CPDPRG[2 waltz16
NUC2 1H
PCPD2 90.00 usec
PL2 -3.00 dB
PL12 16.26 dB
PL13 18.00 dB
PL2W 30.57242203 W
PL12W 0.36251819 W
PL13W 0.24284537 W
SFO2 600.2624010 MHz
F2 - Processing parameters
SI 32768
SF 150.9354841 MHz
WDW EM
SSB 0
LB 1.00 Hz
GB 0
PC 1.40
150
140
130
120
110
100
90
80
70
60
50
40
30
20
10
0
ppm

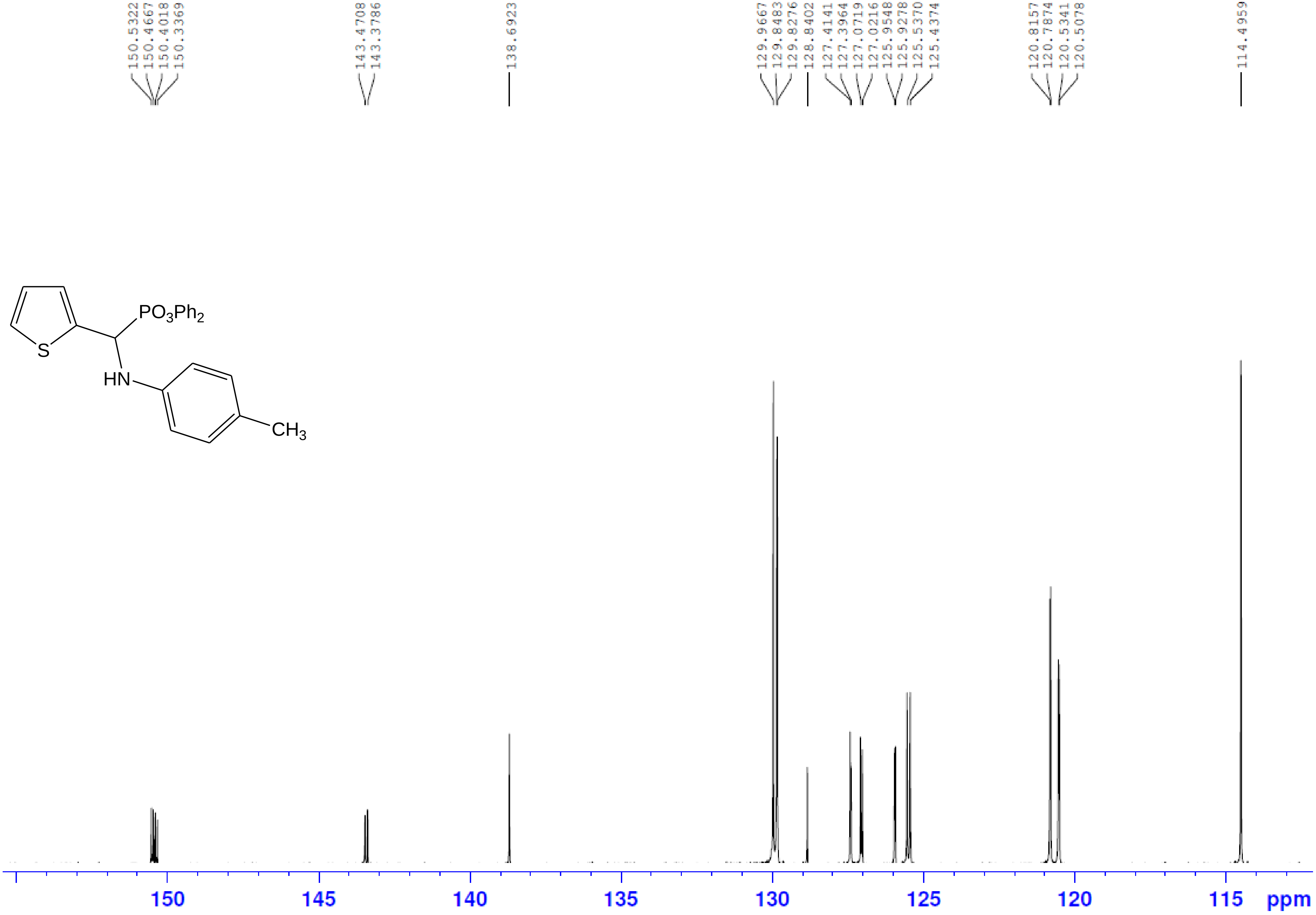
150.5322
150.4667
150.4018
150.3369
143.4708
143.3786
138.6923
129.9667
129.8483
129.8276
128.8402
127.4141
127.3964
127.0719
127.0216
125.9548
125.9278
125.5370
125.4374
120.8157
120.7874
120.5341
120.5078
114.4959
PO3Ph2
S
HN
CH3
150
145
140
135
130
125
120
115
ppm

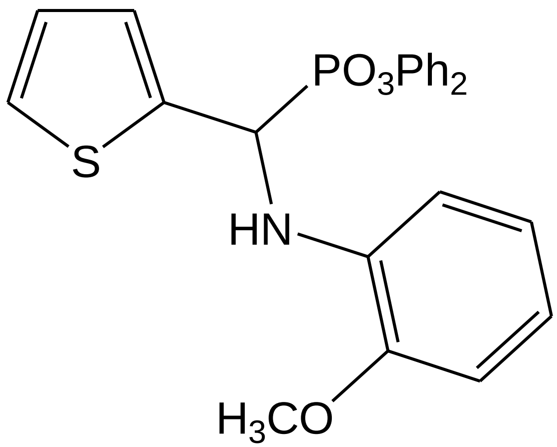

Fig. S4. ^{1}H NMR, ^{13}C NMR, ^{31}P NMR spectra of diphenyl N-(2-methoxyphenyl)amino(2-thienyl)methylphosphonate **(4)**

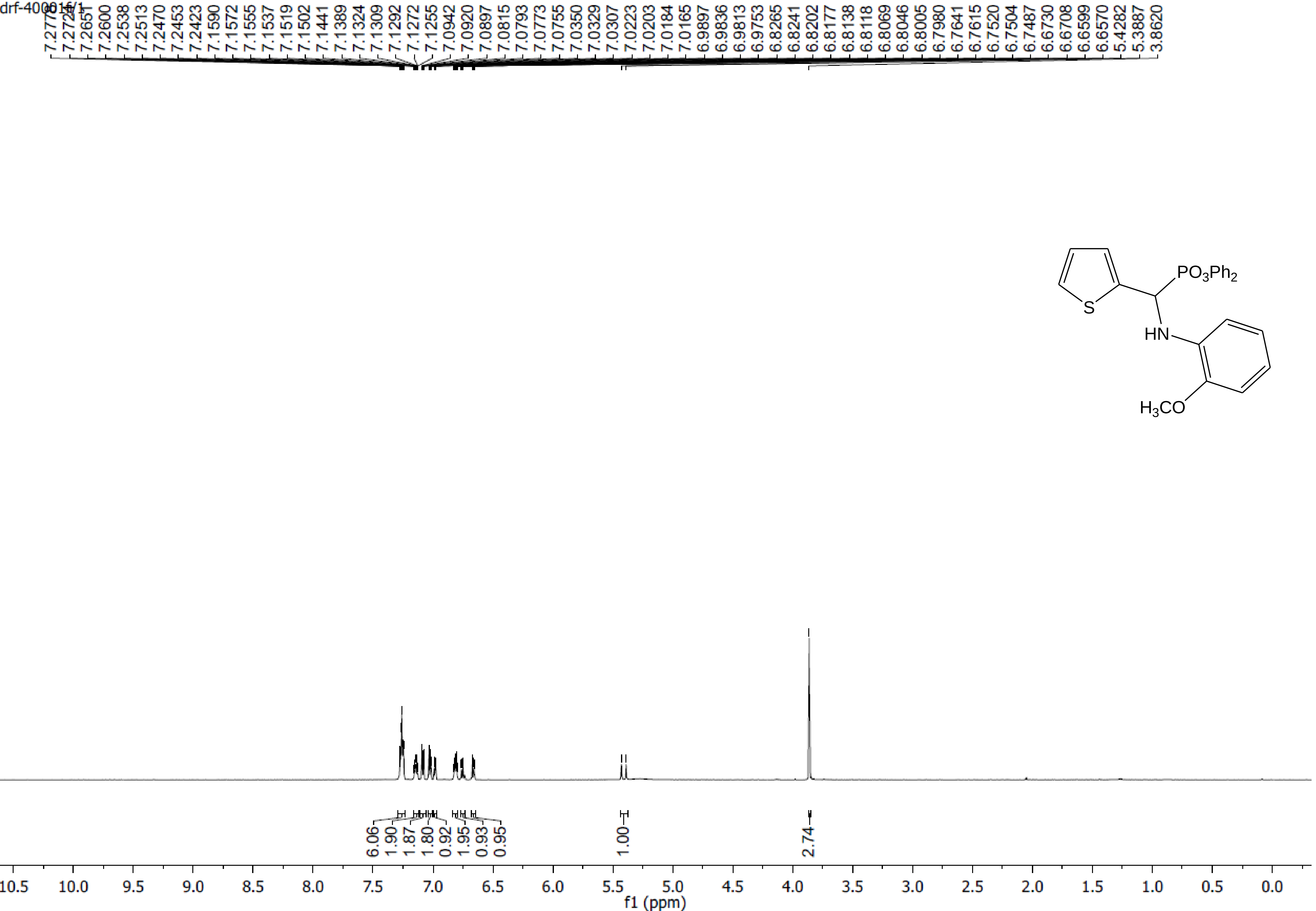
7.2773
7.2726
7.2651
7.2600
7.2538
7.2513
7.2470
7.2453
7.2423
7.1590
7.1572
7.1555
7.1537
7.1519
7.1502
7.1441
7.1389
7.1324
7.1309
7.1292
7.1272
7.1255
7.0942
7.0920
7.0897
7.0815
7.0793
7.0773
7.0755
7.0350
7.0329
7.0307
7.0223
7.0203
7.0184
7.0165
6.9897
6.9836
6.9813
6.9753
6.8265
6.8241
6.8202
6.8177
6.8138
6.8118
6.8069
6.8046
6.8005
6.7980
6.7641
6.7615
6.7520
6.7504
6.7487
6.6730
6.6708
6.6599
6.6570
5.4282
5.3887
3.8620
PO_3Ph_2
S
HN
H_3CO
6.06
1.90
1.87
1.80
0.92
1.95
0.93
0.95
1.00
2.74
10.5 10.0 9.5 9.0 8.5 8.0 7.5 7.0 6.5 6.0 5.5 5.0 4.5 4.0 3.5 3.0 2.5 2.0 1.5 1.0 0.5 0.0
f1 (ppm)

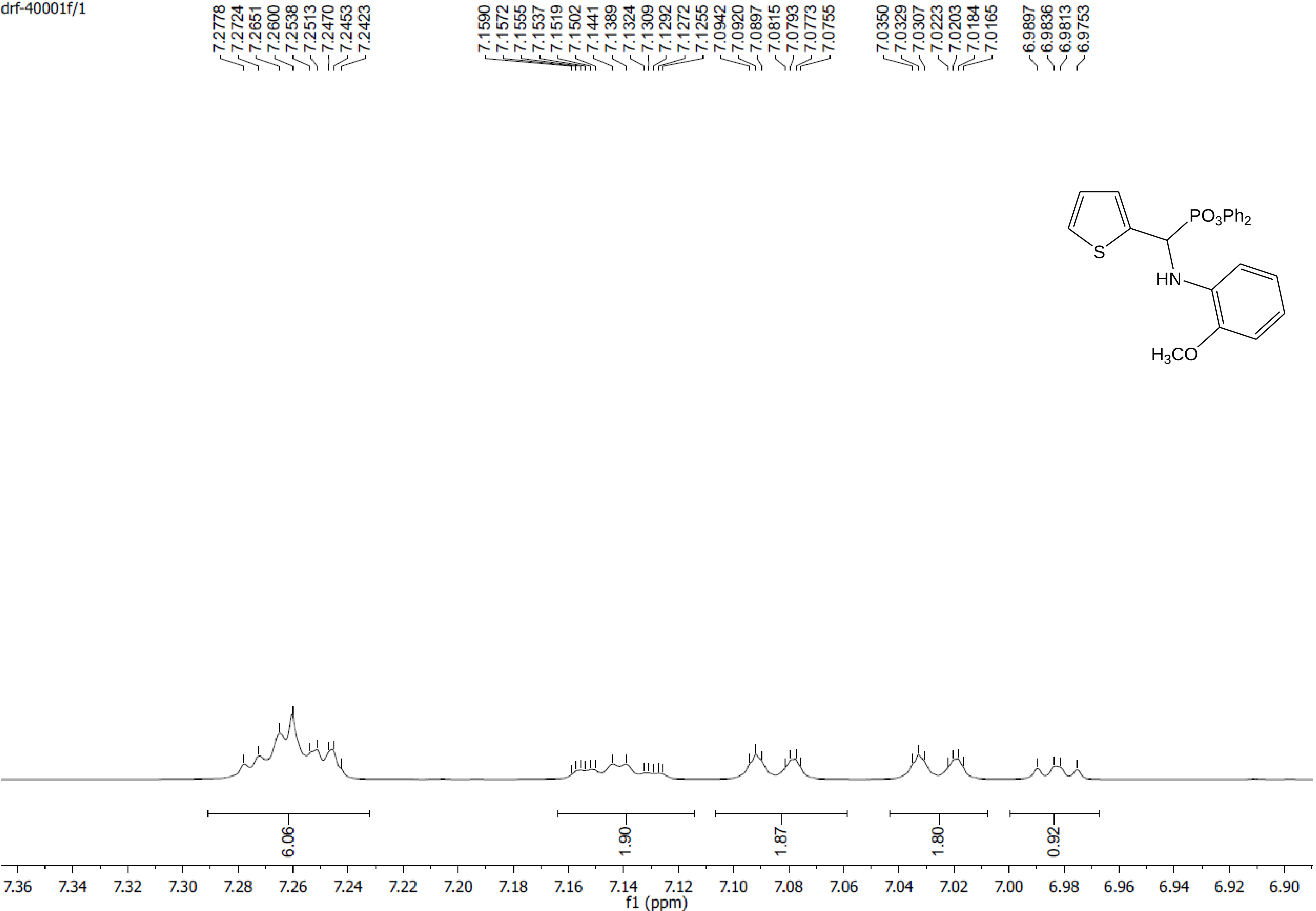
drf-40001f/1
7.2778
7.2724
7.2651
7.2600
7.2538
7.2513
7.2470
7.2453
7.2423
7.1590
7.1572
7.1555
7.1537
7.1519
7.1502
7.1441
7.1389
7.1324
7.1309
7.1292
7.1272
7.1255
7.0942
7.0920
7.0897
7.0815
7.0793
7.0773
7.0755
7.0350
7.0329
7.0307
7.0223
7.0203
7.0184
7.0165
6.9897
6.9836
6.9813
6.9753
PO3Ph2
S
HN
H3CO
6.06
1.90
1.87
1.80
0.92
7.36 7.34 7.32 7.30 7.28 7.26 7.24 7.22 7.20 7.18 7.16 7.14 7.12 7.10 7.08 7.06 7.04 7.02 7.00 6.98 6.96 6.94 6.92 6.90
f1 (ppm)

drf-40001f/1

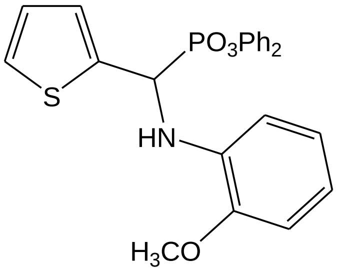
PO3Ph2
S
HN
H3CO

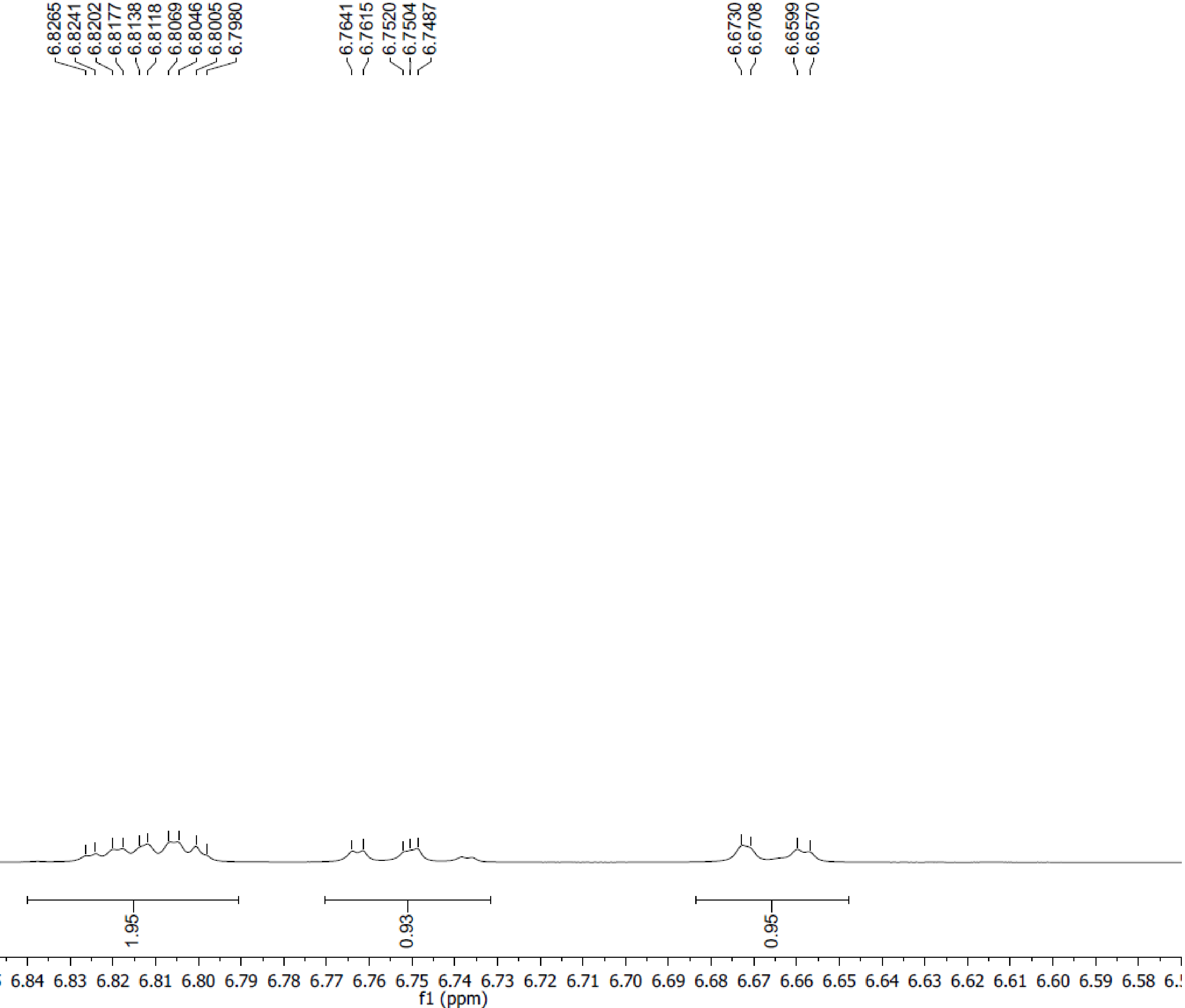
6.8265
6.8241
6.8202
6.8177
6.8138
6.8118
6.8069
6.8046
6.8005
6.7980
6.7641
6.7615
6.7520
6.7504
6.7487
6.6730
6.6708
6.6599
6.6570
1.95
0.93
0.95
6.91 6.90 6.89 6.88 6.87 6.86 6.85 6.84 6.83 6.82 6.81 6.80 6.79 6.78 6.77 6.76 6.75 6.74 6.73 6.72 6.71 6.70 6.69 6.68 6.67 6.66 6.65 6.64 6.63 6.62 6.61 6.60 6.59 6.58
f1 (ppm)

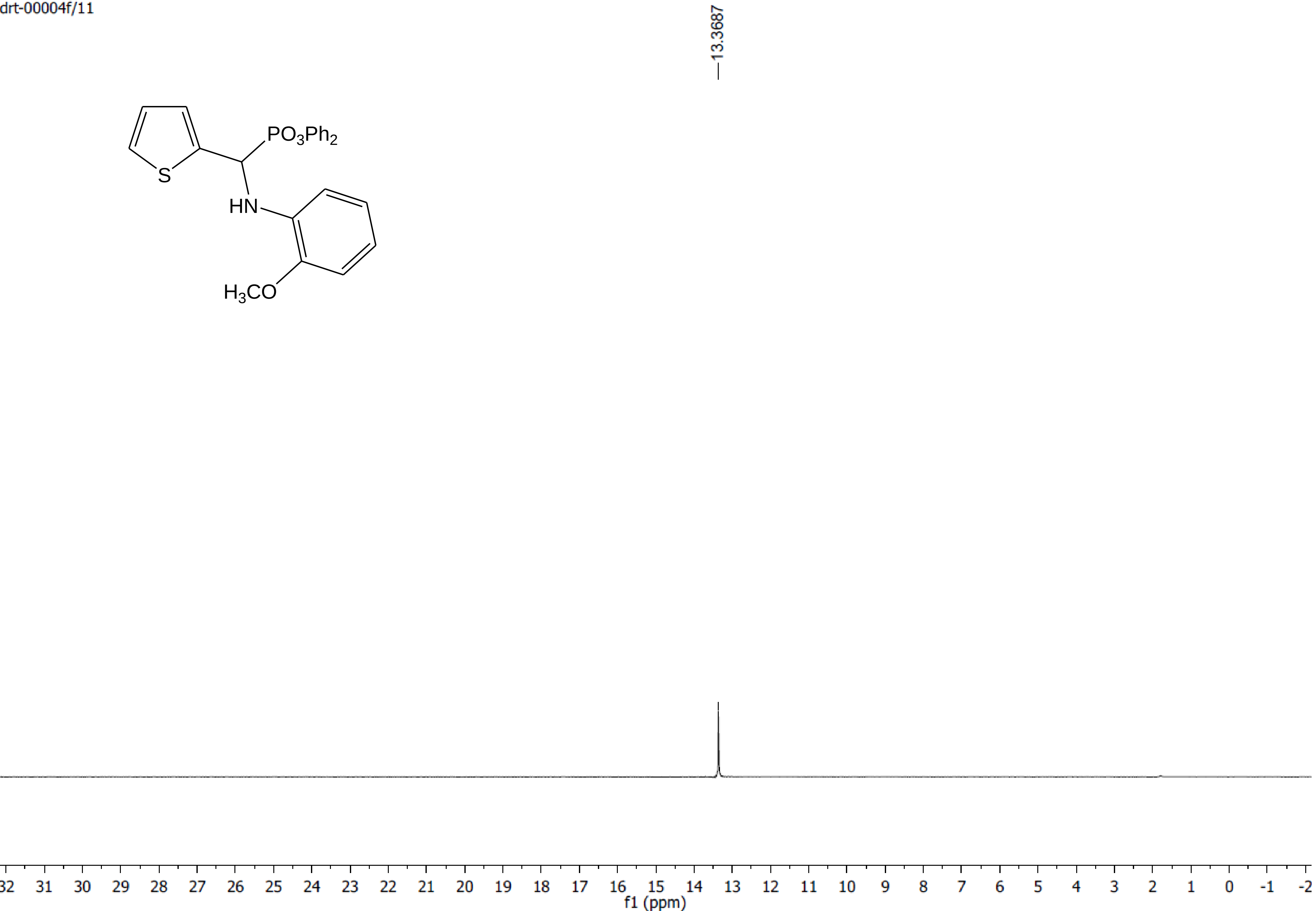
drt-00004f/11
13.3687
PO_3Ph_2
S
HN
H_3CO
32 31 30 29 28 27 26 25 24 23 22 21 20 19 18 17 16 15 14 13 12 11 10 9 8 7 6 5 4 3 2 1 0 -1 -2
f1 (ppm)

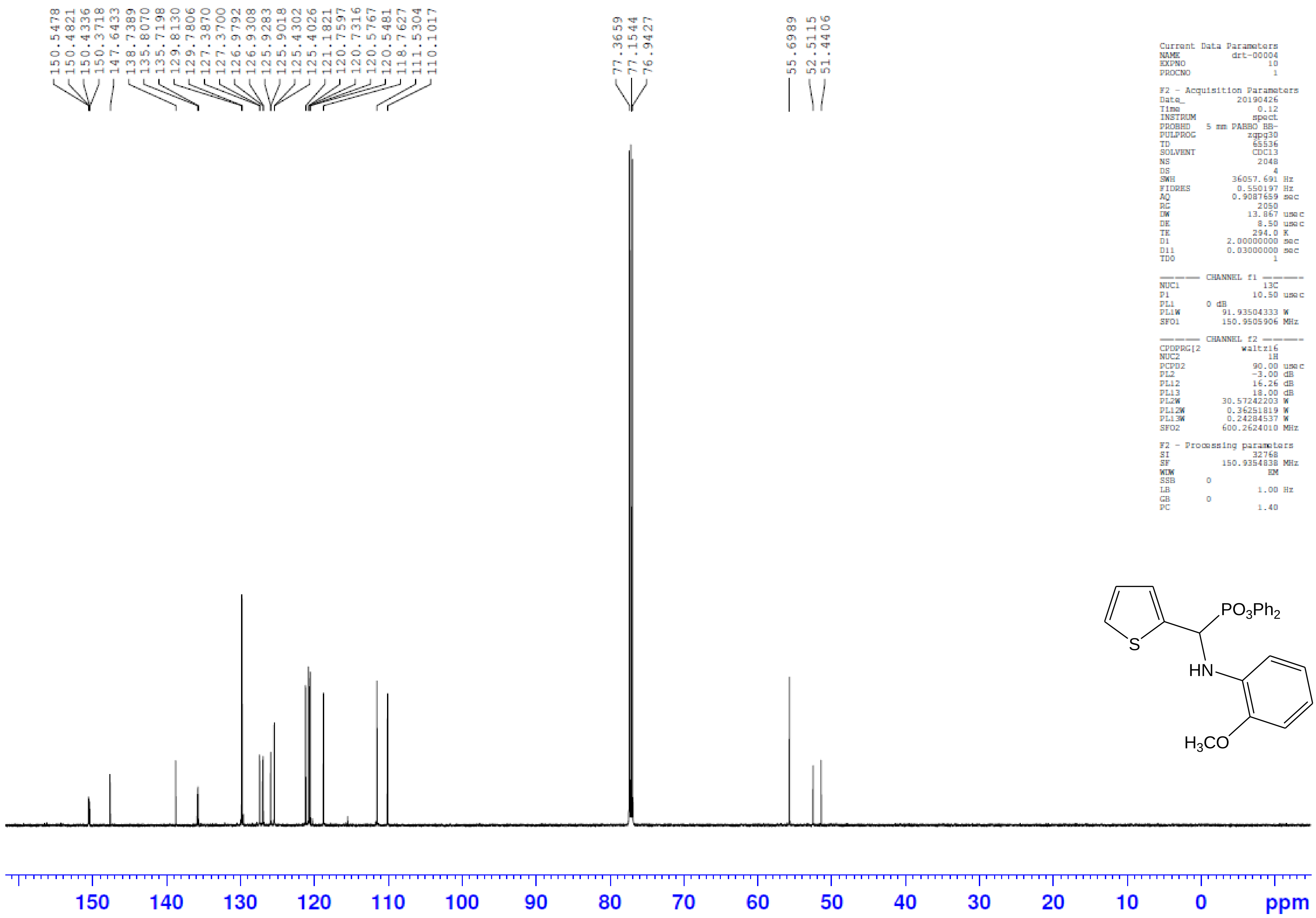
150.5478
150.4821
150.4336
150.3718
147.6433
138.7389
135.8070
135.7198
129.8130
129.7806
127.3870
127.3700
126.9792
126.9308
125.9283
125.9018
125.4302
125.4026
121.1821
120.7597
120.7316
120.5767
120.5481
118.7627
111.5304
110.1017
77.3659
77.1544
76.9427
55.6989
52.5115
51.4406
Current Data Parameters
NAME drt-00004
EXPNO 10
PROCNO 1
F2 - Acquisition Parameters
Date_ 20190426
Time 0.12
INSTRUM spect
PROBHD 5 mm PABBO BB-
PULPROG zgpg30
TD 65536
SOLVENT CDCl3
NS 2048
DS 4
SWH 36057.691 Hz
FIDRES 0.550197 Hz
AQ 0.9087659 sec
RG 2050
DW 13.867 usec
DE 8.50 usec
TE 294.0 K
D1 2.00000000 sec
D11 0.03000000 sec
TD0 1
CHANNEL f1
NUC1 13C
P1 10.50 usec
PL1 0 dB
PL1W 91.93504333 W
SFO1 150.9505906 MHz
CHANNEL f2
CPDPRG[2 waltz16
NUC2 1H
PCPD2 90.00 usec
PL2 -3.00 dB
PL12 16.26 dB
PL13 18.00 dB
PL2W 30.57242203 W
PL12W 0.36251819 W
PL13W 0.24284537 W
SFO2 600.2624010 MHz
F2 - Processing parameters
SI 32768
SF 150.9354838 MHz
WDW EM
SSB 0
LB 1.00 Hz
GB 0
PC 1.40
150
140
130
120
110
100
90
80
70
60
50
40
30
20
10
0
ppm
PO3Ph2
S
HN
H3CO

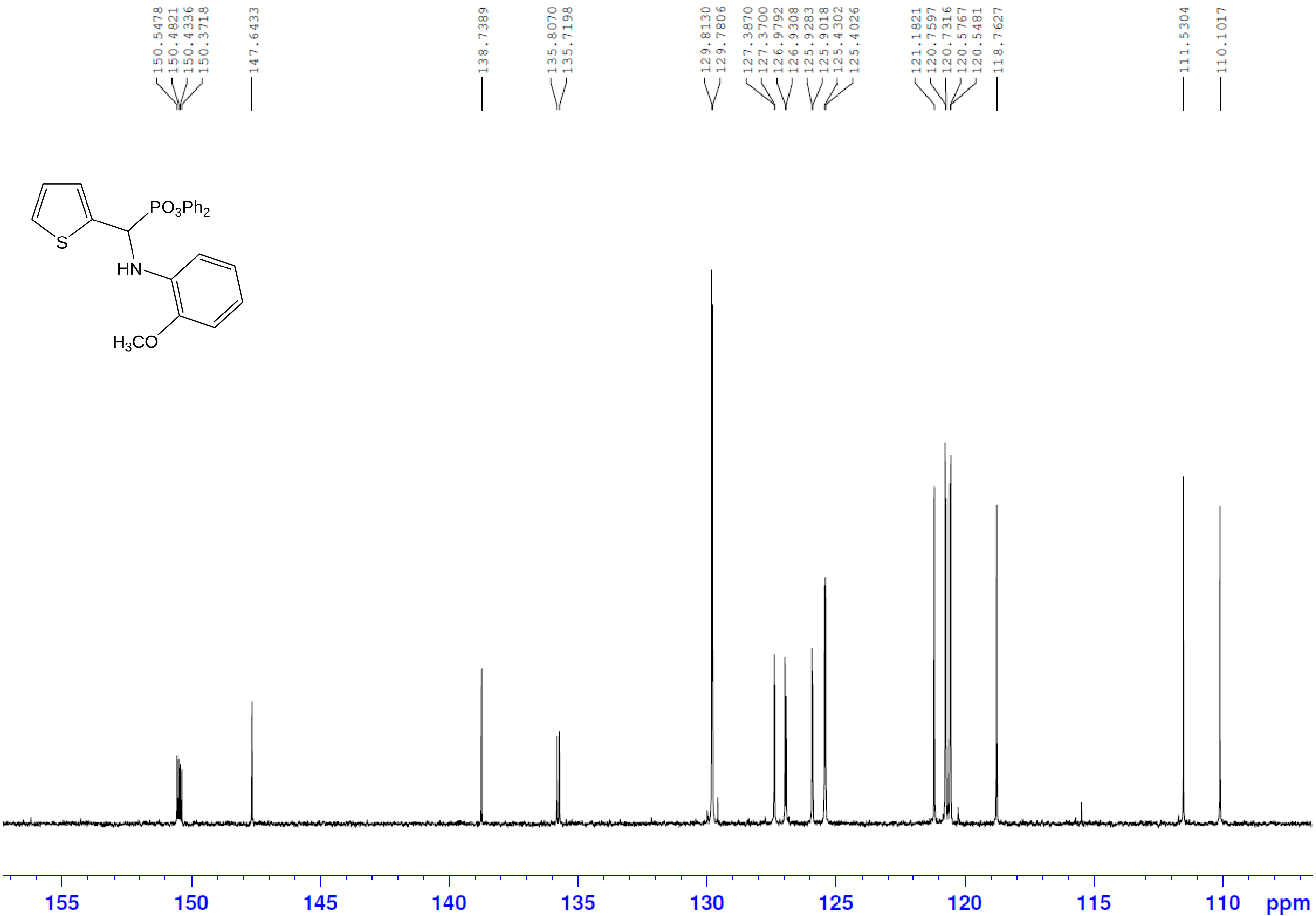
150.5478
150.4821
150.4336
150.3718
147.6433
138.7389
135.8070
135.7198
129.8130
129.7806
127.3870
127.3700
126.9792
126.9308
125.9283
125.9018
125.4302
125.4026
121.1821
120.7597
120.7316
120.5767
120.5481
118.7627
111.5304
110.1017
PO3Ph2
S
HN
H3CO
155
150
145
140
135
130
125
120
115
110
ppm

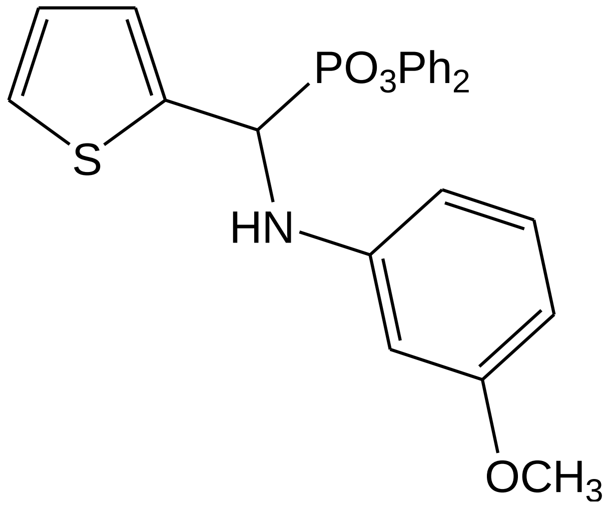

Fig. S5. ^{1}H NMR, ^{13}C NMR, ^{31}P NMR spectra of diphenyl N-(3-methoxyphenyl)amino(2-thienyl)methylphosphonate **(5)**

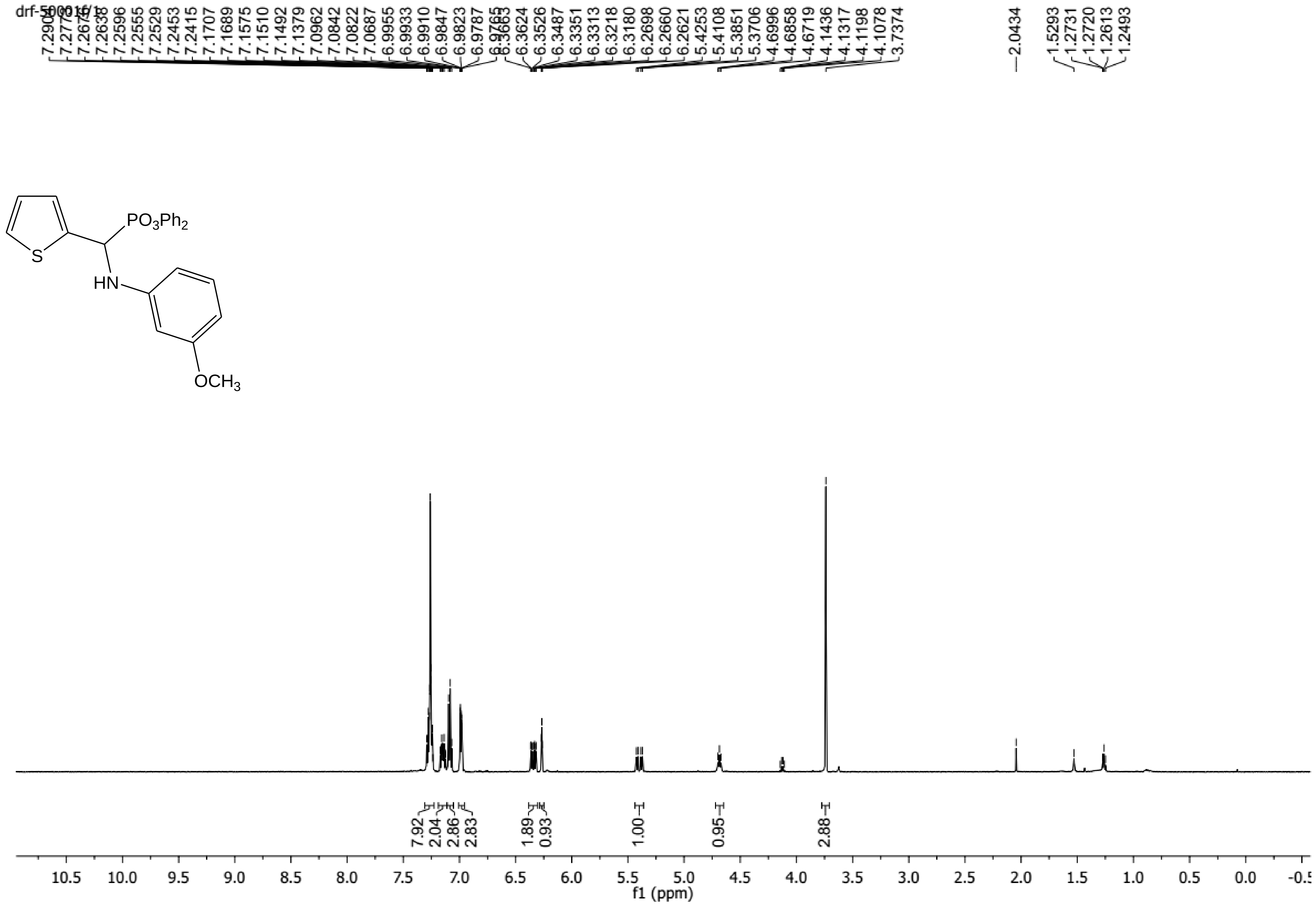
7.2638
7.2596
7.2555
7.2529
7.2453
7.2415
7.1707
7.1689
7.1575
7.1510
7.1492
7.1379
7.0962
7.0842
7.0822
7.0687
6.9955
6.9933
6.9910
6.9847
6.9823
6.9787
6.3624
6.3526
6.3487
6.3351
6.3313
6.3218
6.3180
6.2698
6.2660
6.2621
5.4253
5.4108
5.3851
5.3706
4.6996
4.6858
4.6719
4.1436
4.1317
4.1198
4.1078
3.7374
2.0434
1.5293
1.2731
1.2720
1.2613
1.2493
PO_3Ph_2
S
HN
OCH_3
7.92
2.04
2.86
2.83
1.89
0.93
1.00
0.95
2.88
10.5 10.0 9.5 9.0 8.5 8.0 7.5 7.0 6.5 6.0 5.5 5.0 4.5 4.0 3.5 3.0 2.5 2.0 1.5 1.0 0.5 0.0
f1 (ppm)

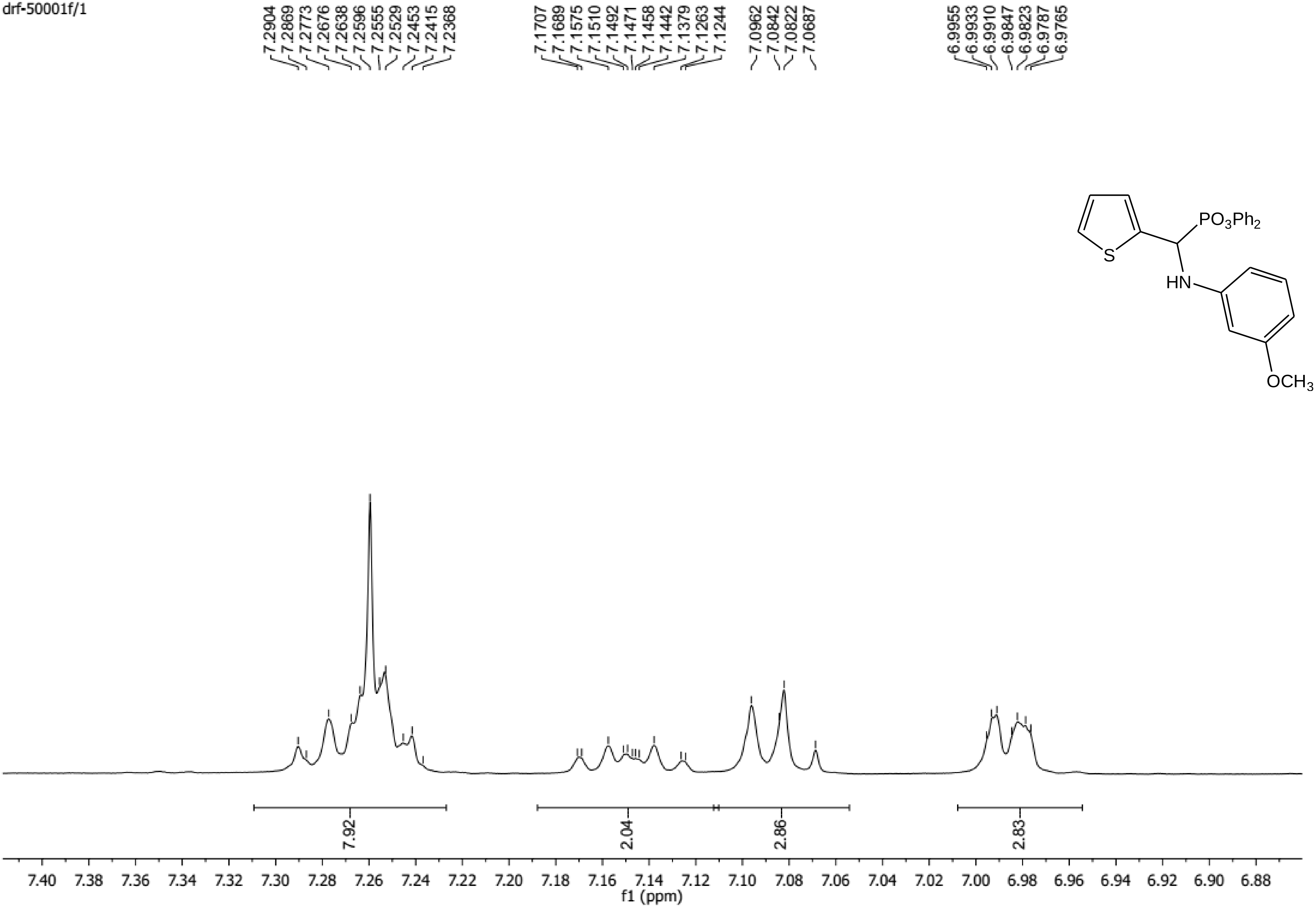

drf-50001f/1
7.2904
7.2869
7.2773
7.2676
7.2638
7.2596
7.2555
7.2529
7.2453
7.2415
7.2368
7.1707
7.1689
7.1575
7.1510
7.1492
7.1471
7.1458
7.1442
7.1379
7.1263
7.1244
7.0962
7.0842
7.0822
7.0687
6.9955
6.9933
6.9910
6.9847
6.9823
6.9787
6.9765
PO3Ph2
S
HN
OCH3
7.92
2.04
2.86
2.83
7.40 7.38 7.36 7.34 7.32 7.30 7.28 7.26 7.24 7.22 7.20 7.18 7.16 7.14 7.12 7.10 7.08 7.06 7.04 7.02 7.00 6.98 6.96 6.94 6.92 6.90 6.88
f1 (ppm)

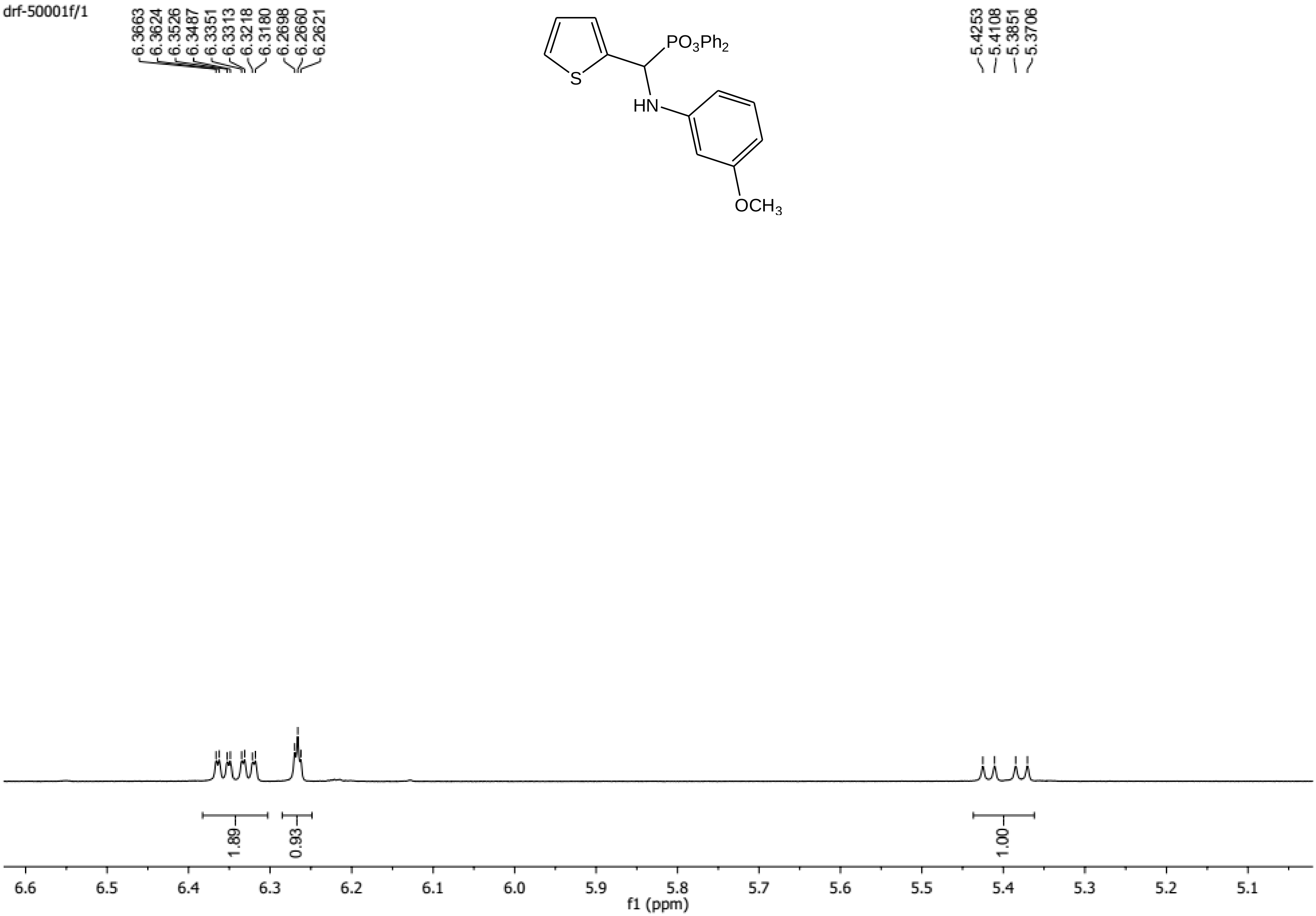

drf-50001f/1
6.3663
6.3624
6.3526
6.3487
6.3351
6.3313
6.3218
6.3180
6.2698
6.2660
6.2621
5.4253
5.4108
5.3851
5.3706
PO3Ph2
S
HN
OCH3
1.89
0.93
1.00
6.6
6.5
6.4
6.3
6.2
6.1
6.0
5.9
5.8
5.7
5.6
5.5
5.4
5.3
5.2
5.1
f1 (ppm)

drt-00005f/11

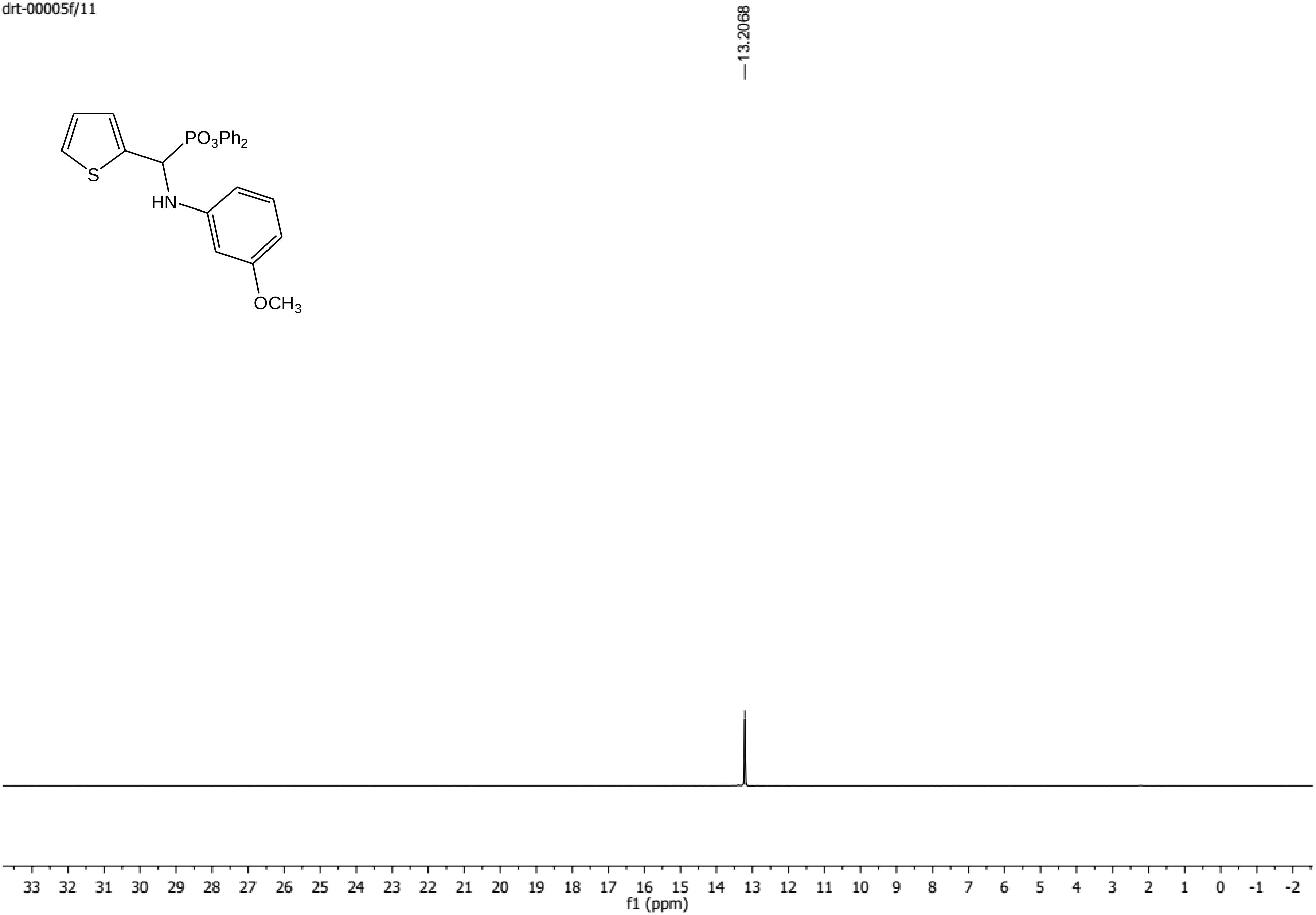

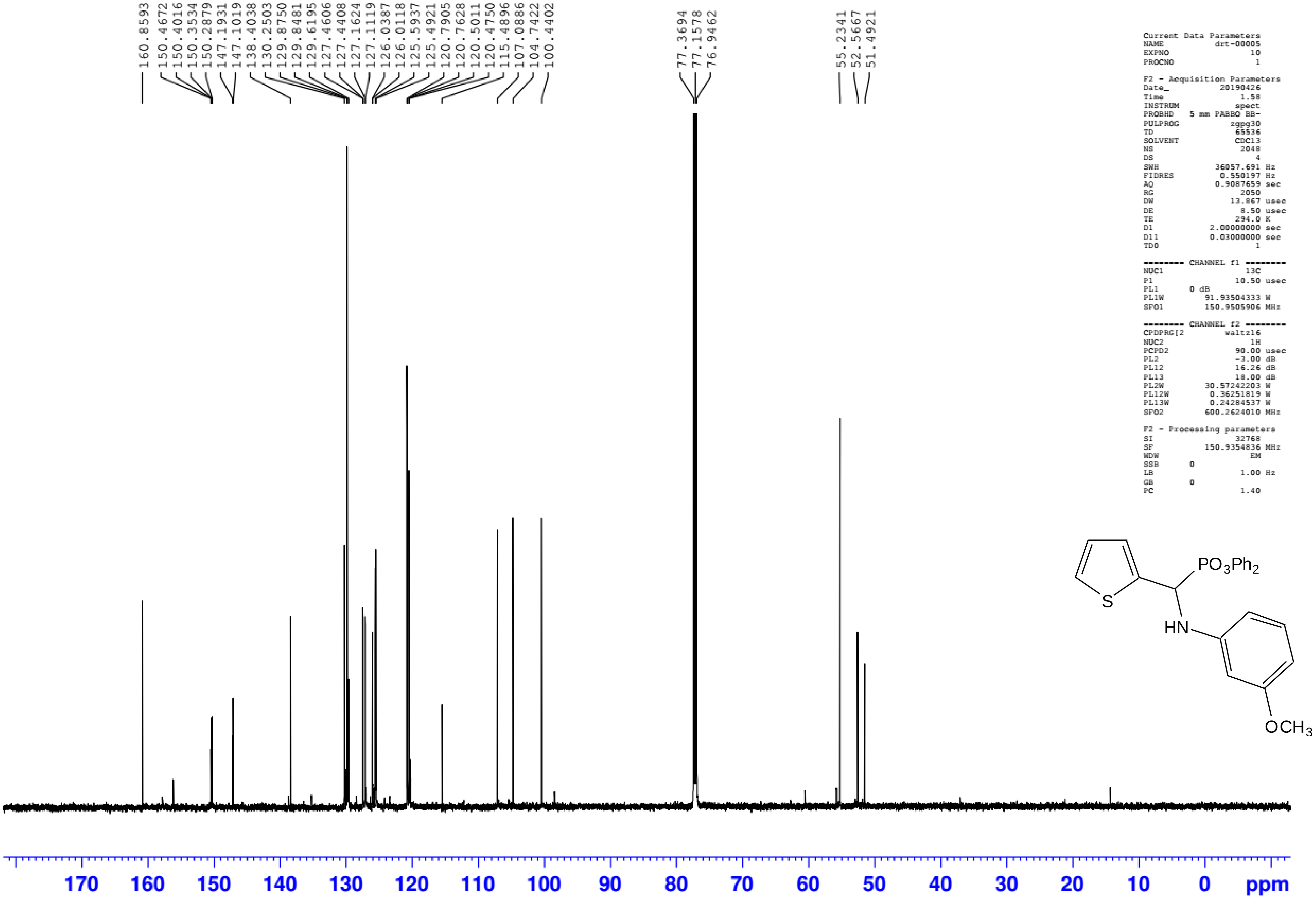

160.8593
150.4672
150.4016
150.3534
150.2879
147.1931
147.1019
138.4038
130.2503
129.8750
129.8481
129.6195
127.4606
127.4408
127.1624
127.1119
126.0387
126.0118
125.5937
125.4921
120.7905
120.7628
120.5011
120.4750
115.4896
107.0886
104.7422
100.4402
77.3694
77.1578
76.9462
55.2341
52.5667
51.4921
Current Data Parameters
NAME drt-00005
EXPNO 10
PROCNO 1
F2 - Acquisition Parameters
Date_ 20190426
Time 1.58
INSTRUM spect
PROBHD 5 mm PABBO BB-
PULPROG zgpg30
TD 65536
SOLVENT CDCl3
NS 2048
DS 4
SWH 36057.691 Hz
FIDRES 0.550197 Hz
AQ 0.9087659 sec
RG 2050
DW 13.867 usec
DE 8.50 usec
TE 294.0 K
D1 2.00000000 sec
D11 0.03000000 sec
TD0 1
======== CHANNEL f1 ========
NUC1 13C
P1 10.50 usec
PL1 0 dB
PL1W 91.93504333 W
SFO1 150.9505906 MHz
======== CHANNEL f2 ========
CPDPRG[2 waltz16
NUC2 1H
PCPD2 90.00 usec
PL2 -3.00 dB
PL12 16.26 dB
PL13 18.00 dB
PL2W 30.57242203 W
PL12W 0.36251819 W
PL13W 0.24284537 W
SFO2 600.2624010 MHz
F2 - Processing parameters
SI 32768
SF 150.9354836 MHz
WDW EM
SSB 0
LB 1.00 Hz
GB 0
PC 1.40
PO3Ph2
S
HN
OCH3
170 160 150 140 130 120 110 100 90 80 70 60 50 40 30 20 10 0 ppm

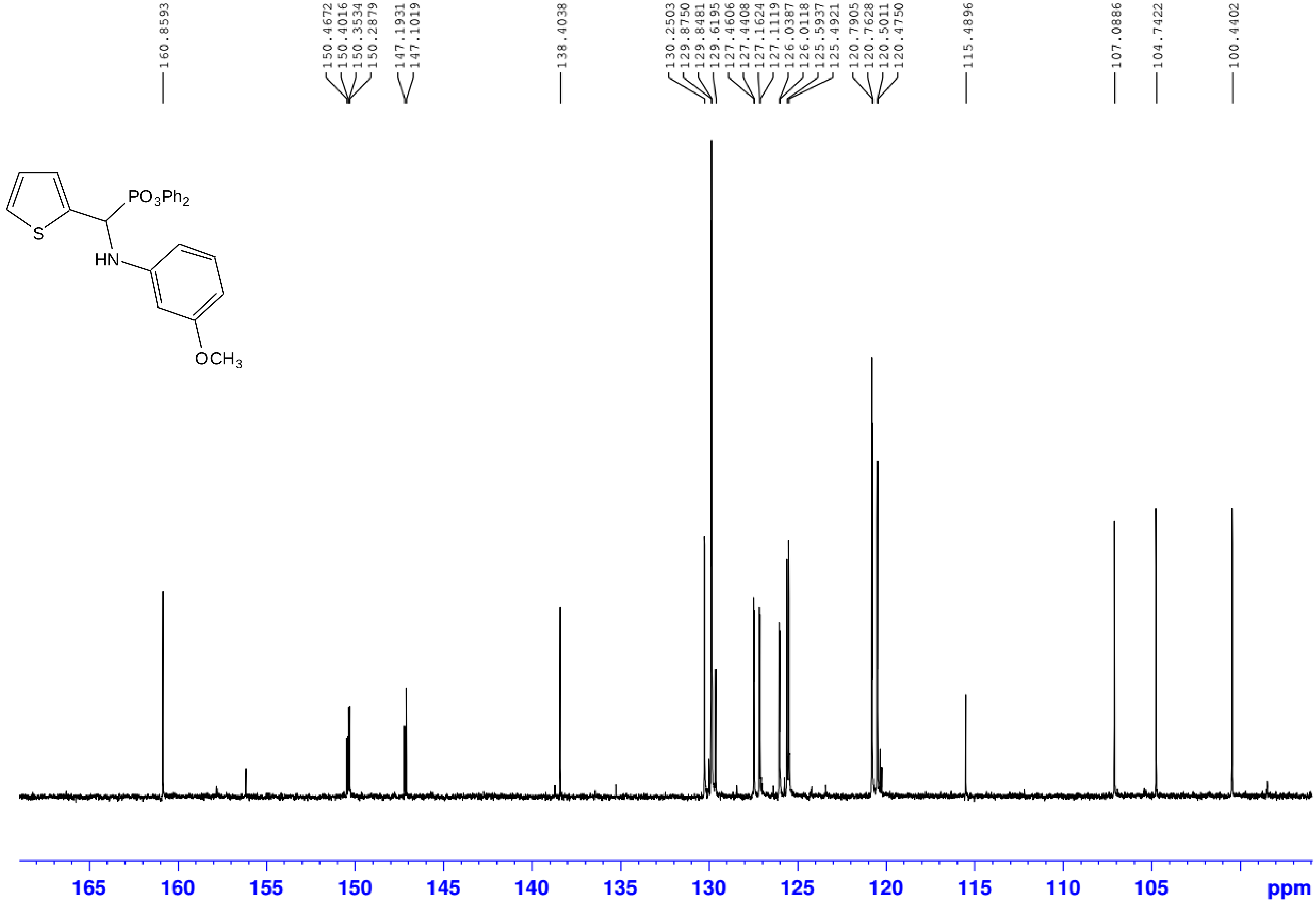

160.8593
150.4672
150.4016
150.3534
150.2879
147.1931
147.1019
138.4038
130.2503
129.8750
129.8481
129.6195
127.4606
127.4408
127.1624
127.1119
126.0387
126.0118
125.5937
125.4921
120.7905
120.7628
120.5011
120.4750
115.4896
107.0886
104.7422
100.4402
PO3Ph2
S
HN
OCH3
165
160
155
150
145
140
135
130
125
120
115
110
105
ppm

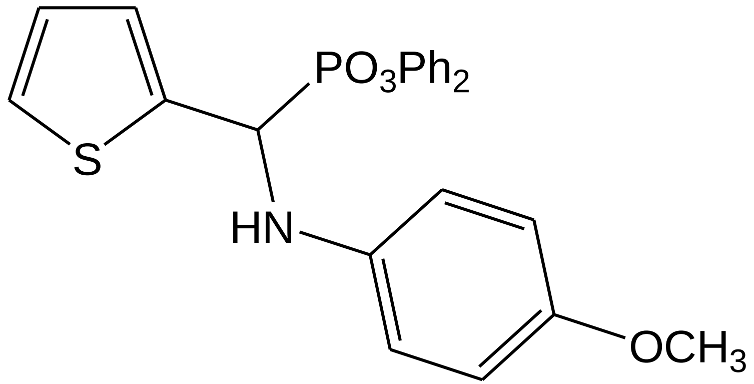

Fig. S6. ^{1}H NMR, ^{13}C NMR, ^{31}P NMR spectra of diphenyl N-(4-methoxyphenyl)amino(2-thienyl)methylphosphonate **(6)**

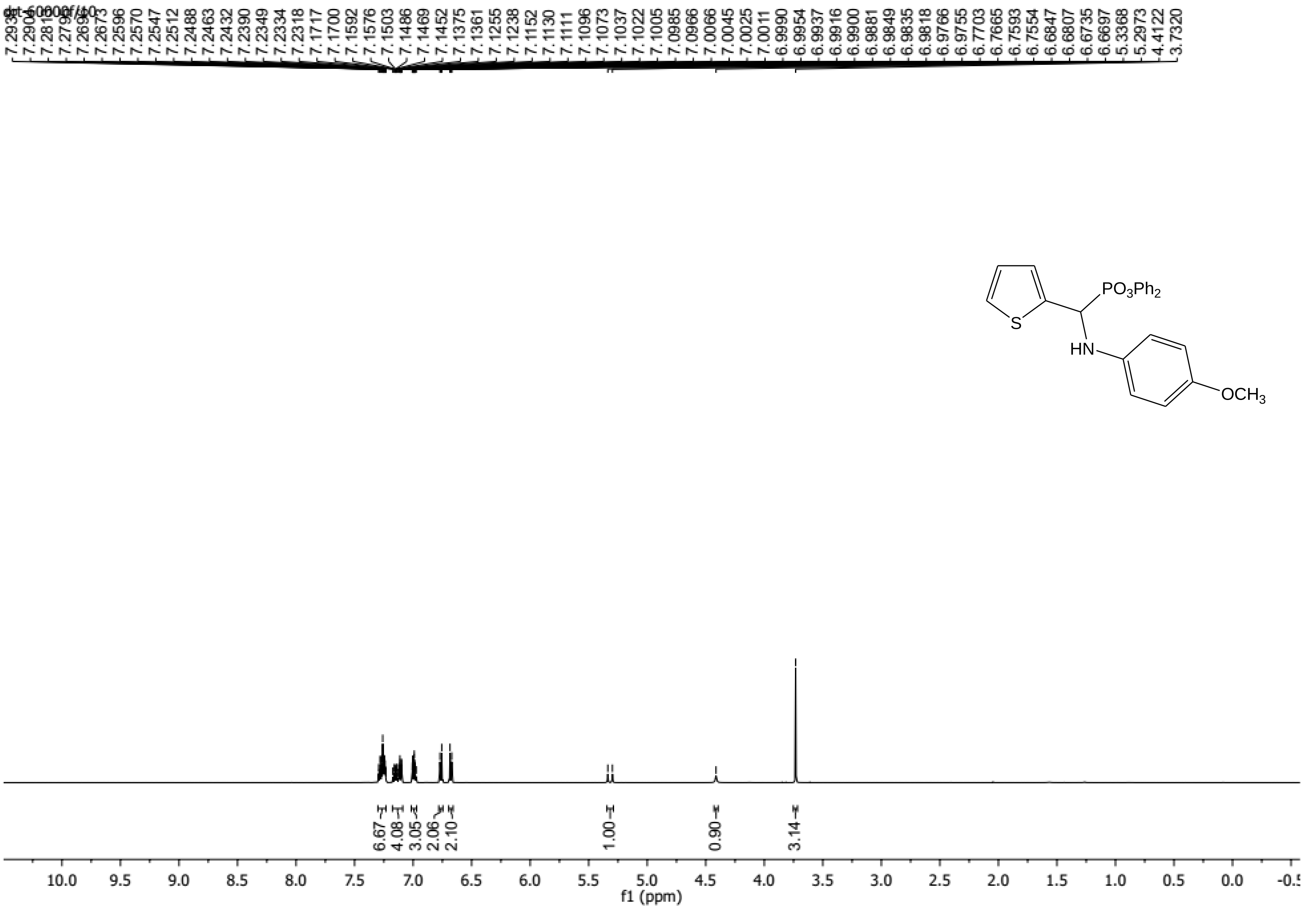
7.2938
7.2904
7.2819
7.2793
7.2693
7.2673
7.2596
7.2570
7.2547
7.2512
7.2488
7.2463
7.2432
7.2390
7.2349
7.2334
7.2318
7.1717
7.1700
7.1592
7.1576
7.1503
7.1486
7.1469
7.1452
7.1375
7.1361
7.1255
7.1238
7.1152
7.1130
7.1111
7.1096
7.1073
7.1037
7.1022
7.1005
7.0985
7.0966
7.0066
7.0045
7.0025
7.0011
6.9990
6.9954
6.9937
6.9916
6.9900
6.9881
6.9849
6.9835
6.9818
6.9766
6.9755
6.7703
6.7665
6.7593
6.7554
6.6847
6.6807
6.6735
6.6697
5.3368
5.2973
4.4122
3.7320
PO3Ph2
S
HN
OCH3
6.67
4.08
3.05
2.06
2.10
1.00
0.90
3.14
10.0
9.5
9.0
8.5
8.0
7.5
7.0
6.5
6.0
5.5
5.0
4.5
4.0
3.5
3.0
2.5
2.0
1.5
1.0
0.5
0.0
f1 (ppm)

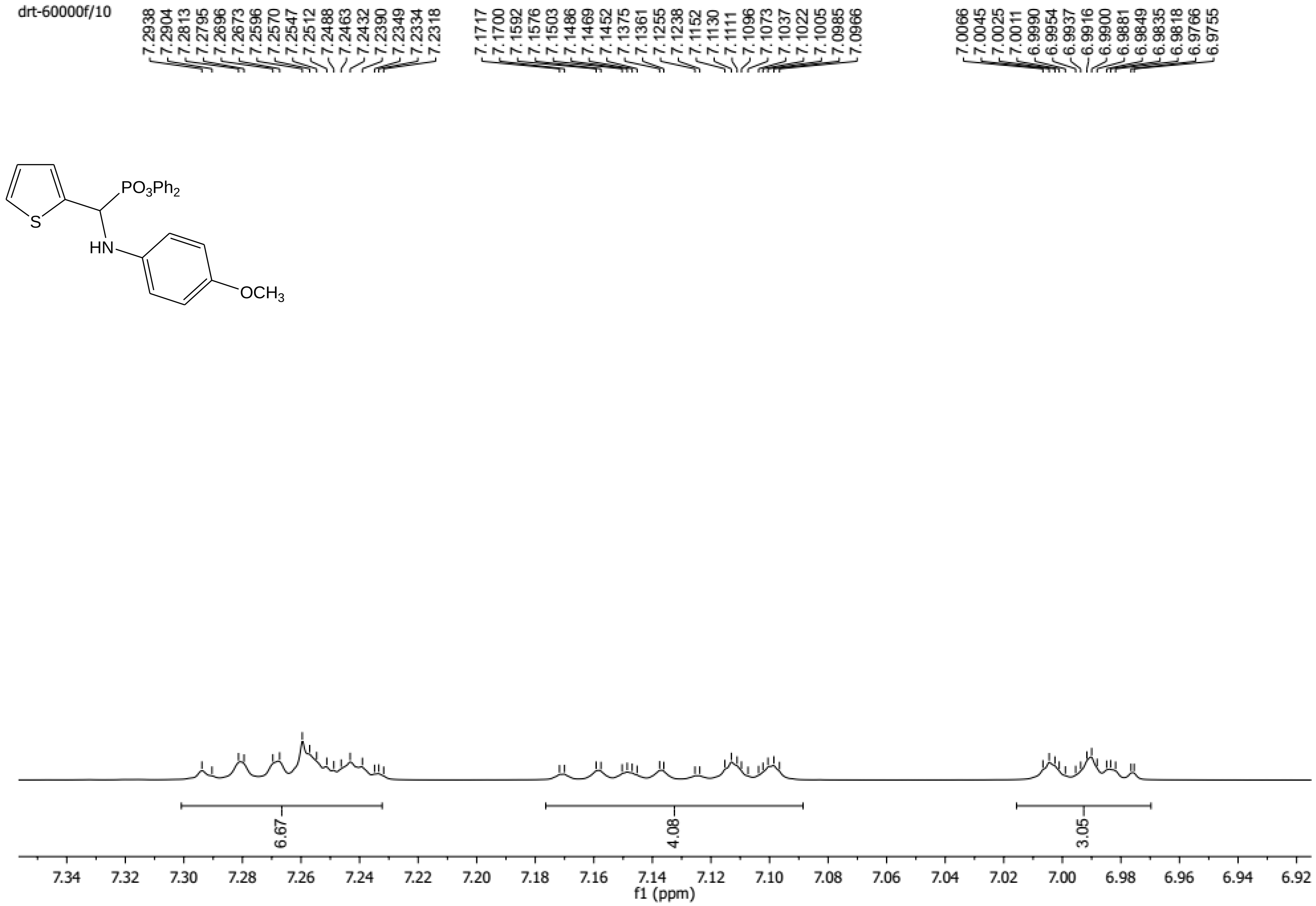
drt-60000f/10
7.2938
7.2904
7.2813
7.2795
7.2696
7.2673
7.2596
7.2570
7.2547
7.2512
7.2488
7.2463
7.2432
7.2390
7.2349
7.2334
7.2318
7.1717
7.1700
7.1592
7.1576
7.1503
7.1486
7.1469
7.1452
7.1375
7.1361
7.1255
7.1238
7.1152
7.1130
7.1111
7.1096
7.1073
7.1037
7.1022
7.1005
7.0985
7.0966
7.0066
7.0045
7.0025
7.0011
6.9990
6.9954
6.9937
6.9916
6.9900
6.9881
6.9849
6.9835
6.9818
6.9766
6.9755
PO3Ph2
S
HN
OCH3
6.67
4.08
3.05
7.34
7.32
7.30
7.28
7.26
7.24
7.22
7.20
7.18
7.16
7.14
7.12
7.10
7.08
7.06
7.04
7.02
7.00
6.98
6.96
6.94
6.92
f1 (ppm)

drt-60000f/10

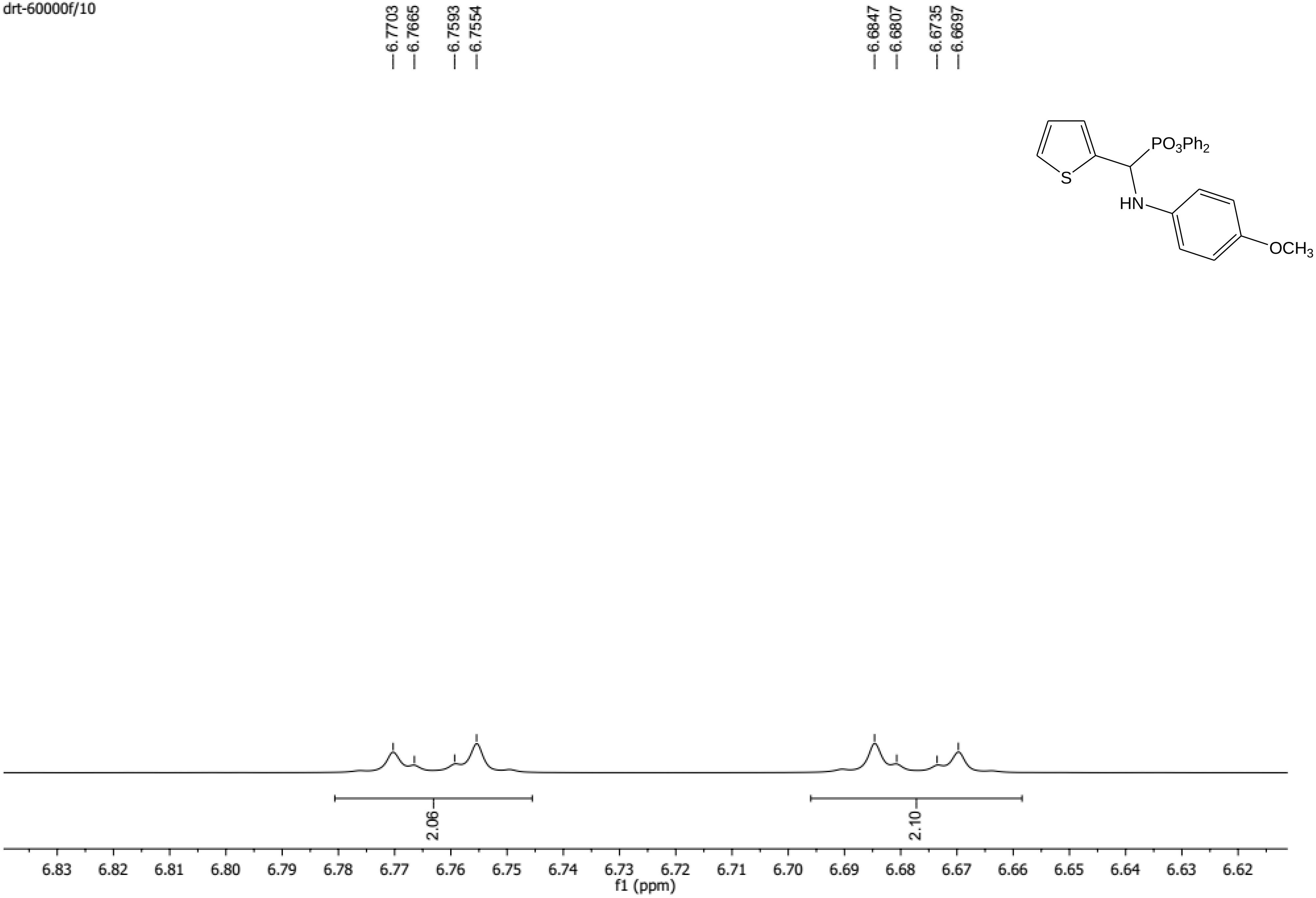

drt-60000f/10

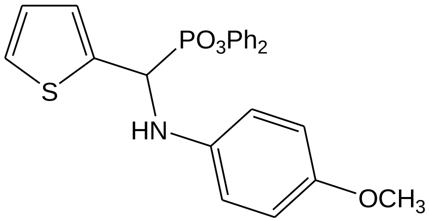

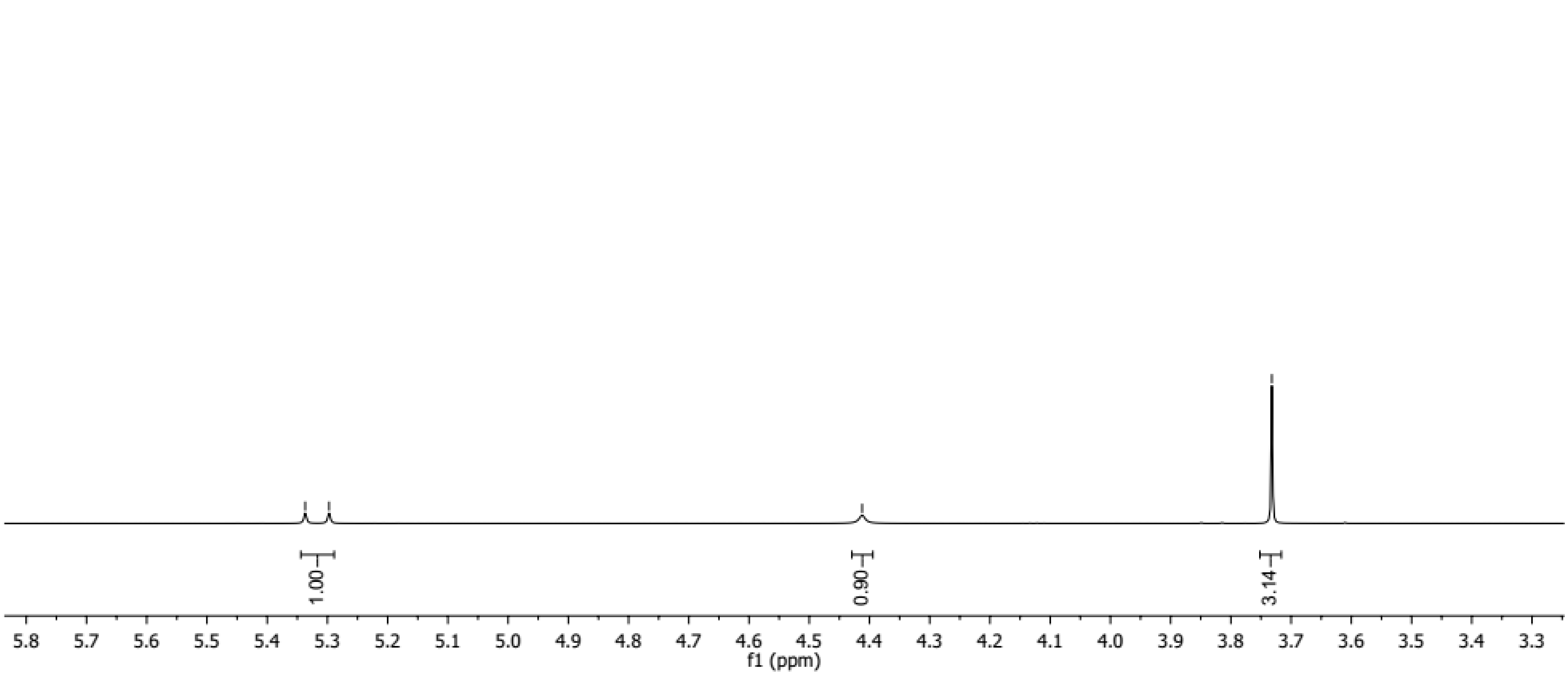

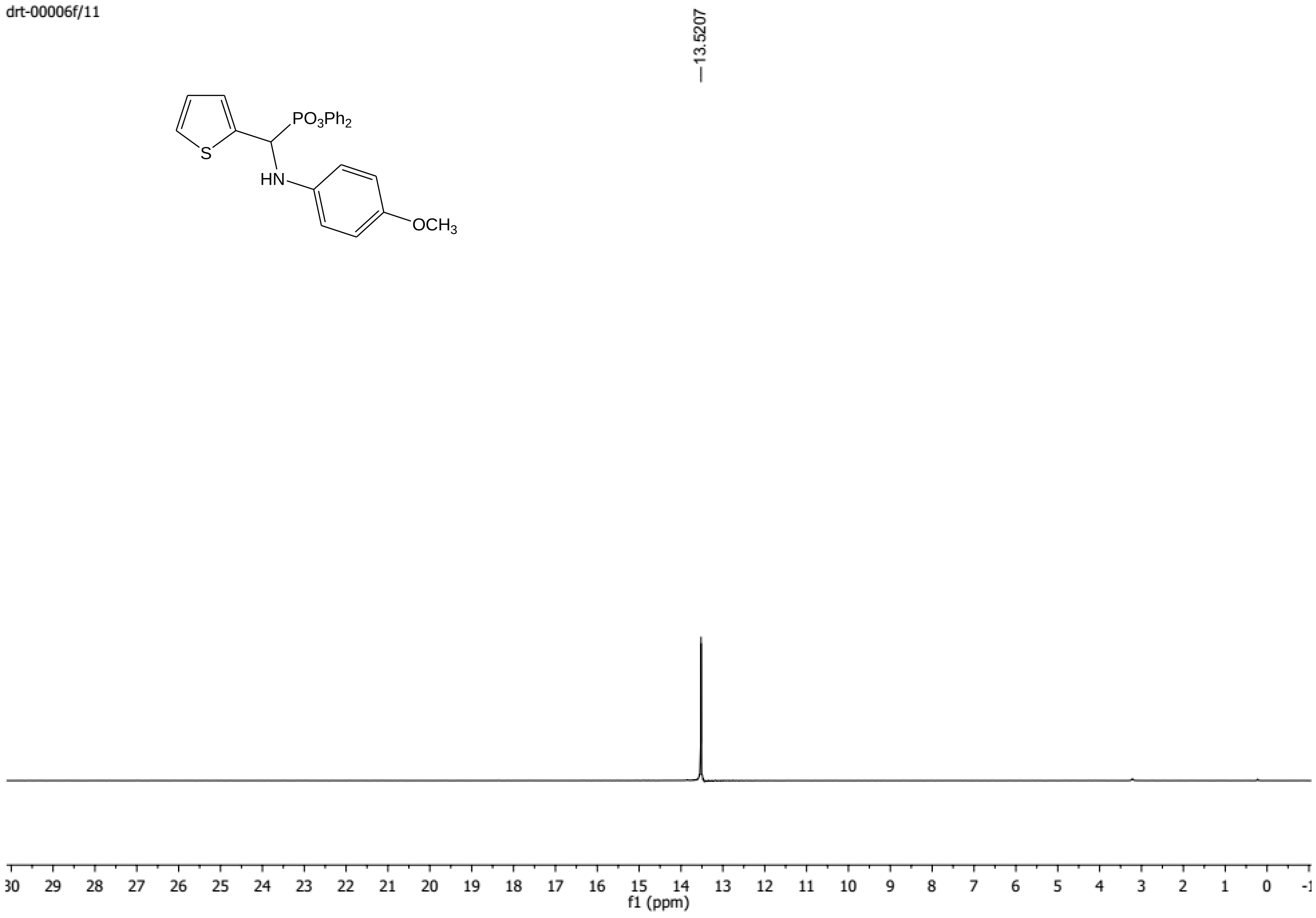
drt-00006f/11
13.5207
PO3Ph2
S
HN
OCH3
30 29 28 27 26 25 24 23 22 21 20 19 18 17 16 15 14 13 12 11 10 9 8 7 6 5 4 3 2 1 0 -1
f1 (ppm)

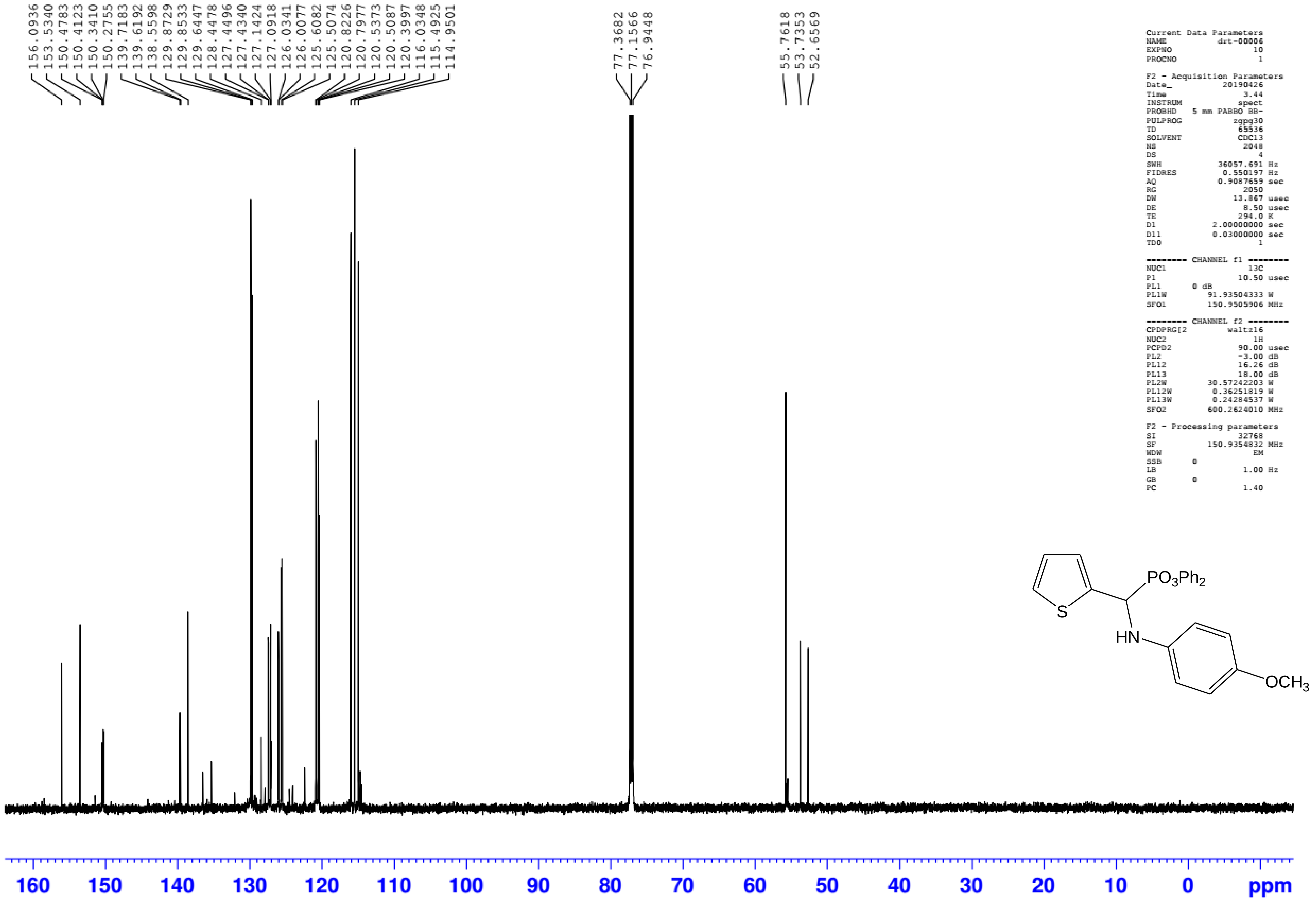
156.0936
153.5340
150.4783
150.4123
150.3410
150.2755
139.7183
139.6192
138.5598
129.8729
129.8533
129.6447
128.4478
127.4496
127.4340
127.1424
127.0918
126.0341
126.0077
125.6082
125.5074
120.8226
120.7977
120.5373
120.5087
120.3997
116.0348
115.4925
114.9501
77.3682
77.1566
76.9448
55.7618
53.7353
52.6569
Current Data Parameters
NAME drt-00006
EXPNO 10
PROCNO 1
F2 - Acquisition Parameters
Date_ 20190426
Time 3.44
INSTRUM spect
PROBHD 5 mm PABBO BB-
PULPROG zgpg30
TD 65536
SOLVENT CDCl3
NS 2048
DS 4
SWH 36057.691 Hz
FIDRES 0.550197 Hz
AQ 0.9087659 sec
RG 2050
DW 13.867 usec
DE 8.50 usec
TE 294.0 K
D1 2.00000000 sec
D11 0.03000000 sec
TD0 1
======== CHANNEL f1 ========
NUC1 13C
P1 10.50 usec
PL1 0 dB
PL1W 91.93504333 W
SFO1 150.9505906 MHz
======== CHANNEL f2 ========
CPDPRG[2 waltz16
NUC2 1H
PCPD2 90.00 usec
PL2 -3.00 dB
PL12 16.26 dB
PL13 18.00 dB
PL2W 30.57242203 W
PL12W 0.36251819 W
PL13W 0.24284537 W
SFO2 600.2624010 MHz
F2 - Processing parameters
SI 32768
SF 150.9354832 MHz
WDW EM
SSB 0
LB 1.00 Hz
GB 0
PC 1.40
PO3Ph2
S
HN
OCH3
160
150
140
130
120
110
100
90
80
70
60
50
40
30
20
10
0
ppm

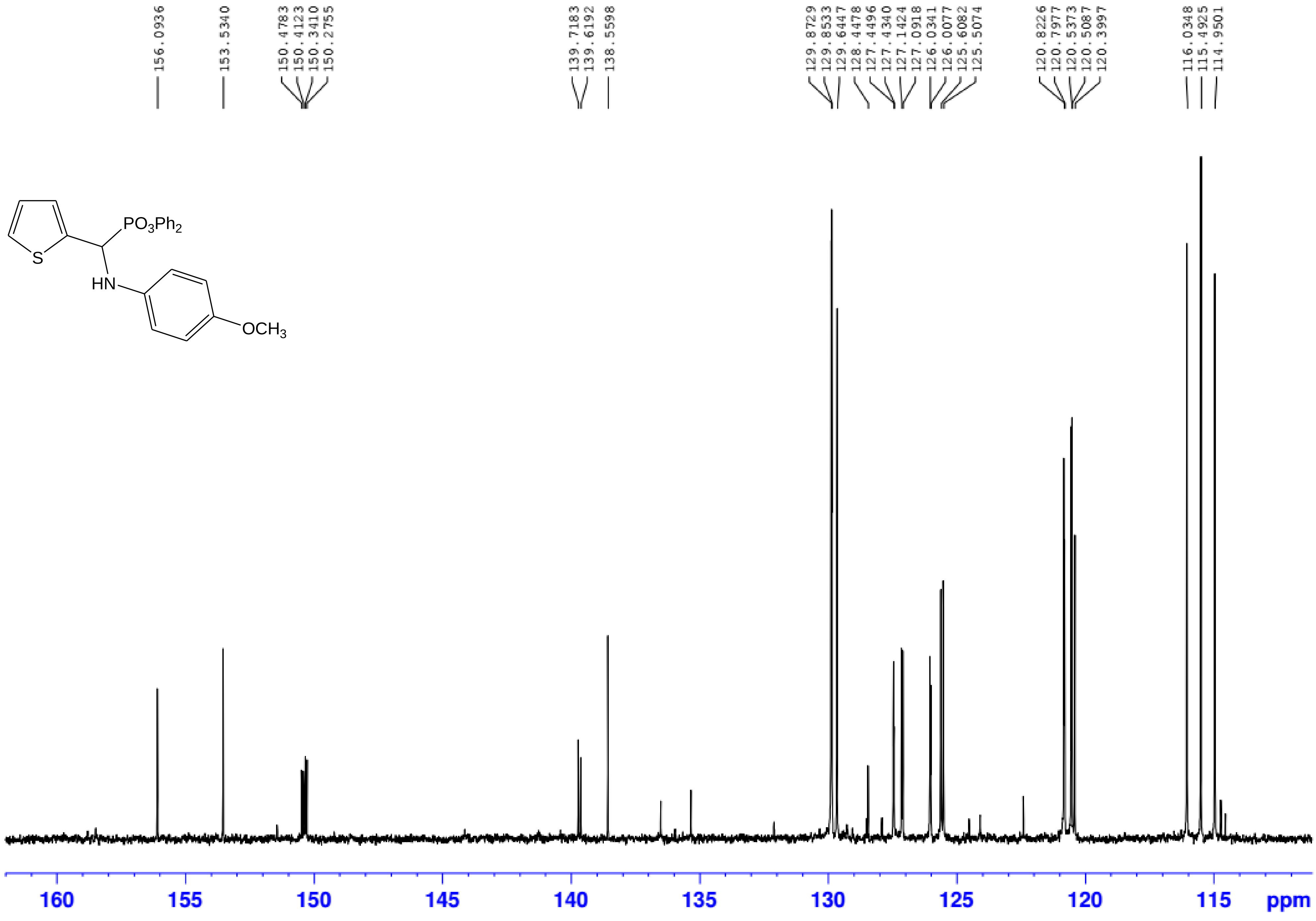
PO3Ph2
S
HN
OCH3
156.0936
153.5340
150.4783
150.4123
150.3410
150.2755
139.7183
139.6192
138.5598
129.8729
129.8533
129.6447
128.4478
127.4496
127.4340
127.1424
127.0918
126.0341
126.0077
125.6082
125.5074
120.8226
120.7977
120.5373
120.5087
120.3997
116.0348
115.4925
114.9501
160
155
150
145
140
135
130
125
120
115
ppm

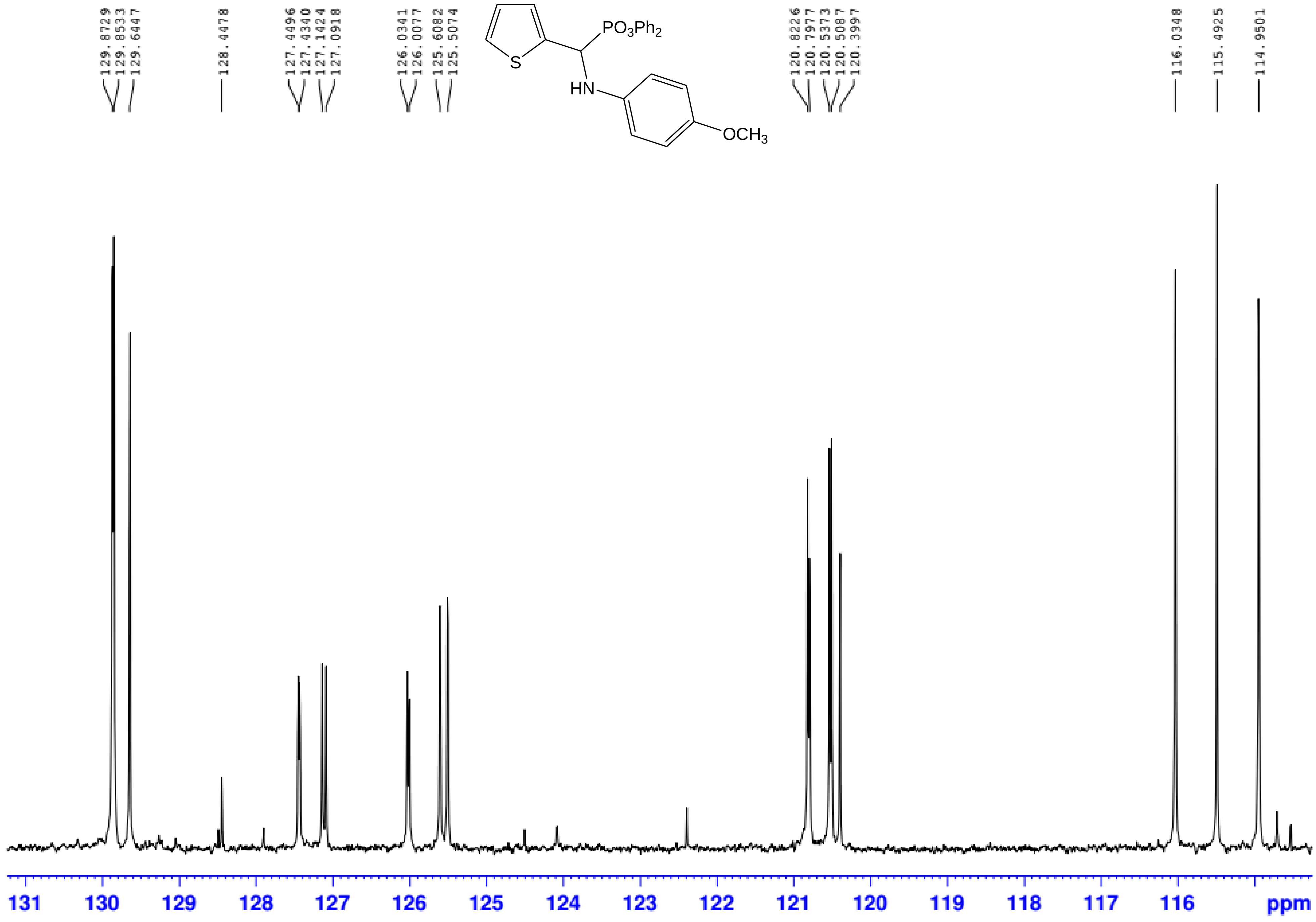
129.8729
129.8533
129.6447
128.4478
127.4496
127.4340
127.1424
127.0918
126.0341
126.0077
125.6082
125.5074
PO3Ph2
S
HN
OCH3
120.8226
120.7977
120.5373
120.5087
120.3997
116.0348
115.4925
114.9501
131
130
129
128
127
126
125
124
123
122
121
120
119
118
117
116
ppm
