# Supplementary material for: The Effect of New Thiophene-Derived Diphenyl Aminophosphonates on Growth of Terrestrial Plants
Source: Materials (Basel). 2019 Jun 24;12(12):2018. doi: 10.3390/ma12122018 (PMC6630915; doi:10.3390/ma12122018)
Supplement: Supplementary file 1 [file materials-12-02018-s001.zip › materials-519639 supplemenatry final check/materials-519639-supplementary 2.pdf]

*materials*

MDPI

*Article*

# The Effect of New Thiophene-Derived DiphenylAminophosphonates on Growth of Terrestrial Plants

**Diana Rogacz [1], Jarosław Lewkowski [2,*], Marta Siedlarek [2],Rafał Karpowicz [2], Anna Kowalczyk [2]and Piotr Rychter[1,*]**

1 Faculty of Mathematics and Natural Science, Jan Długosz University in Częstochowa, 42-200 Częstochowa, 13/15 Armii Krajowej Av., Poland; diana.rogacz@gmail.com (D.R.)

2 Department of Organic Chemistry, Faculty of Chemistry, University of Łódź, Tamka 12, 91-403 Łódź, Poland; mz.morawska@gmail.com (M.S.); rafal.karpowicz@chemia.uni.lodz.pl (R.K.); anak56@gmail.com (A.K.)

* Correspondence:jaroslaw.lewkowski@chemia.uni.lodz.pl (J.L.); p.rychter@ajd.czest.pl (P.R.); Tel.: +48-42-635-5751 (J.L.); +48-34-361-5154 (P.R.)

## Supplementary Materials part 2

## 2. Materials and Methods

### *2.1. Preparation of Aminophosphonates 1-6*

#### 2.1.1. General Information

All solvents (POCh, Gliwice, Poland) were routinely distilled and dried prior to use. Amines, diphenylphosphite, and thiophene-2-carboxaldehyde (Aldrich, Poznań, Poland) were used as received.

Melting points were measured on a MelTemp II apparatus (Bibby Scientific Limited, Staffordshire, UK) and were not corrected. NMR spectra were recorded on an Avance III 600 MHz apparatus (Bruker, Billerica, MA, USA) operating at 600 MHz ($^{1}$H NMR), 150 MHz ($^{13}$C NMR), and 243 MHz ($^{31}$P NMR). Elemental analyses were carried out in the Laboratory of Microanalysis, Faculty of Chemistry, University of Ł ódź, Poland.

#### 2.1.2. Procedure for Preparation of DiphenylAmino(2-thienyl)methylphosphonates1-6

The procedure for synthesis of **1-6** was similar to the previously published method [1,2]: Thiophene-2-carboxaldehyde (12 mmol, 1.34 g) was dissolved in methanol (15 mL) and a solution of an appropriate toluidine or anisidine (12 mmol) in methanol (15 mL) was added. The mixture was stirred magnetically for 24 h at room temperature. Then, 2–3 g of anhydrous potassium carbonate was added and after several minutes the mixture was filtrated and evaporated. Identities of obtained imines was confirmed by 1H NMR and they were used for further reactions without any purification.

Obtained imine (10 mmol) was dissolved in acetonitrile (15 mL) and a solution of diphenylphosphite (10 mmol, 2.34 g, 1.91 mL) in acetonitrile (15 mL) was added. The obtained mixture was refluxed (at 80 C) during a day and stirred at room temperature at night. The reaction lasted for 72 h. Then the solvent was evaporated in vacuo. Crude products were purified by column chromatography on silica gel eluted with ethyl acetate:hexane 3:1.

*Diphenyl N-(2-methylphenyl)amino(2-thienyl)methylphosphonate (1).*

Yield = 71% (3.10 g) ecru crystals, mp: = 62–64 °C. $^{1}$H NMR (600 MHz, $CDCl_3$): δ 7.29–7.26 (m, 6H, ArH); 7.18–7.14 (m, 2H, $C_6H_4$); 7.10–7.07 (m, 4H, ArH); 7.02–7.00 (m, 3H, ArH); 6.75 (approx. t,

$^3J_{HH}$ = 7.4 Hz, 1H, $C_6H_4$); 6.68 (d, $^3J_{HH}$ = 7.9 Hz, 1H, $C_6H_4$); 5.48 (d, $^2J_{PH}$ = 24.1 Hz, 1H, CHP); 4.58 (br. S, 1H, NH); 2.19 (s, 3H, $CH_3$). $^{31}$P NMR (243 MHz, $CDCl_3$): δ 13.33. $^{13}$C NMR (150 MHz, $CDCl_3$): δ 150.6 (d, $^2J_{CP}$ = 9.5 Hz, $POC_{Ar}$); 150.4 (d, $^2J_{CP}$ = 9.7 Hz, $POC_{Ar}$); 143.8 (d, $^2J_{CP}$ = 13.0 Hz, $PCC_{thioph}$); 138.8 ($C_{Ar}$); 130.6 ($C_{Ar}$); 129.8 ($C_{Ar}$); 127.4 (d, $^5J_{CP}$ = 2.9 Hz, $C_{5thioph}$); 127.2 ($C_{Ar}$); 126.9 (d, $^3J_{CP}$ = 7.3 Hz, $C_{3thioph}$); 125.9 (d, $^4J_{CP}$ = 4.1 Hz, $C_{thioph}$); 125.5 ($C_{Ar}$); 125.4 ($C_{Ar}$); 123.6 ($C_{Ar}$); 120.7 (d, $^3J_{CP}$ = 4.1 Hz, $C_{Ar}$); 120.5 (d, $^3J_{CP}$ = 4.3 Hz, $C_{Ar}$); 119.2 ($C_{Ar}$); 111.6 ($C_{Ar}$); 52.2 (d, $^1J_{CP}$ = 159.9 Hz, CP); 17.5 (Ar-$CH_3$). Elem. Anal. Calculated for $C_{24}H_{22}NO_3PS$: C, 66.19; H, 5.09; N, 3.22; S, 7.36. Found: C, 66.01; H, 5.23; N, 3.42; S, 7.23.

*Diphenyl N-(3-methylphenyl)amino(2-thienyl)methylphosphonate (2)*

Yield = 94% (4.10 g) ecru crystals, mp: = 124–126 °C. $^1$H NMR (600 MHz, $CDCl_3$): δ 7.29–7.24 (m, 6H, ArH); 7.17–7.14 (m, 2H, ArH); 7.09–7.05 (m, 3H, ArH); 7.00–6.98 (m, 3H, ArH); 6.62 (d, $^3J_{HH}$ = 7.4 Hz, 1H, $C_6H_4$); 6.55-6.54 (m, 1H, $C_6H_4$); 6.52 (dd, $^3J_{HH}$ = 7.9 and $^4J_{HH}$ = 2.3 Hz, 1H, $C_6H_4$); 5.42 (d, $^2J_{PH}$ = 24.1 Hz, 1H, CHP); 4.61 (broad s, 1H, NH); 2.26 (s, 3H, $CH_3$). $^{31}$P NMR (243 MHz, $CDCl_3$): δ 13.33. $^{13}$C NMR (150 MHz, $CDCl_3$): δ 150.4 (d, $^2J_{CP}$ = 9.8 Hz, $POC_{Ar}$); 150.3 (d, $^2J_{CP}$ = 9.8 Hz, $POC_{Ar}$); 145.7 (d, $^2J_{CP}$ = 13.2 Hz, $PCC_{thioph}$); 139.3 ($C_{Ar}$); 138.6 ($C_{Ar}$); 129.8 (d, $^2J_{CP}$ = 2.3 Hz, $C_{Ar}$); 129.3 ($C_{Ar}$); 127.4 (d, $^5J_{CP}$ = 2.9 Hz, $C_{5thioph}$); 127.1 (d, $^3J_{CP}$ = 7.1 Hz, $C_{3thioph}$); 125.9 (d, $^4J_{CP}$ = 3.9 Hz, $C_{thioph}$); 125.5 ($C_{Ar}$); 125.4 ($C_{Ar}$); 120.8 (d, $^3J_{CP}$ = 4.3 Hz, $C_{Ar}$); 120.5 (d, $^3J_{CP}$ = 4.4 Hz, $C_{Ar}$); 120.4 ($C_{Ar}$); 115.2 ($C_{Ar}$); 111.3 ($C_{Ar}$); 52.1 (d, $^1J_{CP}$ = 161.5 Hz, CP); 21.7 (Ar-$CH_3$). Elem. Anal. Calculated for $C_{24}H_{22}NO_3PS$: C, 66.19; H, 5.09; N, 3.22; S, 7.36. Found: C, 66.30; H, 5.24; N, 3.42; S, 7.15.

*Diphenyl N-(4-methylphenyl)amino(2-thienyl)methylphosphonate (3)*

Yield = 80% (3.50 g) ecru crystals, mp: = 149–151 °C. $^1$H NMR (600 MHz, $CDCl_3$): δ 7.29–7.24 (m, 6H, ArH); 7.17–7.12 (m, 2H, ArH); 7.10–7.09 (m, 2H, ArH); 7.00–6.97 (m, 5H, ArH); 6.65–6.62 (m, 2H, ArH); 5.38 (dd, $^2J_{PH}$ = 24.0 and $^3J_{HH}$ = 8.1 Hz, 1H, CHP); 4.54 (dd, $^3J_{PH}$ = $^3J_{HH}$ = 8.0 Hz, 1H, NH); 2.24 (s, 3H, $CH_3$). $^{31}$P NMR (243 MHz, $CDCl_3$): δ 13.43. $^{13}$C NMR (150 MHz, $CDCl_3$): δ 150.5 (d, $^2J_{CP}$ = 9.8 Hz, $POC_{Ar}$); 150.3 (d, $^2J_{CP}$ = 9.7 Hz, $POC_{Ar}$); 143.4 (d, $^2J_{CP}$ = 13.8 Hz, $PCC_{thioph}$); 138.7 ($C_{Ar}$); 130.0 ($C_{Ar}$); 129.8 (d, $^4J_{CP}$ = 3.1 Hz, $C_{Ar}$); 128.8 ($C_{Ar}$); 127.4 (d, $^5J_{CP}$ = 2.7 Hz, $C_{5thioph}$); 127.0 (d, $^3J_{CP}$ = 7.6 Hz, $C_{3thioph}$); 125.9 (d, $^4J_{CP}$ = 4.1 Hz, $C_{thioph}$); 125.5 ($C_{Ar}$); 125.4 ($C_{Ar}$); 120.8 (d, $^3J_{CP}$ = 4.2 Hz, $C_{Ar}$); 120.5 (d, $^3J_{CP}$ = 3.9 Hz, $C_{Ar}$); 114.5 ($C_{Ar}$); 52.4 (d, $^1J_{CP}$ = 161.5 Hz, CP); 20.6 (Ar-$CH_3$). Elem. Anal. Calculated for $C_{24}H_{22}NO_3PS$: C, 66.19; H, 5.09; N, 3.22; S, 7.36. Found: C, 66.33; H, 5.16; N, 3.37; S, 7.33.

*Diphenyl N-(2-methoxyphenyl)amino(2-thienyl)methylphosphonate (4)*

Yield = 89% (4.00 g) ecru crystals, mp: = 91–93 °C. $^1$H NMR (600 MHz, $CDCl_3$): δ 7.28–7.24 (m, 6H, ArH); 7.16–7.13 (m, 2H, ArH); 7.09–7.08 (m, 2H, ArH); 7.04–7.02 (m, 2H, ArH); 6.99–6.98 (m, 1H, ArH); 6.83–6.80 (m, 2H, ArH); 6.76–6.74 (m, 1H, ArH); 6.67–6.66 (m, 1H, ArH); 5.41 (d, $^2J_{PH}$ = 23.7 Hz, 1H, CHP); 3.86 (s, 3H, $OCH_3$). $^{31}$P NMR (243 MHz, $CDCl_3$): δ 13.37. $^{13}$C NMR (150 MHz, $CDCl_3$): δ 150.5 (d, $^2J_{CP}$ = 9.9 Hz, $POC_{Ar}$); 150.4 (d, $^2J_{CP}$ = 9.3 Hz, $POC_{Ar}$); 147.6 ($C_{Ar}$); 138.7 ($C_{Ar}$); 135.8 (d, $^2J_{CP}$ = 13.1 Hz, $PCC_{thioph}$); 129.8 (d, $^4J_{CP}$ = 4.9 Hz, $C_{Ar}$); 127.4 (d, $^5J_{CP}$ = 2.6 Hz, $C_{5thioph}$); 127.0 (d, $^3J_{CP}$ = 7.3 Hz, $C_{3thioph}$); 125.9 (d, $^4J_{CP}$ = 4.0 Hz, $C_{thioph}$); 125.4 (d, $^4J_{CP}$ = 4.1 Hz, $C_{Ar}$); 121.2 ($C_{Ar}$); 120.7 (d, $^3J_{CP}$ = 4.2 Hz, $C_{Ar}$); 120.6 (d, $^3J_{CP}$ = 4.3 Hz, $C_{Ar}$); 118.8 ($C_{Ar}$); 111.5 ($C_{Ar}$); 110.1 ($C_{Ar}$); 55.7 (Ar-$OCH_3$); 52.0 (d, $^1J_{CP}$ = 160.6 Hz, CP). Calculated for $C_{24}H_{22}NO_4PS$: C, 63.85; H, 4.91; N, 3.10; S, 7.10. Found: C, 63.63; H, 4.97; N, 3.25; S, 7.07.

*Diphenyl N-(3-methoxyphenyl)amino(2-thienyl)methylphosphonate (5)*

Yield = 75% (3.40 g) ecru crystals, mp: = 95–97 °C. $^1$H NMR (600 MHz, $CDCl_3$): δ 7.29–7.24 (m, 6H, ArH); 7.17–7.12 (m, 2H, ArH); 7.10–7.07 (m, 3H, ArH); 7.00–6.98 (m, 3H, ArH); 6.36 (dd, $^3J_{HH}$ = 8.2 and $^4J_{HH}$ = 2.3 Hz, 1H, $C_6H_4$); 6.33 (dd, $^3J_{HH}$ = 8.0 and $^4J_{HH}$ = 2.3 Hz, 1H, $C_6H_4$); 6.27–6.26 (m, 1H, ArH); 5.40 (dd, $^2J_{PH}$ = 24.1 and $^3J_{HH}$ = 8.7 Hz, 1H, CHP); 4.69 (dd, $^3J_{PH}$ = $^3J_{HH}$ = 8.3 Hz, 1H, NH); 3.74 (s, 3H, $OCH_3$). $^{31}$P NMR (243 MHz, $CDCl_3$): δ 13.21. $^{13}$C NMR (150 MHz, $CDCl_3$): δ 160.9 ($C_{Ar}$); 150.4 (d, $^2J_{CP}$ = 9.8 Hz, $POC_{Ar}$); 150.3 (d, $^2J_{CP}$ = 9.8 Hz, $POC_{Ar}$); 147.2 (d, $^2J_{CP}$ = 13.7 Hz, $PCC_{thioph}$); 138.4 ($C_{Ar}$); 130.3 ($C_{Ar}$); 129.9 (d, $^4J_{CP}$ = 4.0 Hz, $C_{Ar}$); 129.6 ($C_{Ar}$); 127.5 (d, $^5J_{CP}$ = 3.0 Hz, $C_{5thioph}$); 127.1 (d, $^3J_{CP}$ = 7.6 Hz, $C_{3thioph}$);

126.0 (d, $^4J_{CP}$ = 4.0 Hz, $C_{thioph}$); 125.6 ($C_{Ar}$); 125.5 ($C_{Ar}$); 121.2 ($C_{Ar}$); 120.8 (d, $^3J_{CP}$ = 4.2 Hz, $C_{Ar}$); 120.5 (d, $^3J_{CP}$ = 3.9 Hz, $C_{Ar}$); 115.5 ($C_{Ar}$); 107.1 ($C_{Ar}$); 104.7 ($C_{Ar}$); 100.4 ($C_{Ar}$); 55.2 (Ar-$OCH_3$); 52.0 (d, $^1J_{CP}$ = 161.2 Hz, CP). Calculated for $C_{24}H_{22}NO_4PS$: C, 63.85; H, 4.91; N, 3.10; S, 7.10. Found: C, 63.93; H, 4.97; N, 3.27; S, 7.04.

*Diphenyl N-(4-methoxyphenyl)amino(2-thienyl)methylphosphonate (6)*

Yield = 74% (3.35 g) ecru crystals, mp: = 87–91 °C. $^1$H NMR (600 MHz, $CDCl_3$): δ 7.29–7.23 (m, 6H, ArH); 7.17–7.10 (m, 4H, ArH); 7.00–6.98 (m, 3H, ArH); 6.77–6.76 (m, 2H, $C_6H_4$); 6.69–6.67 (m, 2H, $C_6H_4$); 5.32 (d, $^2J_{PH}$ = 23.7 Hz, 1H, CHP); 4.41 (broad s, 1H, NH); 3.73 (s, 3H, $CH_3$). $^{31}$P NMR (243 MHz, $CDCl_3$): δ 13.52. $^{13}$C NMR (150 MHz, $CDCl_3$): δ 156.1 ($C_{Ar}$); 153.5 ($C_{Ar}$); 150.5 (d, $^2J_{CP}$ = 9.9 Hz, $POC_{Ar}$); 150.3 (d, $^2J_{CP}$ = 9.8 Hz, $POC_{Ar}$); 139.7 (d, $^2J_{CP}$ = 14.9 Hz, $PCC_{thioph}$); 129.9 (d, $^4J_{CP}$ = 3.0 Hz, $C_{Ar}$); 129.6 ($C_{Ar}$); 128.4 ($C_{Ar}$); 127.4 (d, $^5J_{CP}$ = 2.3 Hz, $C_{5thioph}$); 127.1 (d, $^3J_{CP}$ = 7.6 Hz, $C_{3thioph}$); 126.0 (d, $^4J_{CP}$ = 4.0 Hz, $C_{thioph}$); 125.6 ($C_{Ar}$); 125.5 ($C_{Ar}$); 120.8 (d, $^3J_{CP}$ = 3.7 Hz, $C_{Ar}$); 120.5 (d, $^3J_{CP}$ = 4.3 Hz, $C_{Ar}$); 120.4 ($C_{Ar}$); 116.0 ($C_{Ar}$); 115.5 ($C_{Ar}$); 115.0 ($C_{Ar}$); 55.8 (Ar-$OCH_3$); 53.2 (d, $^1J_{CP}$ = 161.8 Hz, CP). Calculated for $C_{24}H_{22}NO_4PS$: C, 63.85; H, 4.91; N, 3.10; S, 7.10. Found: C, 63.46; H, 5.06; N, 3.04; S, 6.96.

## *2.2. Plant Growth Test of Aminophosphonates 1-6.*

The plant growth test of diphenyl amino(2-thienyl)methylphosphonates **1-6** was performed in laboratory conditions following the OECD 208 Guideline Terrestrial Plants Growth Test [3] for plants: oat (*Avena sativa*) as a monocotyledonous plant and radish (*Raphanussativus L. subvar. radicula Pers.*), a dicotyledonous plant. This method was already successfully used by authors to evaluate toxicity effect of such various aminophosphonic derivatives [1,2,4–7].

According to the mentioned OECD 208 standard, the plant growth test of aminophosphonates**1-6** was carried out in sandy soil having the following parameters: granulometric composition of soil 77% sand, 16% dust and loam, organic carbon content of approx. 1.6%, pH (KCl) equal to 6.6.

Tests were carried out in polypropylene pots (diameter of 90 mm and capacity of 300 $cm^3$), which were filled with the control soil or with the soil mixed with the tested compounds **1-6** added at following concentrations: 100, 200, 400, 800 and 1000 mg/kg of soil dry weight. Each concentration was done in 3 parallel (3 pots for oat, 3 pots for common radish). Twenty seeds of each of the selected plant species were sown into the soil. Seeds of oat have been purchased in Breeding and Seed Company Nieznanice. Seeds of radish were provided by Legutko Ltd company.. Plants were grown for 14 days under controlled conditions: a constant humidity content at the level required for the plants (70% field water capacity), temperature (20±2°C), light intensity (7000 lux) in the system of 16 h/day and 8 h/night.

The evaluation of phytotoxicity of the studied aminophosphonates**1-6** at applied concentrations was made by comparing the germination, dry weight of control plants sprouts (seedlings) with germination and of dry and fresh plants sprouts grown in the soil with an admixture of given amounts of the tested compounds. In order to determine growth inhibition of root and shoot of selected plants, height of shoot and length of root were measured as we described previously [23,24,26-29]. The length of plants is defined as the length the tip of the longest leaf to the base of culms, while root length is measured from the tip of longest root to the root-shoot junction.

Inhibition ratio (IR) was calculated according to method reported by Wang et al. [8] as well as Pawłowska and Biczak [9], namely:

Effective concentration $EC_{50}$ for fresh matter of plants was calculated using GraphPad Prism software (Version 7, GraphPad Software, Inc., La Jolla, CA 92037, USA).

The dry weights of tested plants were measured after drying at 75 °C until the constant weight. Values of the no observed effect concentration (NOEC) and the lowest observed effect concentration (LOEC) of the compounds under study **1-6** was performed. The visual evaluation of phytotoxicity of aminophosphonates**1-6** at applied concentrations was performed by digital photography. Obtained pictures were analyzed in terms of any type of damage to tested plants, such as their growth inhibition, chlorosis, and necrosis. Tests were carried out three times for each sample.

## 2.3. Pigment Assay

Photosynthetic pigments content was determined according to method reported by Oren et al., [10]. Briefly, 200 mg of fresh leaves was thoroughly homogenized in 20 ml of 80% acetone by means of a cooled mortar and centrifuged afterward. The content of chlorophyll a, chlorophyll b and carotenoids was calculated based on the absorbance at wavelength 470, 647 and 664 nm. The content of photosynthetic pigments was expressed in mg/g of dry weight.

## 2.4. Determination of Herbicidal Activity

The weed growth test treated with aminophosphonates **1-6** as a potential soil-applied herbicides was performed using three widely occurring weeds like gallant soldier (*Galinsoga parviflora Cav.*), common sorrel (*Rumex acetosa L.),* and white goosefoot *(Chenopodium album L.*). The weed test was carried out in the same way as the plant growth test and the following concentrations: 100, 400, 1000 mg/kg of soil dry weight.

Herbicidal activity of thiophene derivatives for weeds was determined by growth inhibition of shoot height selected plants and visual assessment of growth inhibition, damages and their drying out, and was documented on digital photographs, which was made on the 21st day of plant growth.

Ratings were assigned based on scales from European Weed Research Council [11–13], wherein for phytointoxication one has 1: total plant death (100%); 2: excellent (98.0 to 99.9%); 3: very good (95.0 to 97.9%); 4: good to acceptable (90 to 94.9%); 5: moderate (82.0 to 89.9%); 6: weak (70.0 to 81.9%); 7: bad (55.0 to 69.9%); 8: very bad (30 to 54.9%); and 9: none (0.0 to 29.9%).

## 2.5. Microtox® Toxicity Assay

Detailed procedure of Microtox® Toxicity Assay has been described previously by us [14]. Method is based on the analysis of light emission reduction of luminescent bacteria (*Aliivibriofischeri*) under toxic stress. The tests were carried out in a Microtox® M500 analyzer according to the 1992 Microtox® Manual. The Microtox® Solid-Phase Test (MSPT) was adopted to report of Doe *et al.* [15].

The MSPT procedure allows the test organisms to come direct contact with an aqueous suspension of the solid test sample. Thus it is possible to detect toxicity which is due to the insoluble solids that are not in the solution. All materials and reagents were purchased from MODERNWATER (New Castle, DE, USA). Toxicity was determined by using the marine luminescent bacterium, *Aliivibrio fischeri*, naturally adapted to a saline environment. Briefly, bacteria were regenerated with 1 mL of Reconstitution Solution (0.01%) and placed in the reagent well of the Microtox®. A suspension of 7 g of the tested soil was prepared in 35 mL of a Solid Phase Diluent (3.5% NaCl) and was magnetically stirred for 10 min. Then a series of dilutions were made and bacteria (approx. $1 \times 10^6$cell/mL per assay) were exposed to these dilutions and to a blank (3.5% NaCl solution) for 20 min. Next, after filtration, the light output of supernatants containing exposed bacteria was measured after 5 min with a Microtox® Analyzer 500. Inhibition was calculated as the concentration of compound loaded to sediment (mg/L) that caused a 50% reduction in the light emitted by the bacteria, and $EC_{50}$ along with 95% confidence limit determined by the software provided with the Analyzer.

## 2.6. Ostracod Test Kit

Ecotoxicity evaluation of synthesized compounds was performed in a short term contact test using Ostracodtoxkit F™ provided by MicroBiotests Inc. (Gent, Belgium). This direct sediment contact bioassay was performed in multiwell test plates using neonates of the benthic ostracod crustacean *Heterocyprisincongruens*hatched from cysts [16].

After 6 days of contact with the tested soil the percentage mortality (calculated the total number of dead ostracods expressed in percent) and the growth of the crustaceans were determined and compared to the results obtained in a non-treated reference soil. Briefly, according to manual of Ostracodtoxkit™ test, the cysts (*Heterocypris incongruens*) were transferred into a Petri dish filled with 10 mL standard fresh water (reconstituted water) and were incubated at 25 °C for 52 h under

continuous illumination (approx. 3000–4000 lux). After 48 h of cysts incubation, pre-feeding of the freshly hatched ostracods was performed with algae (spirulina-powder) provided in the test kit. Next, after hatching, before feeding with algal food suspension, the length measurements of ostracod neonates have been done. Algae (*Selenastrum capricornutum*) used as feed in the test plate were reconstituted according to the manufacturer's procedure. Each well of a test plate was filled in the following order: 2 mL standard freshwater, 2500 μL of sediment (soil) treated and non-treated for comparison (blank), 2 mL already prepared algal suspension, 10 ostracods. The test plates were covered with Parafilm® and closed by a lid. Then multiwell plates were incubated at 25 °C in darkness for 6 days. After 6days of exposure, the ostracods have been recovered from the multiwells to determine the percentage mortality. To calculate the growth inhibition of survived organisms, their length measurements have been also done. Mortality of test organisms was determined in six replicates. The measurement of length was carried out by means of a micrometric strip placed on the bottom of a glass microscope plate. Growth inhibition (GI) of *Heterocypris incongruens*in the test sediment was calculated as follows:

### *2.7. Statistical Analysis*

The significance of the obtained results was evaluated using the analysis of variance (ANOVA). The least significant difference (LSD) values at a confidence level of 95% were computed using the Tukey test. Moreover, the mean standard deviation were determined and plotted in diagrams. Statistical analysis was performed with STATISTICA 13.3 software (version, company, city, country).

## References


1. Lewkowski, J., Malinowski, Z., Matusiak, A., Morawska, M., Rogacz, D., Rychter, P. *The effect of new thiophene-derived aminophosphonic derivatives on growth of* terrestrial plants: a seedling emergence and growth test. *Molecules* **2016**, *21*, 694. DOI: 10.3390/molecules21060694
2. Rogacz, D., Lewkowski, J., Malinowski, Z., Matusiak, A., Morawska, M., Rychter, P. Effect of New Thiophene-Derived Aminophosphonic Derivatives on Growth of Terrestrial Plants. Part 2. Their Ecotoxicological Impact and Phytotoxicity Test Toward *Herbicida*l **App**lic*a*tion in Agriculture. *Molecules* **2018**, *23*, 3173. DOI: 10.3390/molecules23123173
3. Terrestrial plant test: seedling emergence and seedling growth test. In *OECD/OCDE Guidelines for the Testing of Chemicals. Section 2. Effects on Biotic Systems*, Organization For Economic and Cooperation Development (OECD) Publishing, Paris, France, 2006; Test no. 208. http://dx.doi.org/10.1787/9789264070066-en
4. Karpowicz, R., Lewkowski, J., Stasiak, M., Czopor, A., Tokarz, P., Król, A., Rychter, P. Synthesis of novel N-(p-toluenesulfonyl) aminophosphonates and evaluation of their biological properties. *Phosphorus, Sulfur, Silicon Rel. Elem.* **2018**, *193*, 423-436. DOI: 10.1080/10426507.2018.1424712
5. Rychter, P., Rogacz, D., Lewicka, K., Kollár, J., Kawalec, M., Mosnáček, J. Ecotoxicological Properties of Tulipalin A-Based Superabsorbents versus Conventional Superabsorbent Hydrogels. Adv. Polymer Technol. **2019**, Article ID 2947152. DOI: 10.1155/2019/2947152
6. Lewkowski, J., Morawska, M., Karpowicz, R., Rychter, P., Rogacz, D., Lewicka, K., Dobrzyński, P. Evaluation of Ecotoxicological Impact of New Pyrrole-derived Aminophosphonates Using Selected Bioassay Battery. *Ecotoxicology* **2017**, *26*, 914–929. DOI:10.1007/s10646-017-1821-4.
7. Lewkowski, J., Morawska, M., Karpowicz, R., Rychter, P., Rogacz, D., Lewicka, K. Novel (5-nitrofurfuryl)-substituted esters of phosphonoglycine - Their synthesis and phyto- and ecotoxicological properties. *Chemosphere* **2017**, *188*, 618-632. DOI: 10.1016/j.chemosphere.2017.09.031
8. Wang, L.-S., Wang, L., Wang, L., Wang, G., Li, Z.-H., Wang, J.-J. Effect of 1-butyl-3-methylimidazolium tetrafluoroborate on the wheat (*Triticumaestivum*L.) seedlings. *Environ. Toxicol.* **2009**, *24*, 296-303. DOI: 10.1002/tox.20435
9. Pawłowska, B., Biczak, R. Evaluation of the effect of tetraethylammonium bromide and chloride on the growth and development of terrestrial plants. Chemosphere **2016**, 149, 24-33. DOI: 10.1016/j.chemosphere.2016.01.072
10. Oren, A., Kuehl, M., Karsten, U. An endoevaporitic microbial mat within a gypsum crust: zonation of

phototrophs, photopigments and light penetration. *Mar. Ecol. Prog. Ser.* **1995**, *128*, 151–159. DOI: 10.3354/meps128151

11. Johannes, H. Report of the Third and Fourth Meetings of the European Weed Research Council Committee on Methods. *Weed Res.***1964**, *4*, 79. DOI: 10.1111/j.1365-3180.1964.tb00271.x
12. Costa, A.G.F., Sofiatti, V., Maciel, C.D.G., Lira, A.J.S., Cordeiro Jr, A.F., Silva, R.L.M. Weed management with herbicides applied in pre and postemergence on castor crop. *PlantaDaninha***2015**, *33*, 551-559. DOI: 10.1590/S0100-83582015000300017
13. Parsons, D., Lane, P., Hall, E., Galloway, A., Pham Van, B. *Effective Weed Control for Talish and Hairy Canary Clover Seed Crops*; Rural Industries Research and Development Corporation, Barton, Australia, 2013, pp. 4-5.
14. Lewkowski, J.; Rodriguez Moya, M.; Chmielak, M.; Rogacz, D.; Lewicka, K.; Rychter, P. Synthesis, spectral characterization of several novel pyrene-derived aminophosphonates and their ecotoxicological evaluation using *Heterocyprisincongruens*and *Vibrio fischeri*tests. *Molecules* **2016**, *21*, 936, doi:10.3390/molecules21070936.
15. Doe, K., Scroggins, R., Mcleay, D., Wohlgeschaffen, G. Solid-phase test for sediment toxicity using the luminescent bacterium Vibrio fischeri. In *Small-scale Freshwater Toxicity Investigations*, Blaise, C., Férard, J.F., Eds.; Springer, Dordrecht, Netherlands, 2005, Volume 1, pp. 107–136
16. Martínez-Sánchez M.J., Pérez-Sirvent C., García-Lorenzo M.L., Martínez-López S., Bech J., García-Tenorio R., Bolívar J.P. Use of bioassays for the assessment of areas affected by phosphate industry wastes. *J. Geochem. Explor.* **2014**, *147*, 130–138. DOI: 10.1016/j.gexplo.2014.05.019

© 2019 by the authors. Submitted for possible open access publication under the terms and conditions of the Creative Commons Attribution (CC BY) license (http://creativecommons.org/licenses/by/4.0/).
